# PHD1-dependent hydroxylation of RepoMan (CDCA2) on P604 modulates the control of mitotic progression

**DOI:** 10.7554/eLife.108131

**Published:** 2026-06-25

**Authors:** Jimena Druker, Hao Jiang, Dilem Shakir, Fraser Child, Vanesa Alvarez, Melpomeni Platani, Andrea Corno, Constance Alabert, Adrian T Saurin, Jason R Swedlow, Sonia Rocha, Angus I Lamond

**Affiliations:** 1 Division of Molecular Cell and Developmental Biology, School of Life Sciences, University of Dundee Dundee United Kingdom; 2 Department of Biochemistry, Cell and Systems Biology, Institute of Systems, Molecular and Integrative Biology, University of Liverpool Liverpool United Kingdom; 3 Cancer Research, School of Medicine, University of Dundee Dundee United Kingdom; University of Texas Southwestern Medical Center United States; Goethe University Frankfurt Germany

**Keywords:** proline hydroxylation, RepoMan (CDCA2), PHDs, cell cycle, mitotic progression, PP2A, Human

## Abstract

Prolyl-hydroxylases (PHDs) are oxygen-sensing enzymes that mediate the hydroxylation of proline residues. In mammals, three PHD isoforms (PHD1–3) are responsible for proline hydroxylation of hypoxia-inducible factor (HIF) alpha, a key regulator of the hypoxia response. In the accompanying paper (Jiang et al., 2025), we report development of a mass spectrometry-based method to reliably identify proline hydroxylation (OH-Pro) sites on proteins and use this to identify a PHD-dependent OH-Pro modification at Pro604 on the protein RepoMan (CDCA2), a regulatory subunit for protein phosphatase PP1γ with important roles in mitotic progression and cell viability. Here, we investigate the functional significance of hydroxylation of RepoMan at P604. During M phase, the PP1-RepoMan complex dephosphorylates Thr3 of Histone H3 (H3T3) on chromosome arms to ensure the correct localisation of the chromosomal passenger complex (CPC) at centromeres. We show that siRNA depletion of PHD1, but not PHD2, increases H3T3 phosphorylation in prometaphase-arrested cells. In cells depleted of endogenous RepoMan, exogenous expression of wild-type RepoMan, but not a RepoMan-P604A mutant, restored normal H3T3 phosphorylation localisation in prometaphase arrested cells. RepoMan-P604 is located proximal to the short linear motifs (SLiMs) that function as binding sites for the serine/threonine protein phosphatase 2A (PP2A). The interaction of RepoMan and PP2A-B56γ is reduced in cells expressing RepoMan-P604A. Moreover, analyses in both fixed and live cells released from a prometaphase arrest show that expression of the RepoMan-P604A mutant delays completion of mitosis, results in defects in chromosome alignment and segregation, and increases levels of cell death. These data support a role for PHD1-mediated prolyl hydroxylation in controlling progression through mitosis, acting, at least in part, via hydroxylation of RepoMan at P604 regulating the interaction of RepoMan with PP2A during chromosome alignment and thereby controlling the levels of Histone H3 phosphorylation at Thr3.

## Introduction

Proline hydroxylases (PHDs) are part of a large family of 2-oxoglutarate-, iron-, and oxygen-dependent enzymes (2-OGD) (Wilson et al., 2020). PHDs are best known for their role in hydroxylating and controlling hypoxia-inducible factor (HIF) levels in normal oxygen, iron, and 2-oxoglutarate conditions (Kaelin and Ratcliffe, 2008). They are also sensitive to metabolites analogous to 2-oxoglutarate, including succinate and fumarate (Sciacovelli et al., 2016; Laukka et al., 2018), oncometabolites, such as L2-hydroxyglutarate (Laukka et al., 2018), and certain amino acids (Durán et al., 2013). PHDs are thus intricately linked to oxygen and metabolic sensing and control (Wilson et al., 2020).

There are three PHD enzymes in mammals, termed PHD1, PHD2, and PHD3, all of which have HIF hydroxylating functions (Frost et al., 2021). However, PHD2 is the dominant PHD for HIF hydroxylation (Berra et al., 2003), with PHD1 and PHD3 being required for negative feedback loops (Metzen et al., 2005; Pescador et al., 2005). PHD1 is not hypoxia-inducible. Hydroxylation of HIFα results in the formation of a high-affinity binding site for the von Hippel Lindau protein, which is the recognition component of an E3-ubiquitin ligase, targeting HIFα for rapid degradation by the proteasome (Maxwell et al., 1999; Ohh et al., 2000; Epstein et al., 2001; Ivan et al., 2001). Given the importance of PHDs in controlling HIF levels, several PHD inhibitors have been developed by the pharmaceutical industry (Haase, 2021), and these are now approved for use in human patients suffering from anaemia derived from chronic kidney disease (Haase, 2021).

Identification of additional PHD targets, outside the HIF family, has been surrounded by controversy over the last 15 years, confounded by the fact that only PHD2 has a significant phenotype in knockout mice (Takeda et al., 2006). However, recent studies have provided evidence, based upon a combination of genetic, biochemical, molecular, and cellular biological data, identifying additional targets for all PHD enzymes (Batie et al., 2023). When analysing the function of these novel targets, broadly, PHD2 targets seem to relate to signalling and metabolism, while PHD1 targets align to gene regulation and cell cycle (Batie et al., 2023; Druker et al., 2021).

We have developed a robust, mass spectrometry (MS)-based method for the enrichment and identification of proline hydroxylation sites on target proteins, allowing the detection of PHD targets in cell and tissue extracts. In the accompanying manuscript (Druker et al., 2025), we validate this MS approach and use it to identify a set of putative PHD target proteins. We have previously shown that PHD1 contributes to the control of cell cycle progression (Moser et al., 2013; Ortmann et al., 2016). Interestingly, one of the novel PHD1 target proteins identified by Druker et al., 2025, was CDCA2, also known as RepoMan, which is a cell cycle regulated protein phosphatase 1 (PP1) interacting protein (Trinkle-Mulcahy et al., 2006). RepoMan functions as a regulatory subunit for protein phosphatase PP1γ, with important roles in mitotic progression, chromosome architecture, and cell viability (Trinkle-Mulcahy et al., 2006; Vagnarelli et al., 2006). RepoMan localisation to chromatin is regulated during the cell cycle by phosphorylation and dephosphorylation mechanisms, and this is required for proper chromosomal passenger complex (CPC) localisation and mitosis progression (Qian et al., 2013; Qian et al., 2015).

Here, we characterise the functional significance of RepoMan hydroxylation at P604, as identified by Druker et al., 2025. We demonstrate that RepoMan hydroxylation at P604 is sensitive to PHD inhibitors, PHD1 depletion, and increased levels of fumarate. RepoMan-P604 hydroxylation is required for efficient mitotic progression from prometaphase into metaphase with loss of P604 hydroxylation causing delayed mitosis, defects in chromosomes alignment and segregation, and cell death. These results support a critical role for PHD1 in controlling cell cycle progression and expand the repertoire of validated protein targets whose function depends upon site-specific proline hydroxylation.

## Results

### RepoMan interacts with PHD1 in asynchronous cells

**Figure 1.**
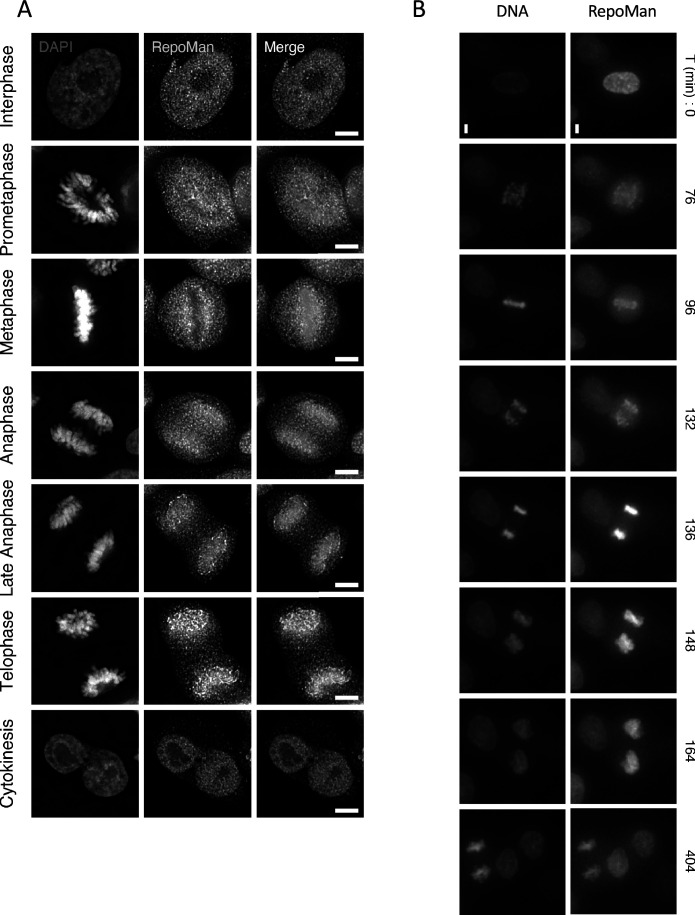
RepoMan localisation across the cell cycle. (**A**) Immunofluorescence analysis of endogenous RepoMan in HeLa cells along the cell cycle. CDCA2 antibody was used to detect endogenous RepoMan. DNA was stained with DAPI. Scale bar represents 5 μm. (**B**) Time lapse of YPF-RepoMan in cells treated with thymidine for 24 hr and release in normal media. DNA was stained with sirDNA. YFP-RepoMan in green and DNA in red. Scale bar represents 5 μm.

Previous studies have shown that RepoMan localises to chromatin during interphase but rapidly dissociates from chromatin and becomes diffusely distributed when cells enter mitosis. Upon anaphase onset, RepoMan loads back onto chromatin and remains there until the next mitosis (Trinkle-Mulcahy et al., 2006; Qian et al., 2013; Qian et al., 2015). First, therefore, we confirmed by immunofluorescence that we could reproduce the previously reported localisation behaviour of endogenous RepoMan throughout the cell cycle, both in fixed HeLa cells (Figure 1A) and in live HeLa cells using YFP-RepoMan (Figure 1B). These results support the previous conclusions and show that RepoMan loads onto chromatin in HeLa cells at anaphase onset and remains associated with chromatin throughout the subsequent interphase, until it dissociates from chromatin when cells enter the next mitosis.

**Figure 2.**
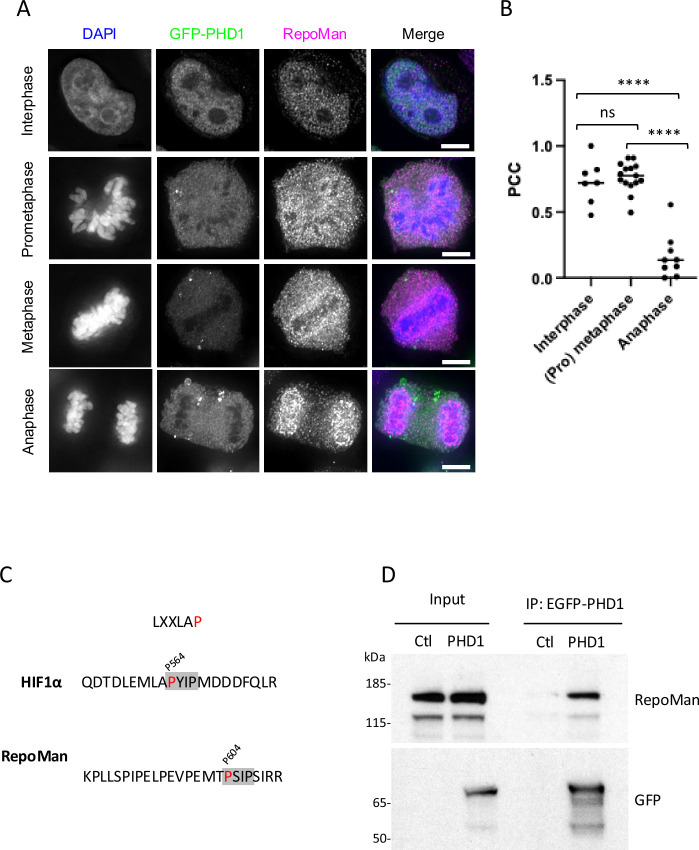
Endogenous RepoMan interacts with EGFP-PHD1. (**A**) Asynchronous HeLa cells were transiently transfected with 1 µg of EGFP-PHD1 expression vector and after 48 hr were fixed with 4% PFA and subjected to immunofluorescence using CDCA2 and GFP antibodies (endogenous RepoMan and EGFP-PHD1, respectively). DNA was stained with DAPI. The images show the localisation of endogenous RepoMan and EGFP-PHD1 along the cell cycle: interphase, prometaphase, metaphase, and anaphase. Scale bars represent 5 µm. (**B**) Colocalisation of GFP-PHD1 with endogenous RepoMan is shown. The graph displays the median of Pearson’s correlation coefficient measured (using Costes’ automatic threshold) in asynchronous cells captured in different stages of the cell cycle (interphase, (pro)metaphase, and anaphase). Unpaired t-test p=0.0531 interphase vs (pro)metaphase, p<0.0001 anaphase vs (pro)metaphase, p<0.0001 anaphase vs interphase. (**C**) Short sequence similarity between HIF1α and RepoMan around prolines 564 and 604 (PYIP, PSIP), respectively. (**D**) Co-immunoprecipitation between EGFP-PHD1 and endogenous RepoMan. Asynchronous HeLa cells were transiently transfected with EGFP-PHD1. After 48 hr cells were lysed and subject to immunoprecipitation using GFP trap magnetic beads and analysing by western blot. Figure 2—source data 1.TIFF files containing full western blots for data presented in Figure 2. Figure 2—source data 2.PDF file containing full western blots for data presented in Figure 2, indicating relevant bands and treatments. Figure 2—source data 3.Data utilised to generate the graph in Figure 2B.

Next, we compared the localisation of RepoMan and PHD1 across the cell cycle in asynchronous cells by analysing the localisation of endogenous RepoMan in HeLa cells transiently expressing EGFP-PHD1, both during interphase and mitosis (Figure 2A). EGFP-PHD1 localised in nuclei during interphase, consistent with previous results showing PHD1 to be bound to promoter regions and involved in hydroxylation of histone H3 (Liu et al., 2022). RepoMan also localised to nuclei during interphase but became enriched around the chromosome periphery during prometaphase (Figure 2A). However, at anaphase onset, RepoMan, as expected, loads onto chromatin, but PHD1 remains diffuse around the segregating anaphase chromosomes (Figure 2A). These results show that the localisation patterns of PHD1 and RepoMan are not identical, consistent with PHD1 acting on multiple different target proteins, not just RepoMan. The main overlap in localisation occurs during interphase and (pro)metaphase, assessed by Pearson’s correlation (Costes et al., 2004), potentially allowing enzyme and substrate to colocalise and interact (Figure 2B).

RepoMan does not possess the consensus motif for proline hydroxylation (LXXLAP) present in HIFα. However, a short sequence next to Pro604 in RepoMan (PSIP) is similar to the PYIP sequence next to Pro564 in HIF1α (Figure 2C). These amino acids are involved in the PHD2-HIF1 oxygen-dependent degradation domain interaction through hydrogen bonds and hydrophobic interaction with residues in PHD2 that are conserved in PHD1 (Chowdhury et al., 2016) and may thus be involved in the RepoMan-PHD interaction we detect in cells. To test this further, we analysed the interaction between RepoMan and ectopically expressed EGFP-PHD1 in asynchronous cultures of HeLa cells. Co-IP experiments, using GFP-Trap, show that endogenous RepoMan is in a complex with EGFP-PHD1 (Figure 2D). Together, these results are consistent with a model in which RepoMan and PHD1 can potentially interact throughout the cell cycle, except during anaphase, suggesting that the hydroxylation of RepoMan-P604 by PHD1 might occur before chromosome segregation.

### Reduction in either PHD1 levels or activity increases H3T3ph on chromosome arms

**Figure 3.**
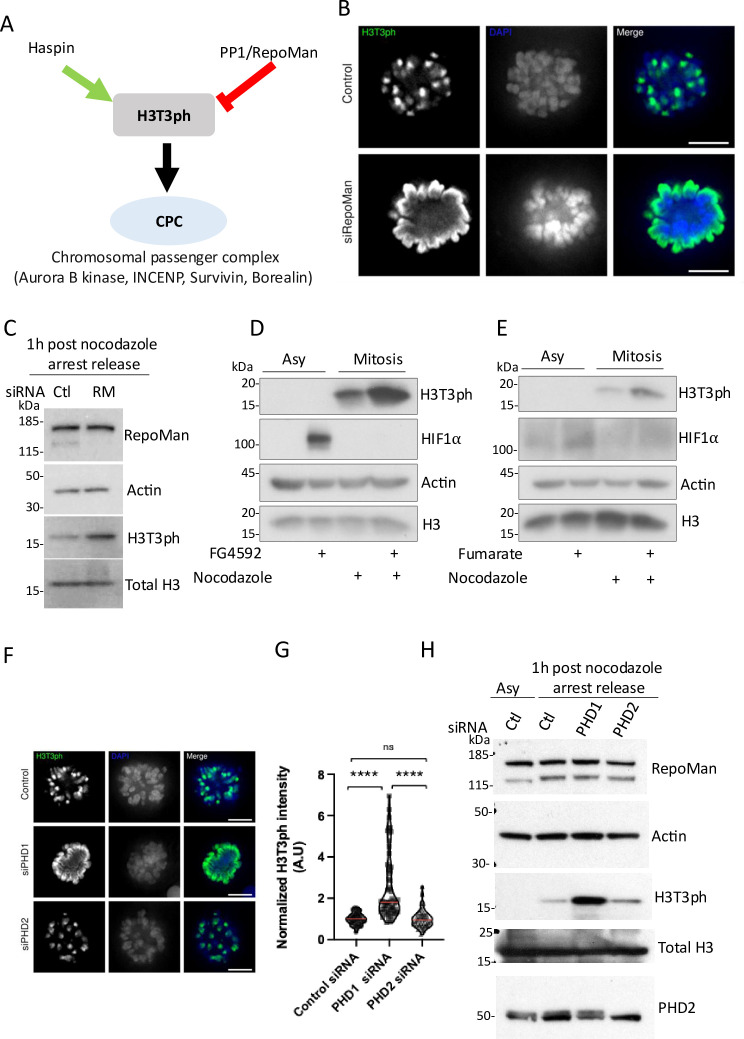
PHD1 regulates phosphorylation of H3T3 during prometaphase. (**A**) Schematic model of the role of PP1-RepoMan during prometaphase. (**B**) HeLa cells were transfected with siControl, or siRNA RepoMan. Cells were arrested in prometaphase with nocodazole 100 ng/mL for 16 hr and released from the arrest for 1 hr in normal media before fixation and stained with pH3T3 and DAPI. (**C**) Cells were treated as in (**B**) and harvested for western blot analysis. (**D**) Asynchronous or prometaphase-arrested HeLa cells were treated with FG4592 50 µM for 2 hr or DMSO. In case of mitotic cells, they were treated 1 hr before the release and during the release for another hour. Cells were lysed and subject to western blot. (**E**) Asynchronous or prometaphase arrested HeLa cells were treated with 100 µM fumarate for 1 hr. In case of mitotic cells, they were treated for 1 hr during the release. Cells were lysed and subject to western blot. (**F**) Immunofluorescence images of HeLa cells arrested in prometaphase. HeLa cells were transfected with siControl, siPHD1, siPHD2 (as in B). Prometaphase-arrested cells were released for 1 hr before fixation. Image scale bars represent 5 µm. (**G**) Graph displays the normalised pH3T3 intensity of total cells per condition from 3 independent experiments (n = 48 si control, n = 62 si PHD1, n = 47 siPHD2). pH3T3 intensity of each condition (siPHD1 and siPHD2) was normalised to the intensity values of pH3T3 in the siControl. The average of 3 independent experiments is shown. Unpair t-test ****p<0.0001, sicontrol vs siPHD1 and siPHD1 vs siPHD2; n.s, sicontrol vs siPHD2 p = 0.8963. (**H**) Western blot analysis of HeLa cells treated as in (**F**) and including asynchronous cells transfected with the control siRNA. Figure 3—source data 1.TIFF files containing full western blots for data presented in Figure 3. Figure 3—source data 2.PDF file containing full western blot for data presented in Figure 3, indicating relevant bands and treatments. Figure 3—source data 3.Data utilised to generate the graph in Figure 3G.

RepoMan functions as a regulatory subunit for protein phosphatase PP1γ, with important roles in mitotic progression and cell viability (Trinkle-Mulcahy et al., 2006). During prometaphase and metaphase, PP1-associated RepoMan dephosphorylates Thr3 of Histone H3 (H3T3) on chromosome arms (Qian et al., 2011). This prevents the recruitment of the CPC and promotes the correct localisation of the CPC complex to centromeres, where H3T3 serves as a docking site (Figure 3A). Proper localisation of the CPC is crucial to ensure correct chromosome alignment and segregation during mitosis (Qian et al., 2013; Qian et al., 2015; Qian et al., 2011; De Munter et al., 2020). Reduction of RepoMan expression by siRNA has been reported to increase H3T3 phosphorylation on chromosome arms in prometaphase cells (Qian et al., 2013; Qian et al., 2015; Qian et al., 2011; De Munter et al., 2020). To determine if this result could be replicated in our experiments, RepoMan was knocked down in HeLa cells using siRNA and H3T3 phosphorylation analysed in nocodazole-arrested cells, by both immunofluorescence (Figure 3B) and western blot (Figure 3C). This confirmed the previous reports that RepoMan depletion coincides with increased H3T3 phosphorylation. Importantly, this result validates the use of H3T3 phosphorylation as a readout of RepoMan function.

**Figure 3—figure supplement 1.**
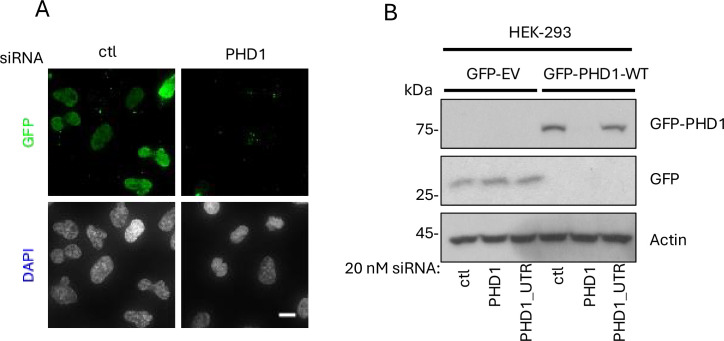
siPHD1 is effective at depleting PHD1 protein. (**A**) Immunofluorescence analysis of siPHD1 knockdown in HeLa cells transiently transfected with EGFP-PHD1. siControl was used as a control. GFP antibody was used for EGFP-PHD1, and DNA was stained with DAPI. Scale bar represents 10 µm. (**B**) Western blot analysis of siPHD1 or si PHD1 UTR knockdown on HEK293 cells stably expressing GFP-PHD1 or GFP empty vector. Figure 3—figure supplement 1—source data 1.TIFF files containing full western blots for data presented in Figure 3—figure supplement 1. Figure 3—figure supplement 1—source data 2.PDF file containing original western blot for Figure 3—figure supplement 1, indicating relevant bands and treatments.

Next, we investigated the potential biological role of RepoMan-P604 hydroxylation, analysing H3T3 phosphorylation during prometaphase. First, H3T3 phosphorylation was analysed after treating cells with the PHD inhibitor, FG4592. This revealed that FG4592 treatment resulted in increased levels of phosphorylated H3T3 in mitotic cells (Figure 3D), as does RepoMan depletion (Figure 3C). These observations are consistent with RepoMan hydroxylation being necessary for RepoMan-dependent dephosphorylation of H3T3 in cells during early mitosis (Figure 3D).

PHD enzymes are known to sense changes in levels of metabolites, as well as oxygen, for example, responding to increased levels of TCA metabolites, such as fumarate (Isaacs et al., 2005). Therefore, we investigated if PHD inhibition by fumarate also impaired the ability of RepoMan to regulate H3T3 phosphorylation. Treatment of HeLa cells with fumarate resulted in increased phosphorylation of H3T3 in mitotic cells (Figure 3E), analogous to that observed after FG4592 treatment. These data suggest that PHD activity is involved in the regulation of H3T3 phosphorylation during prometaphase and further indicate that prometaphase can be modulated in response to changes in levels of metabolites.

While we demonstrate here, and in the accompanying paper by Druker et al., 2025, that PHD1 can hydroxylate RepoMan at P604, we wanted to investigate whether RepoMan may also be a target for PHD2. Therefore, we analysed levels of H3T3 phosphorylation by immunofluorescence, comparing how this was affected by siRNA knockdown of either PHD1 or PHD2. This showed that only depletion of PHD1, but not PHD2, resulted in increased levels of H3T3 phosphorylation in nocodazole-arrested cells, similar to that seen after siRNA-knockdown of RepoMan (Figure 3F and G). Similar results were observed when lysates of nocodazole-arrested cells, which had been depleted of PHD1 by siRNA, were analysed by immunoblotting (Figure 3H). Due to the lack of antibodies that can detect endogenous PHD1, we confirmed the knockdown of PHD1 in HeLa cells, either by immunofluorescence analysis of cells transiently expressing EGFP-PHD1 or by western blot analysis of a HEK293 cell line stably expressing GFP-PHD1 (Figure 3—figure supplement 1).

Taken together, these results indicate that PHD1 is involved in RepoMan regulation and can modulate the levels of phosphorylated H3T3. The data are also consistent with the hydroxylation of RepoMan at P604 being important for its function during early mitosis.

### P604 hydroxylation is necessary for RepoMan function in early mitosis

**Figure 4.**
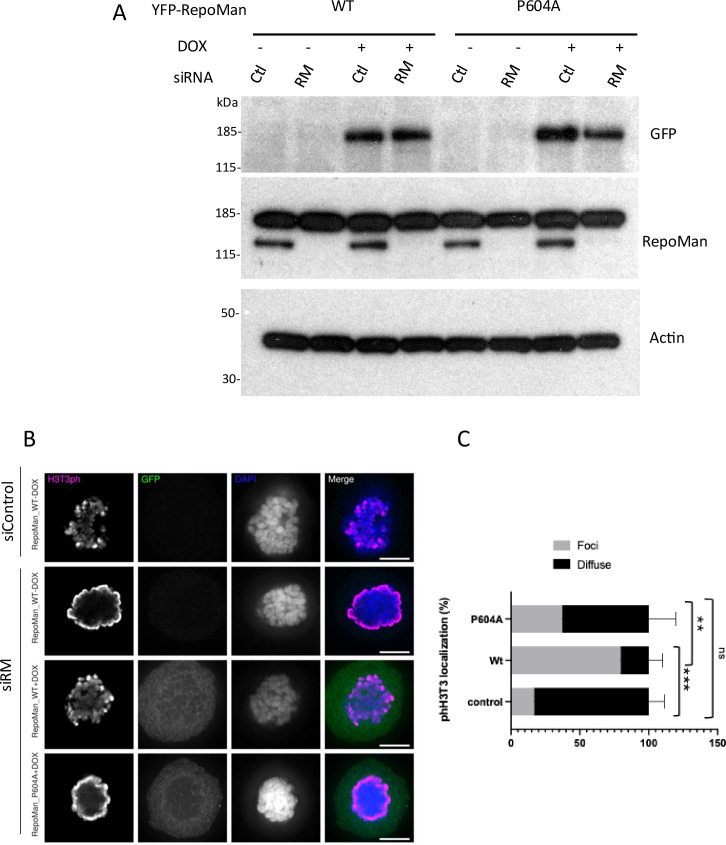
P604 in RepoMan is required for correct localisation and function in mitosis. (**A**) Western blot analysis of HeLa-YFP-RepoMan-wt or the P604A mutant induction in the presence of 1 µg/mL doxycycline. Cells were transfected with the siRNA of RepoMan and after 24 hr cells were induced with doxycycline for another 24 hr before harvested. Blots were developed with GFP, RepoMan, and actin antibodies. (**B**) Immunofluorescence of HeLa-YFP-RepoMan-wt or the P604A synchronised in prometaphase with nocodazole after siRM knockdown. Dox was used to induce the expression of the YFP proteins. Anti phH3T3, GFP antibodies were used, and DNA was stained with DAPI. (**C**) Quantification of (**B**) in cells treated with siRM, with or without doxycycline induction. The graph displays the distribution of phH3T3 on prometaphase cells. Graph represents the percentage of cells showing phH3T3 localisation as foci (centromeric) or diffuse localisation (along chromosome arms). Average of 4 independent experiments with a total number of cells 46 for siRM (not induced), 46 for siRM+ YFPRMwt, and 52 for siRM+YFP-P604A (9–20 cells per condition per experiment). Error bars represent standard deviation (SD). Unpair t-test, control vs wt ***p = 0.0002 ,** wt vs P604A p=0.0087 and n.s , P604A vs control p = 0.1254. Figure 4—source data 1.TIFF files containing full western blots for data presented in Figure 4. Figure 4—source data 2.PDF file containing full western blots images for data presented in Figure 4, indicating relevant bands and treatments. Figure 4—source data 3.Data utilised to generate the graph in Figure 4C.

To investigate further the functional role of RepoMan Pro604 hydroxylation in early mitosis, we generated stable HeLa cell lines that exogenously express either a YFP-tagged wild-type (wt) RepoMan or a YFP-tagged point mutation of RepoMan, in which the hydroxylated proline 604 is replaced with alanine (YFP-RepoMan-P604A). These cell lines are doxycycline-inducible and the YFP-RepoMan expressed is also resistant to siRNAs that deplete endogenous RepoMan, allowing for rescue experiments to be performed. Both the inducible RepoMan-wt and P604A mutant expressed at comparable levels, similar to the levels of endogenous RepoMan (Figure 4A).

**Figure 4—figure supplement 1.**
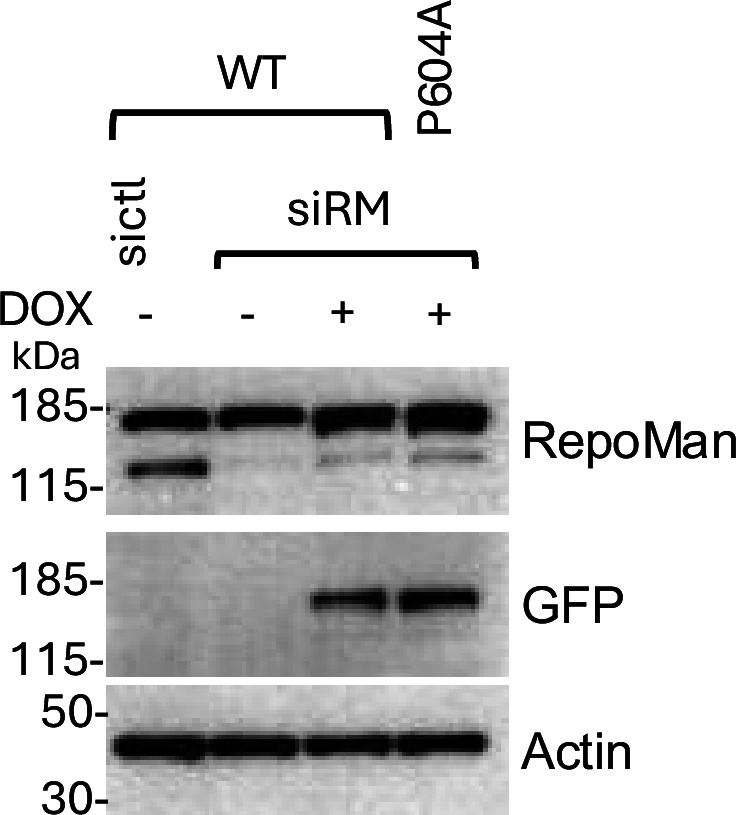
siRepoMan is effective at depleting endogenous but not exogenous RepoMan. HeLa cells were transfected with siRNA control or siRNA against endogenous RepoMan (siRM). 24 hr later, cells were incubated with thymidine 2 mM±doxycycline for 24 hr. After the incubation time, cells were released from the thymidine for 2 hr in normal media and nocodazole±doxycycline was added for 14–16 hr. Cells were lysed, and protein extracts analysed by western blot. Figure 4—figure supplement 1—source data 1.TIFF files containing full western blots for data presented in Figure 4—figure supplement 1. Figure 4—figure supplement 1—source data 2.PDF file containing original western blot for Figure 4—figure supplement 1, indicating relevant bands and treatments.

We analysed by immunofluorescence the localisation and levels of phosphorylated H3T3 to determine if this is affected during early mitosis by expression of the RepoMan-P604A mutant protein. As expected, in control cells transfected with an siRNA control (without doxycycline), phosphorylated H3T3 localises preferentially to centromeres, while cells transfected with siRNA that depletes endogenous RepoMan show phosphorylated H3T3 spread along chromosome arms (Figure 4B and C; Figure 4—figure supplement 1).

The induction of YFP-RepoMan-wt in cells depleted of endogenous RepoMan by siRNA transfection clearly rescued the phosphorylated H3T3 phenotype observed in control cells, i.e., immunofluorescence again shows H3T3 localised preferentially to centromeres. Interestingly, however, induction with doxycycline of the YFP-RepoMan-P604A mutant, in contrast to the YFP-RepoMan-wt, was unable to rescue knockdown of endogenous RepoMan, with H3T3 phosphorylation still showing a similar distribution spread along chromosome arms as seen in cells depleted of endogenous RepoMan (Figure 4B and C; Figure 4—figure supplement 1). We note that this mislocalisation of phosphorylated H3T3, as seen both after siRNA depletion of endogenous RepoMan and in the presence of the exogenous YFP-RepoMan-P604A mutant, is similar to that observed in cells expressing endogenous RepoMan after siRNA-mediated depletion of PHD1 (Figure 3F). Together, these results suggest that hydroxylation of RepoMan at P604 is required for the PP1-RepoMan complex to dephosphorylate H3T3 from chromosome arms during prometaphase.

### P604-RepoMan is required for PP2-B56 interaction during (pro)metaphase

The loading of PP1-RepoMan onto chromatin during mitosis is dynamic and depends on phosphorylation of Ser893 in the histone binding domain of RepoMan (Qian et al., 2015). This residue is phosphorylated by Aurora B and dephosphorylated by a pool of PP2A-B56 that is associated with RepoMan (Qian et al., 2015). As we saw no evidence that lack of hydroxylation on P604 alters levels of RepoMan protein (Figure 5—figure supplement 1A), we sought to determine whether the YFP-P604A RepoMan mutant mislocalised during the cell cycle, as compared with the equivalent YFP-RepoMan-wt protein. To do this, we used live-cell imaging in HeLa cells released from a 24 hr thymidine block (Figure 5—figure supplement 1B), comparing the degree of loading onto chromatin in early mitosis of the respective, exogenously expressed YFP-tagged wt and P604A RepoMan proteins. Both the wt and mutant constructs showed similar levels of loading onto chromatin during (pro)metaphase (Figure 5—figure supplement 1C).

We next compared the association of the YFP-tagged wt and P604A RepoMan proteins with chromatin as cells traversed mitosis by measuring the ratio of RepoMan to DNA signals in cells entering mitosis. We note that the level of RepoMan expression differs between individual cells analysed in this experiment, due to measurements being made with a mixed population of cells, rather than a clone. To account for this, we normalised the values of fluorescence intensity measured throughout mitosis progression, with the intensity of YFP-tagged RepoMan in G2 (T0), for each cell that was measured (Figure 5—figure supplement 1D). This analysis revealed that the YFP-RepoMan-P604A mutant loads onto chromatin almost twice faster than wt, suggesting potential changes to the interaction of RepoMan with B56/PP2A during prometaphase.

**Figure 5.**
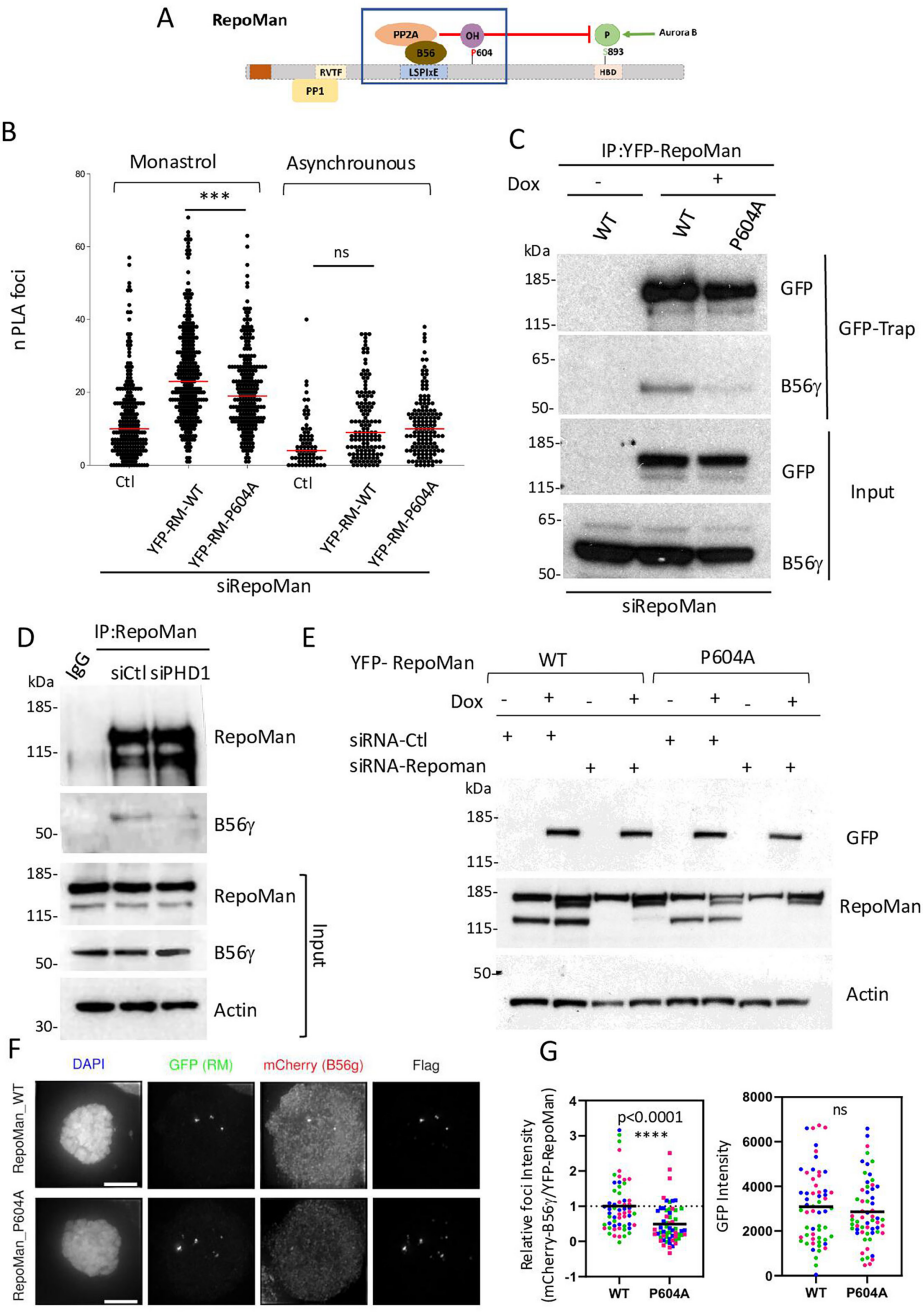
RepoMan-P604 hydroxylation is required for the recruitment of B56γ in prometaphase cells. (**A**) Schematic representation of RepoMan proline 604 proximity to B56-PP2A binding site (LSPIxE). (**B**) Proximity ligation assay (PLA). Graph represents the number of PLA foci per cell in the indicated conditions (monastrol arrested or asynchronous). Quantified PLA foci are located in the YFP area (RepoMan localisation) of the mitotic cells (phSer10 positive cells). Endogenous RepoMan was knocked down using siRM. Median is shown in red. Statistical analysis was performed using an unpaired t-test, and the p-value comparing wt vs mutant under monastrol treatment is <0.0001. Number of cells per condition, in the same order as shown, are: 229, 301, 237, 71, 130, and 139. Before PLA reaction, cells were fixed with PFA 4% and stained with Anti-GFP+B56γ antibodies or only GFP antibody was used as a negative control. After PLA reaction cells were stained with anti-phH3Ser10 as a mitotic marker. (**C**) Co-immunoprecipitation between YFP-RepoMan-wt or P604A mutant with endogenous B56γ. Endogenous RepoMan was knocked down using siRM. 16 hr after transfection, YFP-RepoMan was induced with 1 µg/mL doxycycline and cells were synchronised in prometaphase. Cells lysates were subject to immunoprecipitation using GFP trap magnetic beads and analysed by western blot. (**D**) Co-immunoprecipitation between endogenous RepoMan and endogenous B56γ in cells depleted or not of PHD1 using siRNA. HeLa cells were transfected with siRNA (PHD1 or ctl), 24 hr later cells were incubated with thymidine 2 mM for another 24 hr. After the incubation time, cells were released from the thymidine block, for 2 hr in normal media and arrested in prometaphase with nocodazole for 14 hr. Cells were harvested by shake-off and lysed for immunoprecipitation of endogenous RepoMan. (**E**) Western blot analysis of HeLa-YFP-RepoMan-wt or P604A/mCherry B56γ cell line. Cells were depleted of endogenous RepoMan, and the indicated YFP-RepoMan variants were induced with doxycycline. GFP, CDCA2 (RepoMan), and actin as a loading control were utilised. (**F**) Immunofluorescence showing the recruitment of mCherry B56γ through YFP-RepoMan-wt or P604A at ectopic foci on chr 1 in prometaphase-arrested cells. GFP, mCherry, and Flag antibodies were used to detect YFP, B56γ, and dCas9, respectively. DAPI shows DNA. Graphs represent B56γ levels (**G**) and GFP levels (G). Average of 3 independent experiments, 58 (wt) 60 (P604A) cells. Scale bar represents 5 µm. Mann-Whitney test was applied; p-value<0.0001. Figure 5—source data 1.TIFF files containing full western blots for data presented in Figure 5. Figure 5—source data 2.PDF file containing full western blot images for data presented in Figure 5, indicating relevant bands and treatments. Figure 5—source data 3.Data utilised to generate the graph in Figure 5B. Figure 5—source data 4.Data utilised to generate the graph in Figure 5G.

Phosphorylation of Ser893 on RepoMan, by Aurora B, prevents the loading of RepoMan to chromatin during prometaphase (Qian et al., 2013), with this phosphorylation being removed by the phosphatase PP2A/B56γ (Qian et al., 2013; Qian et al., 2015). The PP2A/B56γ binding site on RepoMan occurs in the short linear motif (SLIM) (Wang et al., 2016) that is located 13 amino acids away from Pro604 (Figure 5A). We therefore hypothesised that the hydroxylation of Pro604 on RepoMan could affect the interaction with B56γ during prometaphase and thus promote the loading of the PP1-RepoMan complex to chromatin, leading to the dephosphorylation of H3T3 on chromosome arms. It has been reported that the interaction between B56γ and RepoMan takes place during mitosis, reaching a maximum at prometaphase (Qian et al., 2015).

**Figure 5—figure supplement 1.**
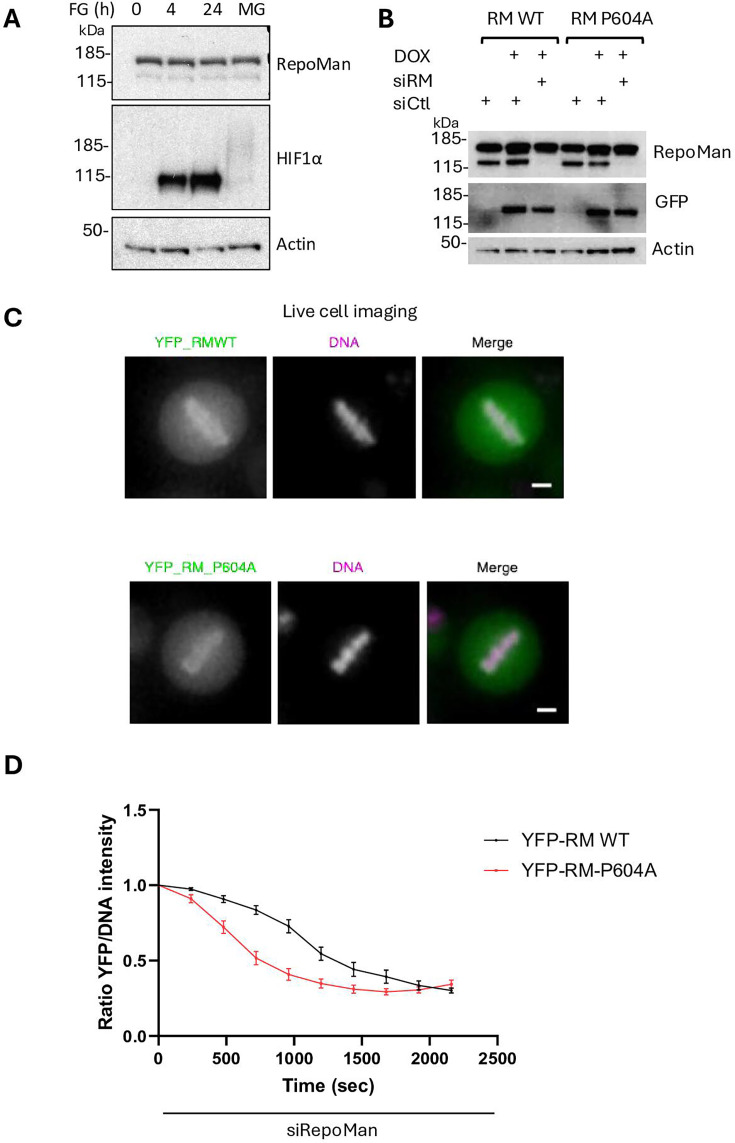
Wild-type (wt) and P604A mutant RepoMan can load to chromatin. (**A**) Western blot analysis of endogenous RepoMan levels in the presence of prolyl-hydroxylases (PHD) inhibitor FG4592 50 µM for the indicated times. DMSO was used in time 0 as a control for 24 hr. MG132 20 µM was added for 3 hr. CDCA2, HIF1α, and Actin antibodies were used. (**B**) Cells were transfected with siRNA control or siRM. 16 hr later, cells were incubated with thymidine 2mM±doxycycline to induce YFP constructs or not (siCtl – dox) for 24 hr. After the incubation time, cells were released from the thymidine block for 6 hr in normal media. Cells were lysed and analysed by western blot. (**C**) Both RepoMan-wt and P604A mutant can load to chromatin during pro(metaphase). Live-cell images of YFP-RepoMan-wt (upper) or P604A mutant in metaphase. Cells were depleted of endogenous RepoMan with siRNAs. Cells were incubated with thymidine plus doxycycline for 24 hr and released in fresh media+doxycycline. Before imaging DNA was stained with sirDNA for 20 min in L15 media, after the incubation time, the media containing sirDNA was removed and cells were incubated in L15 media for cell imaging. Scale bar represents 5 µm. (**D**) Graph depicting tracking of YFP-RepoMan-wt or P604A mutant over chromatin during mitosis. Cells were treated as in (**C**). The graph represents the ratio between RepoMan/DNA intensity over mitosis, values of 2 independent experiments (total number of cells between 2 experiments: RepoMan-wt 29 and RepoMan P604A 24). The values were normalised to the RepoMan/DNA intensity during interphase 2 frames before cells enter in mitosis. Error bars represent SEM. Figure 5—figure supplement 1—source data 1.TIFF files containing full western blots for data presented in Figure 5—figure supplement 1. Figure 5—figure supplement 1—source data 2.PDF file containing original western blot for Figure 5—figure supplement 1, indicating relevant bands and treatments.

**Figure 5—figure supplement 2.**
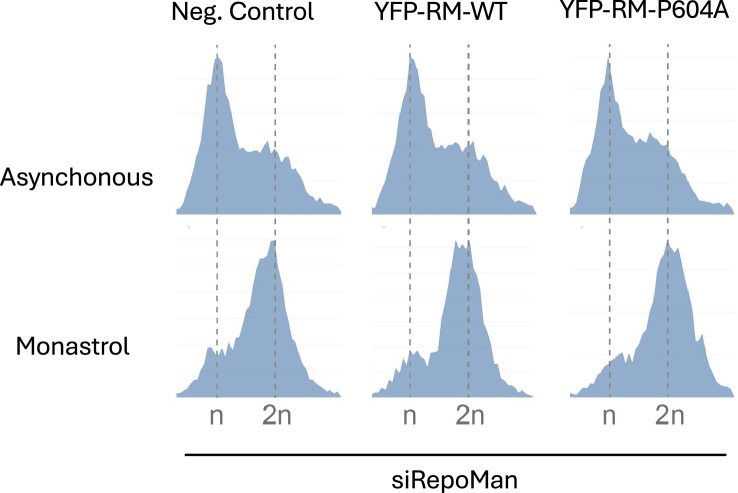
Wild-type (wt) and P604A RepoMan cells are synchronised by monastrol. Cell cycle distribution of the experiment shown. The number of cells is represented in the y axis and DAPI content in the x axis, from n (G1) to 2n (G2/mitosis), marked with dashed lines. First line of graph shows asynchronous cells and the graph on the bottom shows monastrol-treated cells. The whole cell population from each well was analysed. The number of cells per sample: asynchronous 3668, 7725, 7621; monastrol 5089, 5197, 6820. One representative experiment is shown.

**Figure 5—figure supplement 3.**
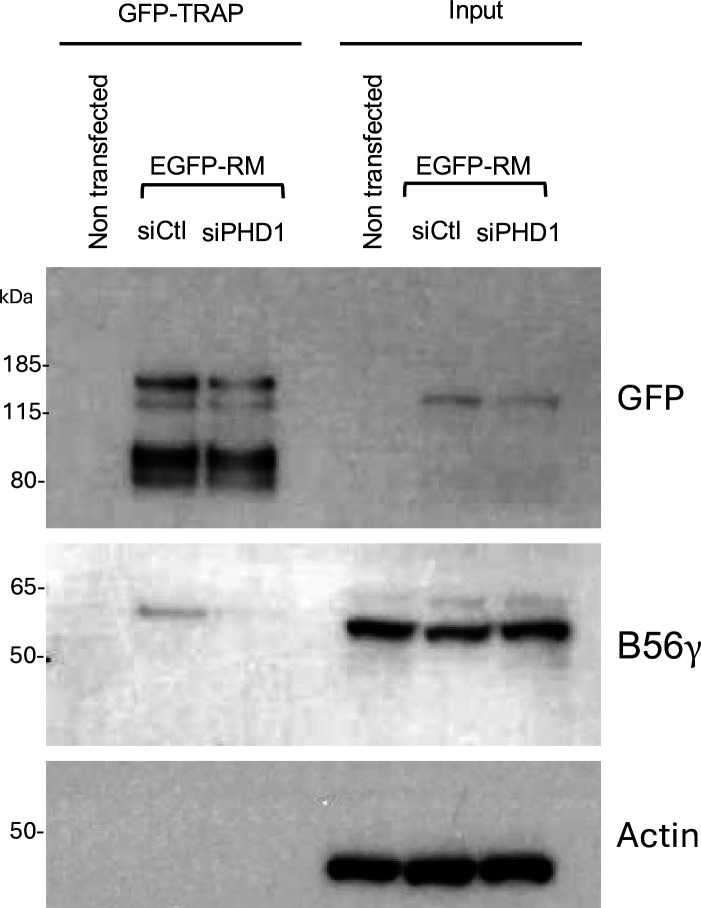
PHD1 is required for the interaction between GFP-RepoMan and endogenous B56γ in mitosis. HeLa cells were transfected with siRNA (PHD1 or ctl). Next day cells were transfected with 5 µg of EGFP-RepoMan for 4 hr. After the incubation time, cells were incubated with thymidine 2 mM for 24 hr, released for 2 hr and incubated with nocodazole 100 ng/mL for 14 hr. Cells were harvested by shake-off and resuspended in lysis buffer. Lysates were subjected to GFP Trap IP, and the interaction of overexpressed EGFP-RepoMan with endogenous B56γ was analysed. Figure 5—figure supplement 3—source data 1.TIFF files containing full western blots for data presented in Figure 5—figure supplement 3. Figure 5—figure supplement 3—source data 2.PDF file containing original western blot for Figure 5—figure supplement 3, indicating relevant bands and treatments.

To test whether the hydroxylation of P604 on RepoMan alters its interaction with PP2A-B56γ during prometaphase, we performed PLA (proximity ligation assay), in either asynchronous HeLa cell cultures or cells synchronised in prometaphase with monastrol (an inhibitor of Eg5), using anti-GFP and anti-B56γ antibodies to detect YFP-RepoMan and endogenous PP2A-B56γ, respectively. As shown by DAPI content (n=G1 and 2n=G2/mitosis), the cell cycle synchronisation procedure was successful for both YFP-RepoMan-wt and the YFP-RepoMan-P604A mutant (Figure 5—figure supplement 2).

In prometaphase cells, YFP-RepoMan-wt shows a higher number of PLA foci, as compared with cells expressing the YFP-RepoMan-P604A mutant (Figure 5B). This is consistent with the YFP-RepoMan-P604A mutant having a reduced interaction with B56γ. To further validate this finding, co-immunoprecipitation experiments were performed, using GFP-Trap beads and cells synchronised with nocodazole in prometaphase, comparing expression of YFP-RepoMan-wt and the YFP-RepoMan-P604A mutant. While cells expressing the wt and mutant RepoMan constructs synchronised equally well (Figure 5—figure supplement 2), the YFP-RepoMan-P604A mutant showed a reduced interaction with endogenous B56γ, as compared with the YFP-RepoMan-wt protein (Figure 5C). In addition, in cells depleted of PHD1, endogenous RepoMan had a reduced interaction with endogenous B56γ (Figure 5D). Similar results were also observed with EGFP-RepoMan and endogenous B56g (Figure 5—figure supplement 3).

We validated this important finding further, using an orthogonal approach. For this, cells were generated that expressed either YFP-RepoMan-wt or the YFP-RepoMan-P604A mutant (both constructs being doxycycline-inducible and resistant to siRNA targeted to endogenous RepoMan) in cells that also constitutively express mCherry-B56γ. Western blot analysis of cell lysates transfected with either siRNA non-targeting control or siRNA targeted to knock down endogenous RepoMan demonstrates that the expression levels of both exogenous YFP-RepoMan-wt and YFP-RepoMan-P604A proteins are comparable and similar to the level of endogenous RepoMan in these cells prior to knockdown (Figure 5E).

Next, we compared the interaction between YFP-RepoMan-wt and YFP-RepoMan-P604A, with mCherry-B56γ-wt on the telomere of chromosome 1, using the dead Cas9 (dCas9)-DARPin system (Corno et al., 2023). Cells were transiently co-transfected with dCas9-DARPin-Flag and sgChr1 expression vectors, following the depletion of endogenous RepoMan with siRNA. Both dCAS9-DARPin and the YFP-RepoMan constructs are doxycycline-inducible. Cells were arrested in prometaphase with nocodazole and fixed for immunofluorescence analysis (see Materials and methods). The GFP panel represents YFP-RepoMan, while the flag panel represents dCas-9-DARPin (Figure 5F). Upon recruitment of mCherry-B56γ, strong foci are seen when YFP-RepoMan-wt is induced (Figure 5F, upper row). However, in cells where YFP-RepoMan-P604A mutant is induced, the intensity of the foci is clearly reduced (Figure 5F, lower row). Quantification of the foci intensity (i.e. the B56γ/YFP ratio) confirms that there is a lower level of interaction between YFP-RepoMan-P604A and mCherry-B56γ in comparison with YFP-RepoMan-wt (Figure 5G). Importantly, the YFP levels in foci are similar for expression of both the YFP-RepoMan-wt and YFP-RepoMan-P604A mutant, indicating that the difference observed in interaction with mCherry-B56γ is not due to differences in the level of YFP recruited to DARPin (Figure 5G).

Overall, these results indicate that proline 604 in RepoMan is important for the interaction between B56γ and RepoMan during prometaphase and support a model in which the hydroxylation of RepoMan-P604 favours interaction with B56γ in cells during prometaphase.

### Loss of RepoMan-P604 hydroxylation results in mitotic defects

**Figure 6.**
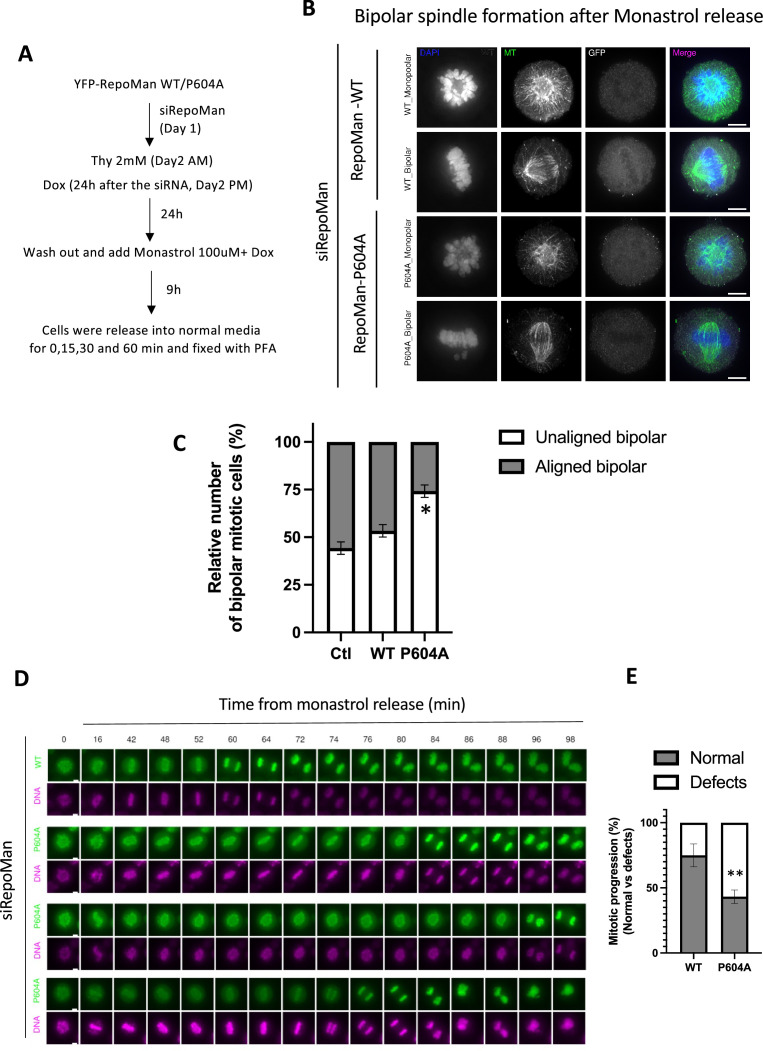
P604 of RepoMan is required for normal mitotic progression. (**A**) Schematic representation of the experimental design. (**B**) Representative immunofluorescence images of YFP-RepoMan arrested in prometaphase with monastrol. Cells were released for 30 min. Monopolar or bipolar cells are shown for either RepoMan-wt or P604A. Cells were fixed and stained with MT and GFP antibodies, and DAPI shows the DNA. Scale bar represents 5 µm. (**C**) Quantification of chromosome alignment in bipolar spindles after release of 1 hr from monastrol arrest. The graph represents the percentage of cells with aligned or unaligned chromosomes in bipolar spindles with respect to the total number of bipolar mitotic cells from 3 independent experiments. Total cells analysed: Ctl 160, RM-wt 196, RM P604A 187. Error bars represent SEM. Unpaired t-test unaligned wt vs mut p=0.0105. (**D**) Time lapse of YFP-RepoMan-wt or P604A cells arrested in prometaphase with monastrol and released into fresh media. (YFP-RepoMan) green and DNA (magenta). Representative images are shown. Scale bar represents 5 μm. (**E**) The graph shows the percentage of normal vs defective mitosis over the total mitotic cells per condition per experiment. (Total number of cells analysed: 57 cells RepoMan-wt and 55 cells for RepoMan-P604A from 3 independent experiments). Error bars represent SD. Unpaired t-test, p=0.0058, defects wt vs mut. Figure 6—source data 1.Data utilised to generate the graph in Figure 6C. Figure 6—source data 2.Data utilised to generate the graph in Figure 6E.

Dephosphorylation of H3T3 by RepoMan-PP1 is essential for correct chromosome alignment and segregation (Qian et al., 2011; Vagnarelli et al., 2011). Since we show above that hydroxylation of RepoMan-P604 by PHD1 is important for efficient dephosphorylation of H3T3 during prometaphase, we analysed if chromosome alignment is affected by hydroxylation of RepoMan-P604A. To address this, we performed a release from monastrol arrest (see Materials and methods), which analyses correct establishment of spindle bipolarity and chromosome alignment (Mayer et al., 1999). First, HeLa cells were arrested in prometaphase with monastrol (Figure 6—figure supplement 1), then released in fresh media and fixed at different time points after release and analysed by immunofluorescence (Figure 6A). Cells expressing either YFP-tagged RepoMan-wt or RepoMan-P604A can form bipolar spindles after monastrol release (Figure 6B). However, the numbers of unaligned chromosomes observed are higher in bipolar cells expressing the YFP-RepoMan-P604A mutant, 1 hr after release from monastrol arrest (Figure 6C). Expression of YFP-RepoMan-P604A resulted in nearly 75% of cells displaying mitotic defects, a significant level compared to cells expressing YFP-RepoMan-wt.

**Figure 6—figure supplement 1.**
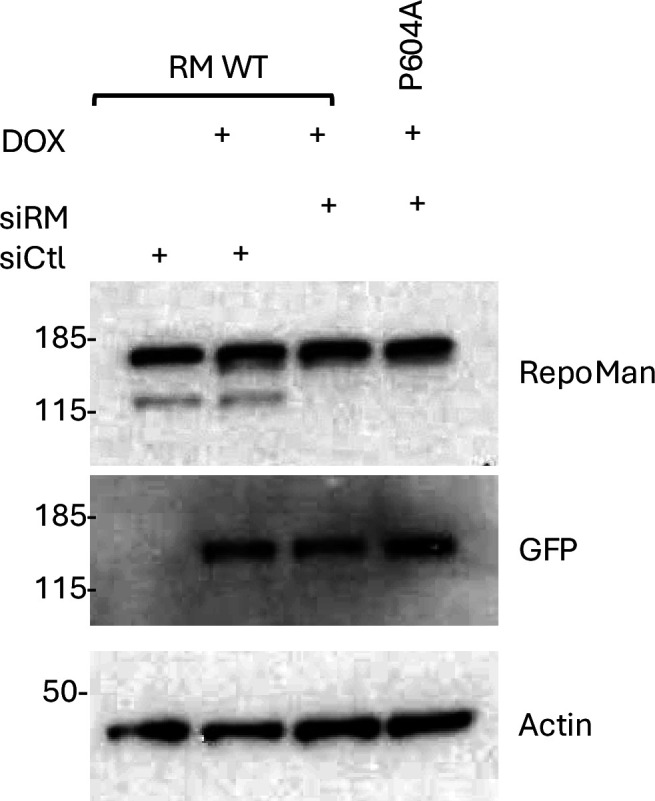
Efficient depletion of endogenous RepoMan and rescue with exogenous RepoMan-wt and P604A. HeLa cells were transfected with siRNA control or siRM. 16 hr later, cells were incubated with thymidine 2 mM±doxycycline to induce YFP constructs or not (siCtl – dox) for 24 hr. After the incubation time, cells were released from the thymidine block for 9 hr in media containing monastrol with or without doxycycline as indicated. After the incubation time, cells were lysed and extracts analysed by western blot. Figure 6—figure supplement 1—source data 1.TIFF files containing full western blots for data presented in Figure 6—figure supplement 1. Figure 6—figure supplement 1—source data 2.PDF file containing original western blot for Figure 6—figure supplement 1, indicating relevant bands and treatments.

Using the same approach, we analysed mitosis progression in live cells. Time-lapse analysis was performed in HeLa cells that had been arrested with monastrol in prometaphase (T=0) and then released into fresh media. On average, anaphase onset in cells expressing YFP-RepoMan-wt occurred 60–70 min after release from monastrol. However, in cells expressing the YFP-RepoMan-P604A mutant, there was an increase in cells failing to complete mitosis, while most of those cells that completed mitosis after monastrol release did so more slowly, showing on average a delay of 15–30 min compared with cells expressing YFP-RepoMan-wt (Figure 6D). Moreover, in cells expressing the YFP-RepoMan-P604A mutant, a variety of defects were apparent. For example, >55% of cells displayed chromosome alignment and segregation problems, including an increased level of cell death (Figure 6D and E).

Taken together, these results support a model in which the hydroxylation of RepoMan at P604 is required for RepoMan function to ensure efficient progression through mitosis.

## Discussion

In the accompanying study by Druker et al., 2025, we have identified by MS that the PP1 regulatory subunit, RepoMan (CDCA2), is hydroxylated at P604 by PHD1, both in cell lines and in vitro. Here, we have used a combination of cellular, biochemical, and fluorescence imaging assays to analyse the potential functional significance of site-specific hydroxylation of RepoMan at P604. We have compared the effect of expressing wt RepoMan with expressing a single-site mutation that replaces proline 604 with alanine. In addition, we have compared the effect of expressing the RepoMan-P604A mutant in cells with siRNA knockdown of PHD1 in cells expressing wt RepoMan. Collectively, these experiments showed that replacing wt RepoMan in cells with a RepoMan-P604A mutant resulted in major defects in early mitosis. These defects included mislocalisation of phosphorylated H3T3, along with decreased interaction with the B56 protein, which is an important targeting subunit for the PP2A-B56 phosphatase complex that dephosphorylates the PP1-RepoMan phosphatase to promote its loading onto chromatin prior to anaphase onset. Furthermore, in cells expressing the RepoMan-P604A mutant, a variety of defects were apparent, including problems with chromosome alignment and segregation and increased cell death, as previously seen in cells expressing endogenous RepoMan after depletion of PHD1 (Moser et al., 2013).

**Figure 7.**
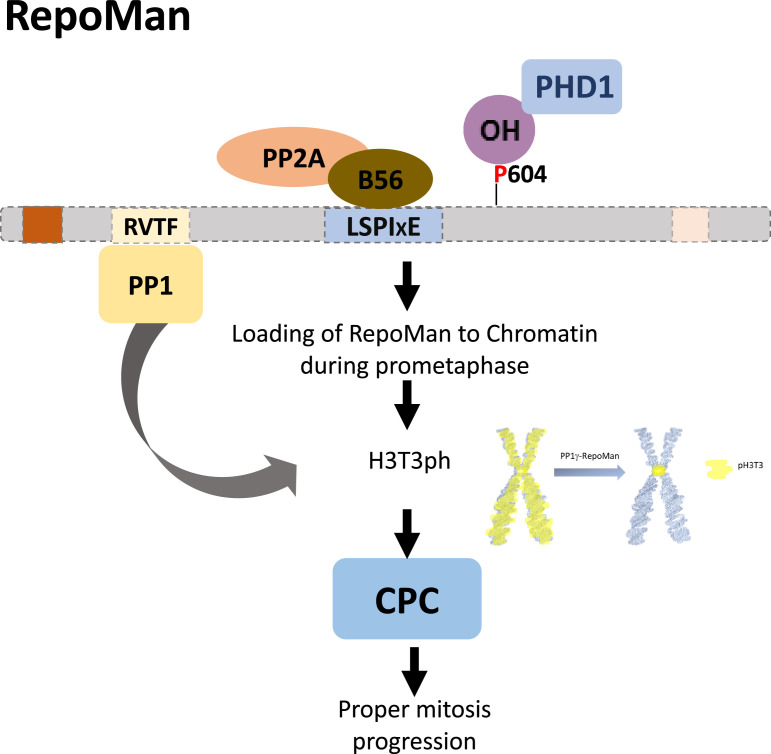
Schematic model of RepoMan hydroxylation during prometaphase. PHD1 hydroxylates RepoMan at proline 604. During prometaphase, this modification is important for the binding of PP2A-B56γ to RepoMan. PP2A-B56γ has a crucial role in the loading of RepoMan to chromatin during prometaphase, leading PP1-RepoMan to dephosphorylate phH3T3 from chromosome arms. The resulted enrichment on the phH3T3 at the centromere assures the correct localisation of the CPC (chromosomal passenger complex) important for the proper mitosis progression.

In combination, the data summarised above lead us to propose the model shown in Figure 7. In this model, the PP1-RepoMan phosphatase complex associates with chromatin throughout the cell cycle, except during prophase when cells enter mitosis. During prometaphase, PHD1-mediated, site-specific hydroxylation of RepoMan at P604 (OH-P604) contributes to stabilising the interaction between the PP1-RepoMan and PP2A-B56 phosphatase complexes, thereby allowing PP2A-B56 to dephosphorylate RepoMan at S893, which in turn allows re-loading of PP1-RepoMan onto chromatin. We propose that when the PP1-RepoMan complex is bound to chromatin, it dephosphorylates phH3T3 on chromosome arms, which aids in targeting the CPC to preferentially bind to centromeres, where higher levels of phH3T3 remain and act as docking sites for the CPC. The PP1-RepoMan phosphatase complex then remains associated with chromatin throughout the remainder of mitosis and the following interphase. Entry into the next mitotic cycle sees the activation of Aurora B kinase, which phosphorylates RepoMan on Ser893 (Qian et al., 2013) and prevents its association with chromatin during prometaphase.

To the best of our knowledge, the model shown in Figure 7 is consistent with all experimental observations, both from our current work and from previous studies by ourselves and others, concerning the dynamic localisation of the RepoMan protein during the cell cycle and its role in mechanisms controlling progression through mitosis (Trinkle-Mulcahy et al., 2006; Vagnarelli et al., 2006; Qian et al., 2015; Vagnarelli et al., 2011; Vagnarelli, 2014; de Castro et al., 2017; Manzione et al., 2020). Importantly, the data here and in the accompanying manuscript by Druker et al., 2025, use MS to identify unambiguously the site-specific hydroxylation of RepoMan at P604 by PHD1 and then characterise the functional significance of this post-translational modification for protein interactions involved in mechanisms important for efficient control of mitotic progression. These new data support and extend our previous observations that PHD enzymes contribute to the regulation of cell cycle progression (Druker et al., 2021; Moser et al., 2013; Ortmann et al., 2016). Furthermore, these data identify specific molecular mechanisms whereby both oxygen-sensing mechanisms and the concentration of specific metabolites, such as fumarate, can influence the cell cycle by affecting the activity of PHD enzymes and changing their ability to hydroxylate proline residues on novel target proteins whose modification state affects their functions in cell cycle control. This provides a new paradigm for understanding mechanisms that can integrate cell cycle regulation in cells and tissues with signals reporting on exposure of organisms to stress and other physiological cues important for homeostasis.

Interestingly, PHD-dependent proline modification can also be modulated in concert with other post-translational protein signalling mechanisms, including phosphorylation (Batie et al., 2023). Of note, P604 is next to T603, which has been shown to be phosphorylated in mitosis in previous phosphoproteomic screens (Dephoure et al., 2008; Kettenbach et al., 2011). It is therefore possible that this phosphorylation might be impacted by P604 hydroxylation. However, additional work is needed to investigate this further.

One interesting aspect of this work is the specificity for PHD1 over PHD2. For HIF regulation, PHD2 is dominant, with PHD1 and PHD3 having lesser roles (Berra et al., 2003). As our knowledge of PHD-targeting and specificity is still based on HIF, more analysis is necessary to really understand how individual PHDs are controlled and select their targets. One possibility is direct modification of PHD enzymes themselves, e.g., by phosphorylation. Phosphorylation of PHD1 by CDKs could alter target selectivity between HIF and Cep192 for example (Ortmann et al., 2016); however, more work is needed to fully understand the regulation of these enzymes in cells.

We note that, in addition to proteins involved in cell cycle regulation, our MS analyses of PHD target proteins has also identified sites of proline hydroxylation in many new proteins that have roles in other important cellular processes, notably including RNA processing (Druker et al., 2025). It will be interesting in future, therefore, to pursue the functional characterisation of these novel PHD targets, to help deepen an understanding of the breadth of cellular mechanisms in which post-translational modification of proteins via proline hydroxylation can modulate function.

There has been a degree of controversy regarding the physiological relevance of new PHD targets identified in cells, beyond HIFα, due, at least in part, to the reported lack of activity of PHDs in vitro towards hydroxylation of non-HIF1 peptides (Cockman et al., 2019). Nonetheless, evidence has been growing from a multitude of laboratories in support of functional roles for site-specific proline hydroxylation by PHDs on protein targets other than HIF1α (Wilson et al., 2020; Batie et al., 2023), e.g., Histone H3 (Liu et al., 2022) and Beclin (Wang et al., 2024) for PHD1, and AMPK (Jiang et al., 2023) and IRF3 (Liu et al., 2024) for PHD2. The data in this study clearly show the functional importance of PHD1-dependent hydroxylation of RepoMan for efficient mitotic progression, providing an important example that PHD targets are not confined to only HIF1α.

## Materials and methods

### Cell culture

HEK293-GFP, HEK293-GFP-PHD1, HeLa, HeLa Flp-in cells, HeLa YFP-RepoMan-wt siRNA-resistant (Cordeiro et al., 2020), and HeLa mCherry-B56γ (Corno et al., 2023) were cultured in Dulbecco’s Modified Eagle Medium (Gibco, # 41966-029) supplemented with 10% Fetal Bovine Serum (FBS, Gibco # A3169801), 100 U/mL penicillin and streptomycin (PS), and 2 mM L-Glutamine. Cell lines were maintained at 37°C with 5% CO_2_ in a humidified incubator. During fluorescence time-lapse analysis, cells were cultured in Leibovitz’s L15 media (Gibco #21083-027) supplemented with FBS and PS. HeLa Flp-in and HeLa mCherry-B56γ cells were used to stably express doxycycline-inducible constructs after transfection with the relevant pcDNA5/FRT/TO vector and the Flp recombinase pOG44 (Invitrogen). Cells were then selected for stable integrants at the FRT locus using 200 µg/mL hygromycin B (Roche) for 2 weeks. A pool of cells generated (rather than a single clone) was used in the experiments in this study.

### Cell cycle synchronisation

Unless indicated otherwise, a prometaphase arrest was induced by culturing cells consecutively for 24 hr with 2 mM thymidine, 2 hr without thymidine and either 16 hr with 100 ng/mL nocodazole or 6–9 hr with 100 µM monastrol. The arrested cells were harvested by shake-off for biochemical analysis. Doxycycline (1 µg/mL; Sigma-Aldrich) was used to induce expression of the YFP-RepoMan constructs during and after the thymidine block, after 16–24 hr of siRNA knockdown to deplete endogenous RepoMan.

### Reagents

Final concentrations used: Doxycycline 1 µg/mL, Nocodazole 100 ng/mL (Sigma #487928), Monastrol 100 µM (Torsis #1305), Thymidine 2 mM (Sigma # T1895), Roxadustat (FG-4592, Selleck # S1007), Hygromycin B (200 µg/µL) (Roche #10843555001), Sir DNA far-red (1:10,000) from Spirochrome SC007. Poly-L-lysine solution (Sigma # P4707) was used to coat coverslips for immunofluorescence.

### Plasmids and mutagenesis

pEGFP-N1_PHD1 (Addgene #21400). dCas9-DARPin-Flag and pU6-sgChr1 (Corno et al., 2023), pcDNA5-YFP-RepoMan-wt expressing an siRNA-resistant and N-terminally YFP-tagged wt RepoMan (Cordeiro et al., 2020). This vector was used to generate pcDNA5-YFP-RepoMan^P604A^ using the site-directed mutagenesis kit (Q5#E0552S NEB), with the following primers: Forward 5’- TGAGATGACACGTTCCATTCCGAG-3’, Reverse 5’-GGGACTTCAGGCAGCTCG-3’ (IDT).

#### Interference RNA transfection

Double-stranded interference RNA (siRNA) was transfected at a final concentration of 20–30 nM using the RNAi max Transfection Reagent (Invitrogen), according to the manufacturer’s instructions, in a six-well plate. siControl, siPHD1, and siPHD2 sequences were described in Moser et al., 2013. RepoMan (5’-UGACAGACUUGACCAGAAA-3’). All siRNAs were obtained from Eurofins genomics and synthesised with a dTdT overhang. For all experiments with either HeLa-YFP-RepoMan-wt or the HeLa-YFP-RepoMan-P604A mutant, the endogenous RepoMan mRNA was first knocked down using siRNA, allowing replacement with the doxycycline-inducible, siRNA-resistant RepoMan constructs.

#### DNA transfection

HeLa cells were plated in either 6 cm or 10 cm plates, 24 hr before transfection. Cells were transfected with either 1 µg or 5 µg of plasmid DNA EGFP-PHD1 using JetPRIME reagent (ratio 1:2) (Polyplus # 114-07), according to the manufacturer’s instructions, in media without antibiotic. 4 hr after transfection, the media was changed to complete media, and after 48 hr further incubation cells were harvested in lysis buffer. For siRNA treatment, HeLa cells were co-transfected in six-well plates with the siRNA and 16 hr later with 1 µg of plasmid DNA (EGFP-PHD1) for another 48 hr (Figure 3—figure supplement 1A). Cells were split to coverslips after siRNA, and DNA was transfected. For Figure 5F, DNA was transfected with FuGene (Promega), as described below.

#### Immunoprecipitation

Asynchronous or prometaphase-arrested HeLa cells were resuspended in 200 µL of lysis buffer (50 mM Tris pH 7.5, 150 mM NaCl, 1% NP40, 0.5% sodium deoxycholate, and 0.1% SDS), containing protease inhibitors (Roche, Complete Mini EDTA-Free) and phosphatase inhibitors (PhosSTOP, Roche). Lysates were incubated on ice for 20 min and cleared by centrifugation at 4°C for 15 min at 13,000×*g*. Supernatant was diluted in 300 µL of dilution buffer (50 mM Tris pH 7.5, 150 mM NaCl); 10% of material was taken as input. 500 µL of the diluted lysate was transferred to a new tube containing pre-washed GFP-Trap magnetic agarose beads (ChromoTek gtma-20) and incubated for 2 hr at 4°C. After the incubation time, beads were washed three times with a washing buffer (50 mM Tris pH 7.5, 150 mM NaCl, and 0.05% NP40) and transferred to a clean tube after the last wash. Beads were resuspended in LDS sample buffer (Invitrogen), heated at 70°C for 10 min, and the eluted proteins were transferred to a new tube. For immunoprecipitation of endogenous RepoMan, lysates were incubated with 1.5 µg of CDCA2 antibody (or IgG as a control) ON at 4°C. Antibody-antigen complex was incubated with magnetic Dynabeads A beads (Invitrogen #10001D) for 2 hr at 4°C. Eluted proteins were analysed by western blot.

### Western blot and dot blot

Western blot analysis was performed in NuPAGE 4–12% Bis-Tris gels (Invitrogen) or normal acrylamide gels. Immobilon-P transfer membranes (PVDF, 0.45 µM pore size, Millipore) were used for a semi-dry transfer. After blocking in 10% fat-free milk in TBS-Tween, membranes were incubated overnight with the primary antibody. After three washes with TBST plus one with TBS for 5 min each, membranes were incubated with the HRP secondary antibodies and developed using Pierce-ECL western blotting substrate (Thermo Scientific #32106).

### Antibodies

The following primary antibodies were used for either western blot or IF, as indicated.

CDCA2 (HPA030049, Sigma) 1/1000 WB, 1/300 IF, Phospho Histone 3 (Thr3) (13576S, Cell Signaling) 1/3000 IF, Phospho Histone 3 (Thr3) (07-424, Millipore) 1/1000 WB, Histone 3 (9715S, Cell Signaling) 1/1000 WB, Actin (3700S, Cell Signaling) 1/5000 WB, PHD2/Egln1 (4835S, Cell Signaling) 1/1000 WB, HIF-1α (610959, BD Biosciences) 1/1000 WB, PP2A-B56γ (A11) (sc-374379, Santa Cruz Biotechnology) 1/1000 WB, 1/100 PLA, GFP rabbit IgG fraction (A11122, Invitrogen) 1/100 PLA, GFP (Ab13970, Abcam) 1/1000 IF, GFP (47859600, Roche) 1/1000 WB. mCherry (GTX 128508, GeneTex) 1/1000 IF, αTubulin, clone DM1A (T9026, Sigma) 1/500 IF, Flag M2 (F3165, Sigma) 1/2000 IF. Fluorescently labelled secondary antibodies for immunofluorescence were obtained from Jackson ImmunoResearch (1/250) and Invitrogen (1/1000) A488 goat anti-chicken (A11039), A568 goat anti-rabbit (A11036), and A647 donkey anti-mouse (A31571). For western blot, HRP secondary antibodies were used at 1/5000 anti-rabbit and anti-mouse IgG-HRP linked (7074S and 7076S Cell Signaling), respectively.

### Proximity ligation assay

Cells were transfected in six-well plates with siRepoMan using Lipofectamine RNAimax (#13778-150 invitrogen). 14 hr later, cells were split in media containing 2 mM thymidine and 1 µg/mL doxycycline (to induce the expression of the YFP constructs) and transferred to a 96-well plate clear base (Greiner 655090) pre-coated with poly L-Lysine solution (sigma #P4707). After 24 hr with thymidine, cells were washed once with PBS and released in media containing 1 µg/mL doxycycline and 100 µM monastrol for 9 hr. Cells were fixed with 4% PFA for 10 min at room temperature (RT) and washed with PBS. Cells were incubated with 3% BSA in PBS plus 0.1% Triton for 30 min at RT and washed with PBS. From this point, fixed cells were subject to Duolink PLA Fluorescent protocol according to the manufacturer’s instructions (Sigma DUO92008), with some modifications. Briefly, cells were incubated with 40 µL/well blocking solution 1× (DUO82007) for 45 min at 37°C in a humidified chamber and then incubated overnight at 4°C with the primary antibody (anti-GFP (1/100) plus anti-B56γ antibody diluted 1/100, or only with anti-GFP (diluted 1/100) as a negative control) in Duolink antibody diluent. After overnight incubation at 4°C in a humid chamber, samples were washed two times with buffer A at RT for 5 min. Anti mouse and anti-rabbit ±probes were added 1:5 in Duolink antibody diluent and incubated for 1 hr at 37°C. After two washes with buffer A at RT, the ligase was added for 30 min at 37°C, washed twice at RT with buffer A, and polymerase together with the red fluorescent reagent was added for the amplification reaction for 100 min at 37°C. After incubation, cells were washed at RT for 10 min with buffer B, 10 min with DAPI 1/1000 in PBS, 10 min with buffer B and 1 min with 1/100 buffer B. After PLA, phSer10 antibody and secondary Alexa Fluor 647 were added to ensure that quantified cells were mitotic cells. We got similar results avoiding this step.

High-throughput microscopy images were taken and analysed with ScanR High Content Screening Microscopy (Olympus). PLA foci per cell were detected with the foci detection package. Data shown in this work correspond with the PLA signal detected in the YFP area of each cell, which corresponds to the RepoMan signal. In addition, cells were filtered by phospho-Serine 10 positive signal. DAPI signal was used to classify cells according to their cell cycle stage. Data were visualised and statistically analysed in Tableau and GraphPad Prism. The statistical test was unpaired t-test.

### dCas-9-DAR protein-protein interaction assay

The interaction of either YFP-RepoMan-wt or YFP-Repoman-P604A mutant with mCherry-B56γ was investigated using a previously described interaction assay (Corno et al., 2023). Experiments were performed by transfecting YFP-RepoMan (wt or P604A) mCherry B56γ cells with dCas9-DARPin-Flag and pU6-sgChr1 (ratio 1:3) using Fugene (Promega) in a six-well plate. 24 hr later, cells were split and transfected with siRepoMan RNA before replating cells in 24-well plates on coverslips. 16 hr later, cells were incubated with 2 mM thymidine plus 1 µg/mL doxycycline for 24 hr. After incubation, cells were released from the thymidine block in media containing 3.3 µM nocodazole and 1 µg/mL doxycycline for 8 hr and fixed with 4% PFA at RT for immunofluorescence. Cells were stained with antibodies to detect YFP-RepoMan, mCherry-B56γ, and DARpin-Flag. Only mitotic cells that show a clear spot of YFP-RepoMan were imaged and quantified.

### Immunofluorescence

Cells on High Precision 1.5H 12 mm coverslips (Merienfield) were fixed with 4% PFA in PBS for 10 min at RT. Fixed cells were washed three times with PBS and incubated with 3% BSA in PBS+Triton 0.1% for 30 min at RT. Coverslips were incubated overnight at 4°C with primary antibody in PBS+3% BSA. After three washes with PBS, coverslips were incubated for 2–3 hr at RT in the dark with secondary antibody plus DAPI (D9542, Sigma 1/1000) in PBS+3% BSA. After incubation, coverslips were washed three times with PBS for 10 min at RT. Each coverslip was mounted on slides using Prolong gold antifade reagent (Invitrogen #P36930). All images, apart from those shown in Figure 3—figure supplement 1A, were acquired on a Deltavision fluorescence microscope (Applied Precision, Inc, Issaquah, WA, USA) using a 100× objective, 1×1 binning, and processed using SoftWoRx software. Images shown in Figure 3—figure supplement 1A were similarly acquired on a Deltavision fluorescence microscope but using a 60× objective. Figure panels were created using OMERO (Allan et al., 2012).

### Time-lapse analysis

Cells were plated in iBIDi µslide eight-well plates and imaged in L-15 media, using a DeltaVision system equipped with a heated 37°C chamber, with a 40× objective, 4×4 binning, 256×256 image size, 4 z-stacks of 5 µm each (total thickness 20 µm), using softWoRx software (Applied Precision, Issaquah, WA, USA). DNA was stained using siRDNA a live-cell far red fluorescent dye (Spirochrome, #SC007). Before adding the monastrol, after the thymidine block, cells were incubated with sirDNA 1/10,000 for 20 min, then the media was replaced with media containing monastrol for the indicated time. Cells were released from the arrest and immediately imaged. For experiments shown in Figure 5—figure supplement 1C and D, after cells were released from thymidine, sirDNA was added for 20 min before imaging.

### Quantitative analysis of immunofluorescence and chromatin loading

For immunofluorescence data, the following macro was run in Fiji to measure background-corrected nuclear intensity in two channels. Individual nuclei were identified at user-selected points of interest by auto-thresholding using the triangle algorithm <<REF-GB3>> followed by ‘Fill holes’. A user-selected region of interest (ROI) was used to estimate background and calculate background-corrected total intensity for both channels, as well as normalised intensity of the second channel relative to the nuclear stain. A second macro was used to prepare data for tracking after deconvolution using SoftWoRx: (1) images were cropped to remove border artefacts, (2) for each timepoint, an average projection over in-focus Z slices was performed, and (3) background subtraction was performed using a sliding paraboloid with radius = 50 pixels. A final macro was used to track nuclei semi-automatically and extract intensity data for loading analysis. Single Z-section time series were tracked starting from a user-defined rectangular selection containing a single nucleus of interest. At each timepoint, a nucleus selection was identified by Otsu auto-thresholding <<REF-GB4>>, fill holes, and optional watershed to split touching objects (a cost function based on area and position changes determines whether the watershed operation is beneficial). Parameters used were: minimum and maximum nucleus area (45–500 µm^2^) and maximum frame-to-frame displacement (20 pixels, 12.68 µm) corresponding to ~0.05 µm/s. The nucleus selection was used as the ROI to measure intensity statistics in a second channel corresponding to Repo-Man protein. Analysis of image data was carried out using an ImageJ <<REF-GB1>>macros in Fiji <<REF-GB2>>.

For quantification of GFP-PHD1 and RepoMan colocalisation, Pearson’s correlation was calculated using the established method by Costes et al., 2004, and the JACoP plugin macro for Fiji (Bolte and Cordelières, 2006), using Costes automatic threshold.

For quantification of images shown in Figure 5F, an ImageJ macro was designed to threshold and select the Chrl foci automatically and to measure the mean foci intensity of mCherry-B56 relative to YFP-RepoMan, as previously described in Corno et al., 2023.

## Data Availability

All data generated or analyzed during this study are included in the manuscript and supporting files; source data files have been provided.

## Additional files

MDAR checklist
