# Supplementary figures and images for: PHD1-dependent hydroxylation of RepoMan (CDCA2) on P604 modulates the control of mitotic progression
Source: eLife. 2026 Jun 25;14:RP108131. doi: 10.7554/eLife.108131 (PMC13299607; doi:10.7554/eLife.108131)

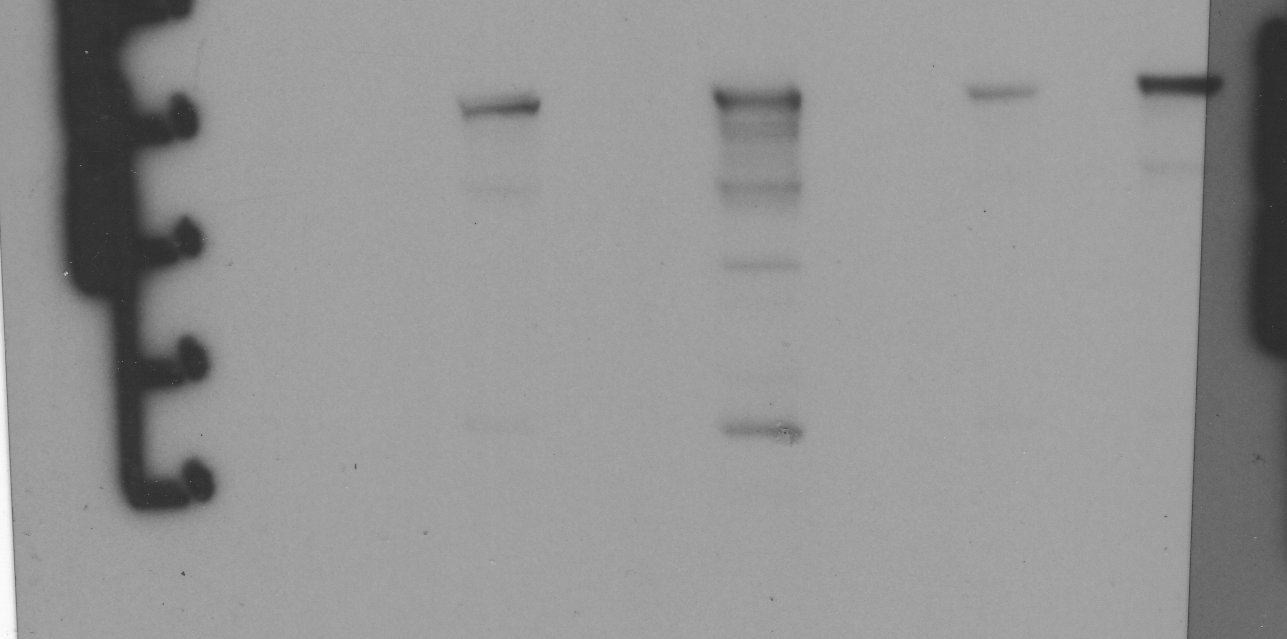

Supplement: Figure 2—source data 1. [file elife-108131-fig2-data1.zip › Figure 2-source data 1/Figure 2 -source data 1-PHD1.tiff]

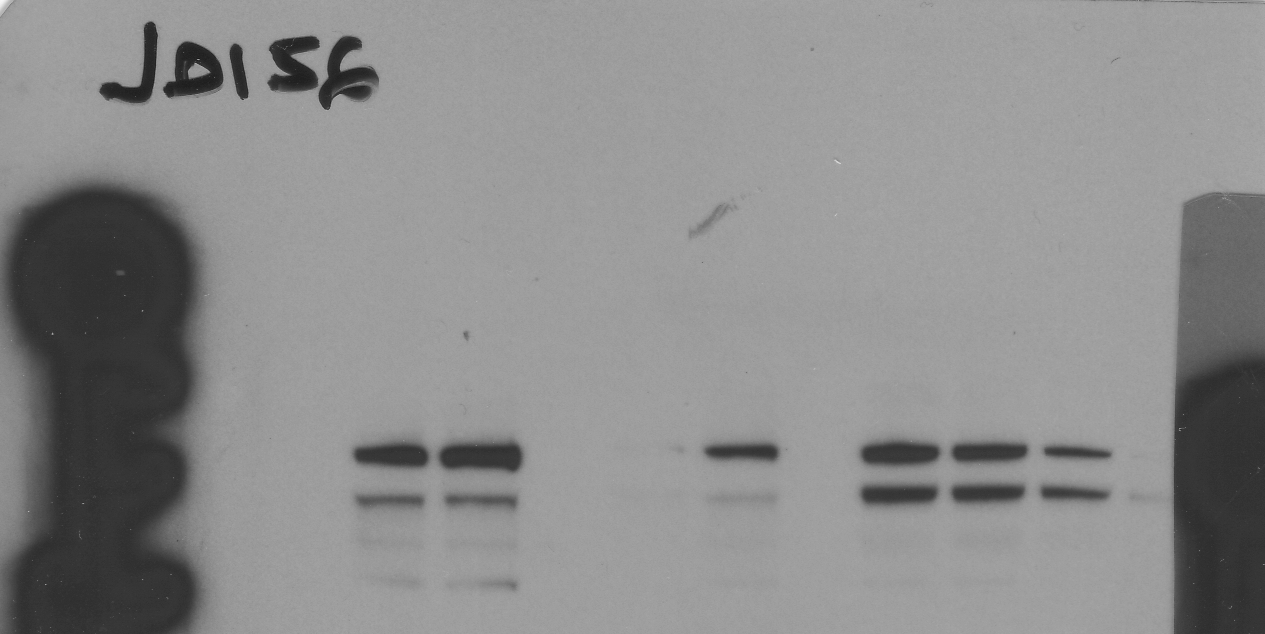

Supplement: Figure 2—source data 1. [file elife-108131-fig2-data1.zip › Figure 2-source data 1/Figure 2 D- source data 1-RepoMan.tiff]

Figure 2-source data 2

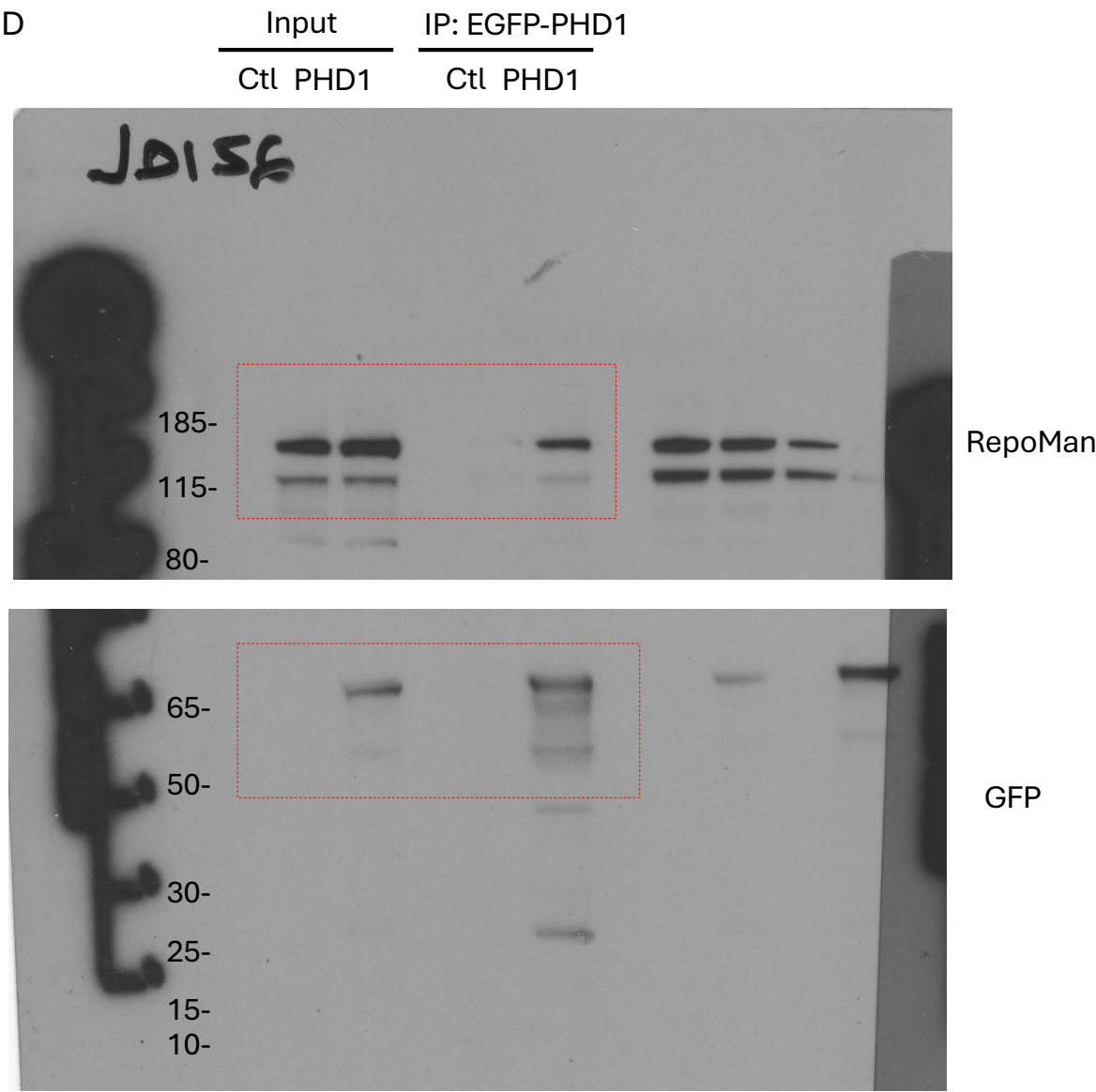

Supplement: Figure 2—source data 2. [file elife-108131-fig2-data2.pdf]

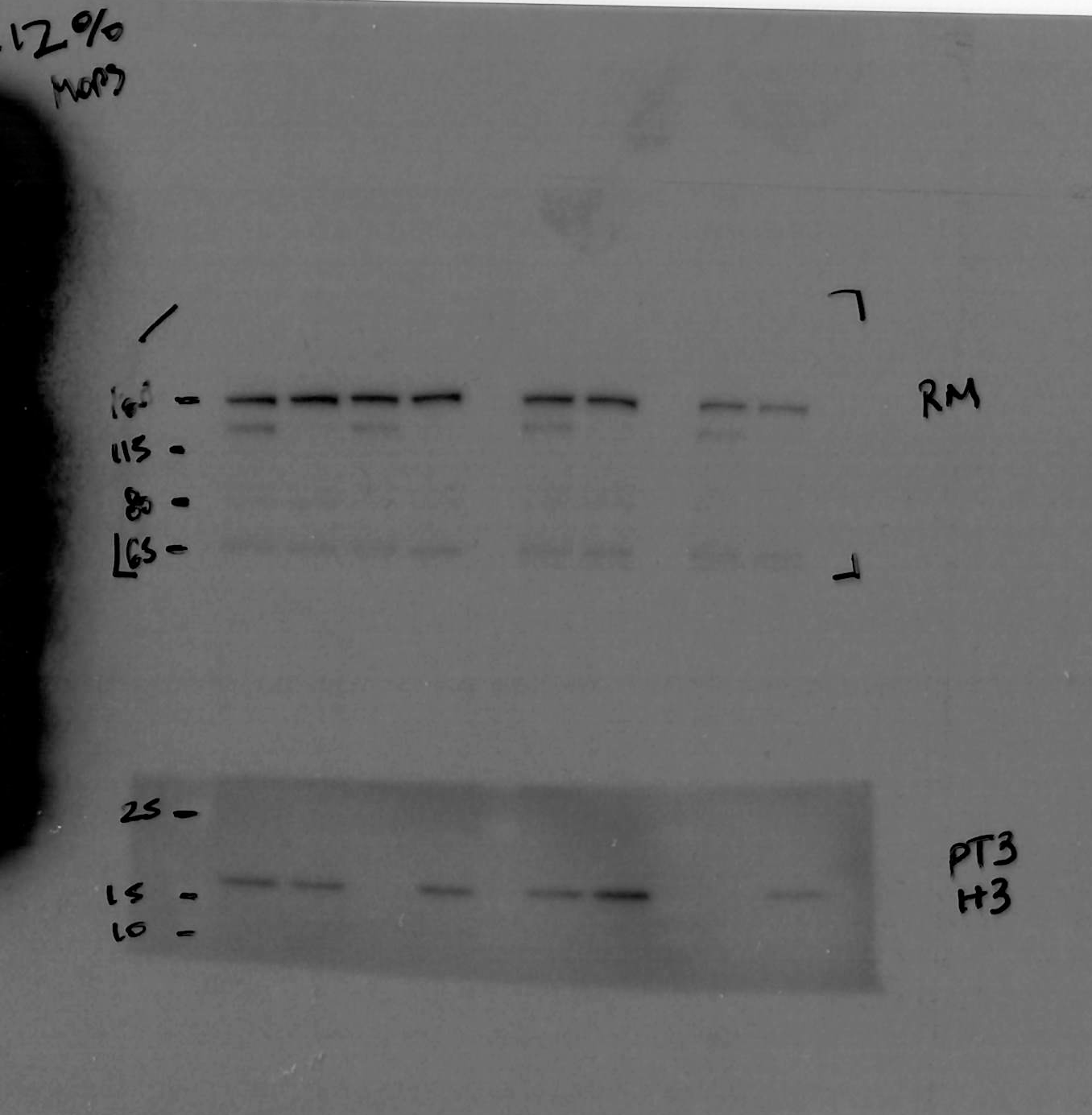

Supplement: Figure 3—source data 1. [file elife-108131-fig3-data1.zip › Figure 3 source data 1 (originals)/Figure 3-source data 1_C/Figure 3C RepoMan_ pH3T3.tiff]

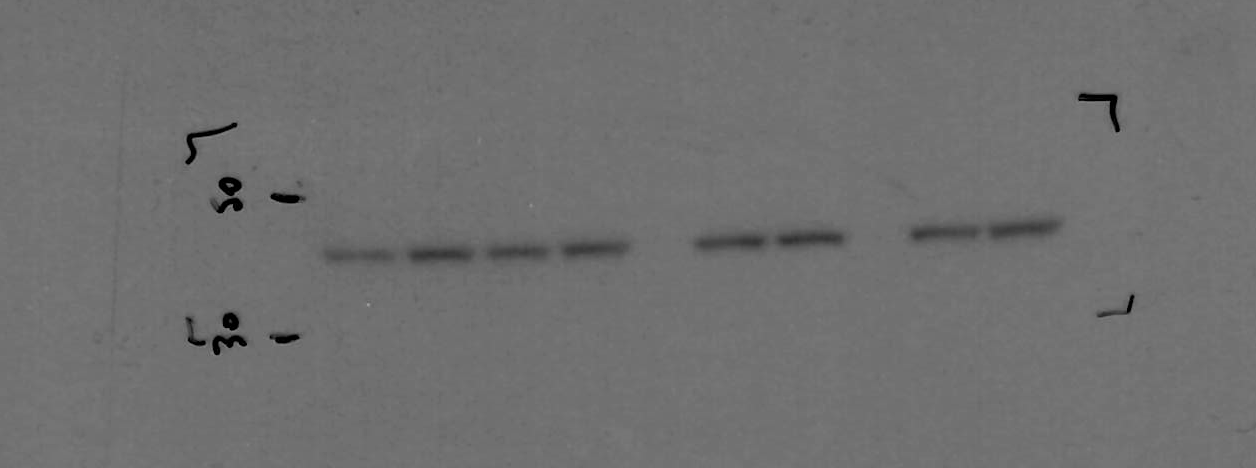

Supplement: Figure 3—source data 1. [file elife-108131-fig3-data1.zip › Figure 3 source data 1 (originals)/Figure 3-source data 1_C/Figure 3C_ Actin.tiff]

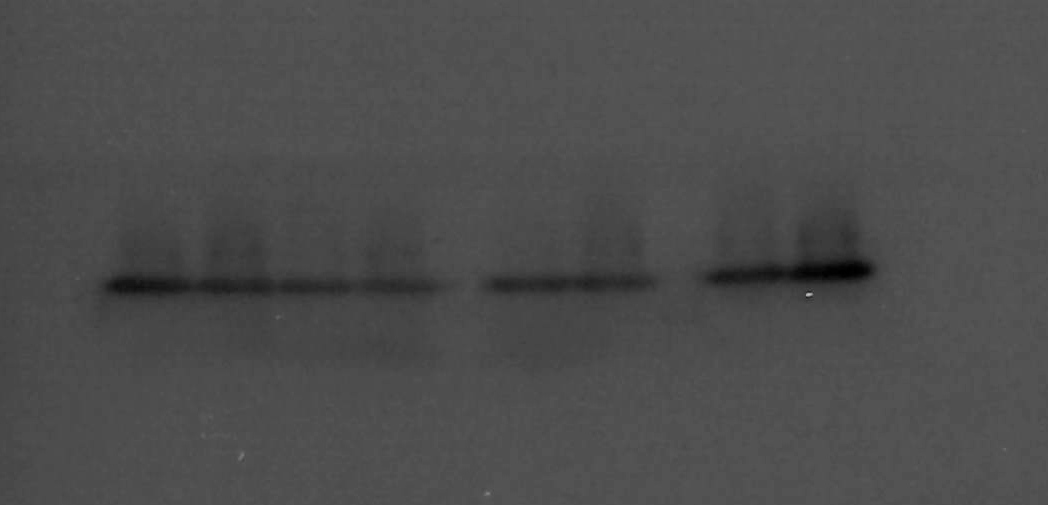

Supplement: Figure 3—source data 1. [file elife-108131-fig3-data1.zip › Figure 3 source data 1 (originals)/Figure 3-source data 1_C/Figure 3C_ tot H3.tiff]

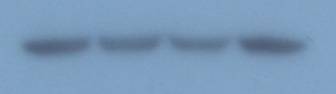

Supplement: Figure 3—source data 1. [file elife-108131-fig3-data1.zip › Figure 3 source data 1 (originals)/Figure 3 source data 1_ E/Actin_Fumarate.tif]

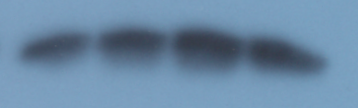

Supplement: Figure 3—source data 1. [file elife-108131-fig3-data1.zip › Figure 3 source data 1 (originals)/Figure 3 source data 1_ E/H3_Fumarate.tif]

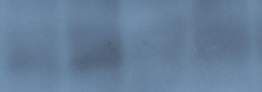

Supplement: Figure 3—source data 1. [file elife-108131-fig3-data1.zip › Figure 3 source data 1 (originals)/Figure 3 source data 1_ E/HIF1A_Fumarate.tif]

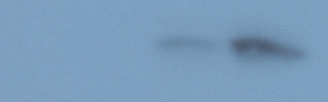

Supplement: Figure 3—source data 1. [file elife-108131-fig3-data1.zip › Figure 3 source data 1 (originals)/Figure 3 source data 1_ E/PhosH3_Fumarate.tif]

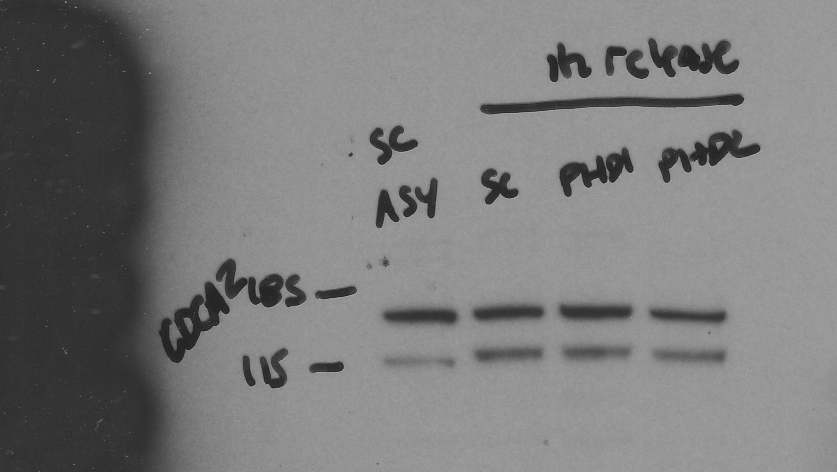

Supplement: Figure 3—source data 1. [file elife-108131-fig3-data1.zip › Figure 3 source data 1 (originals)/Figure 3- source data 1 _ H/Figure 3H_RepoMan.tiff]

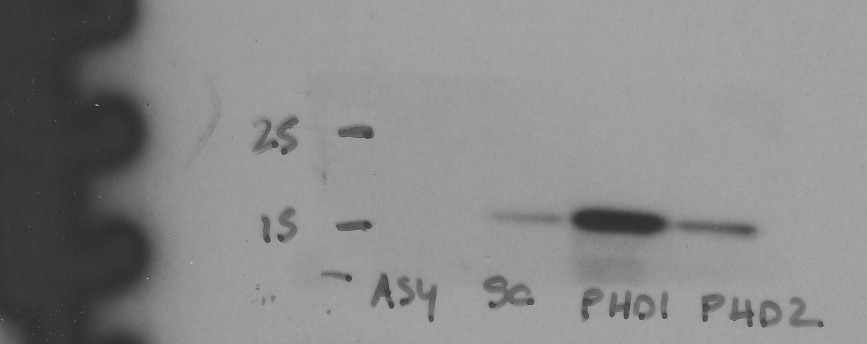

Supplement: Figure 3—source data 1. [file elife-108131-fig3-data1.zip › Figure 3 source data 1 (originals)/Figure 3- source data 1 _ H/Figure_3H_pT3H3.tiff]

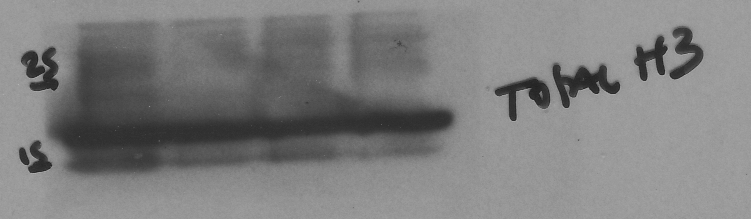

Supplement: Figure 3—source data 1. [file elife-108131-fig3-data1.zip › Figure 3 source data 1 (originals)/Figure 3- source data 1 _ H/Figure3H_ H3.tiff]

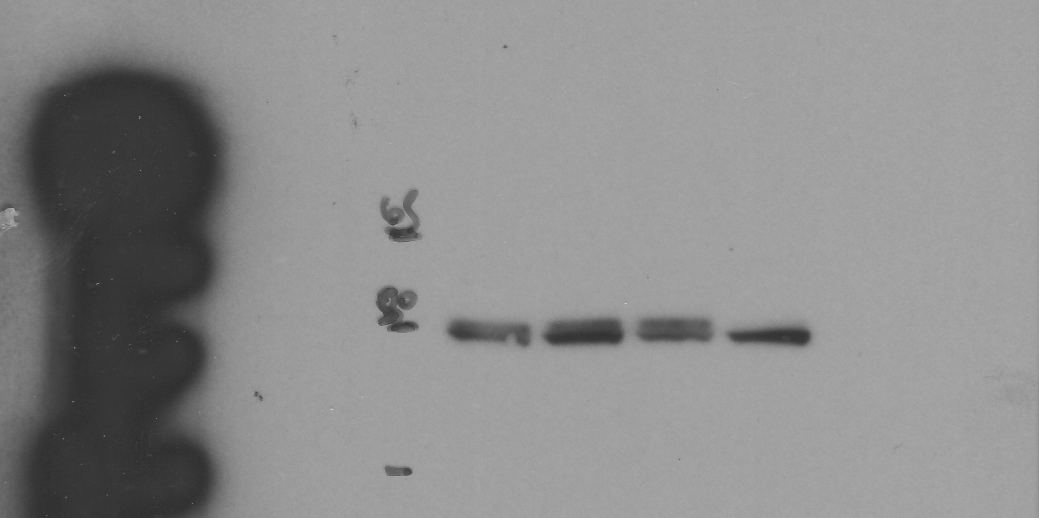

Supplement: Figure 3—source data 1. [file elife-108131-fig3-data1.zip › Figure 3 source data 1 (originals)/Figure 3- source data 1 _ H/Figure3H_PHD2.tiff]

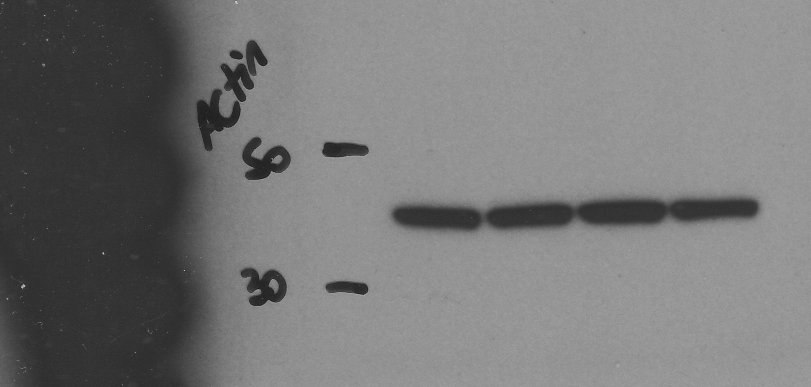

Supplement: Figure 3—source data 1. [file elife-108131-fig3-data1.zip › Figure 3 source data 1 (originals)/Figure 3- source data 1 _ H/Figure3H_actin.tiff]

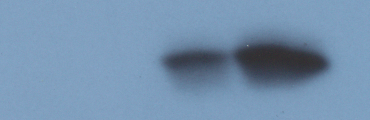

Supplement: Figure 3—source data 1. [file elife-108131-fig3-data1.zip › Figure 3 source data 1 (originals)/Figure 3 source data 1_ D/PhosH3_FG.tif]

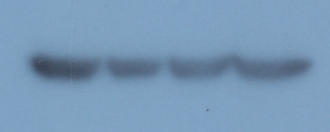

Supplement: Figure 3—source data 1. [file elife-108131-fig3-data1.zip › Figure 3 source data 1 (originals)/Figure 3 source data 1_ D/Figure 3 -source data 1-Actin_FG.tif]

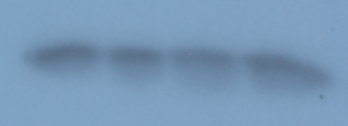

Supplement: Figure 3—source data 1. [file elife-108131-fig3-data1.zip › Figure 3 source data 1 (originals)/Figure 3 source data 1_ D/H3_FG.tif]

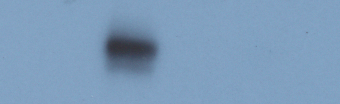

Supplement: Figure 3—source data 1. [file elife-108131-fig3-data1.zip › Figure 3 source data 1 (originals)/Figure 3 source data 1_ D/HIF1A_FG.tif]

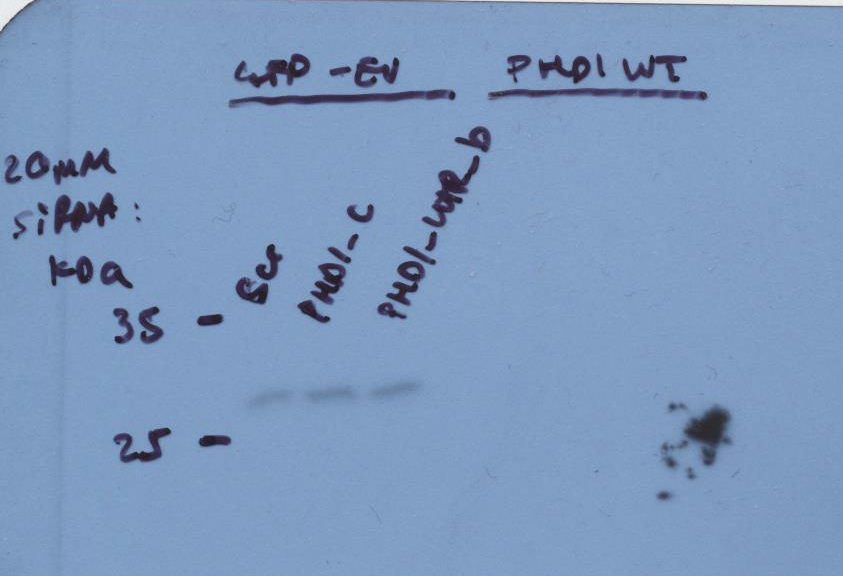

Supplement: Figure 3—figure supplement 1—source data 1. [file elife-108131-fig3-figsupp1-data1.zip › Figure 3_figure supplement 1_source data 1/Figure 3_ figure suplemment 1 _source data 1_GFP EV.tif]

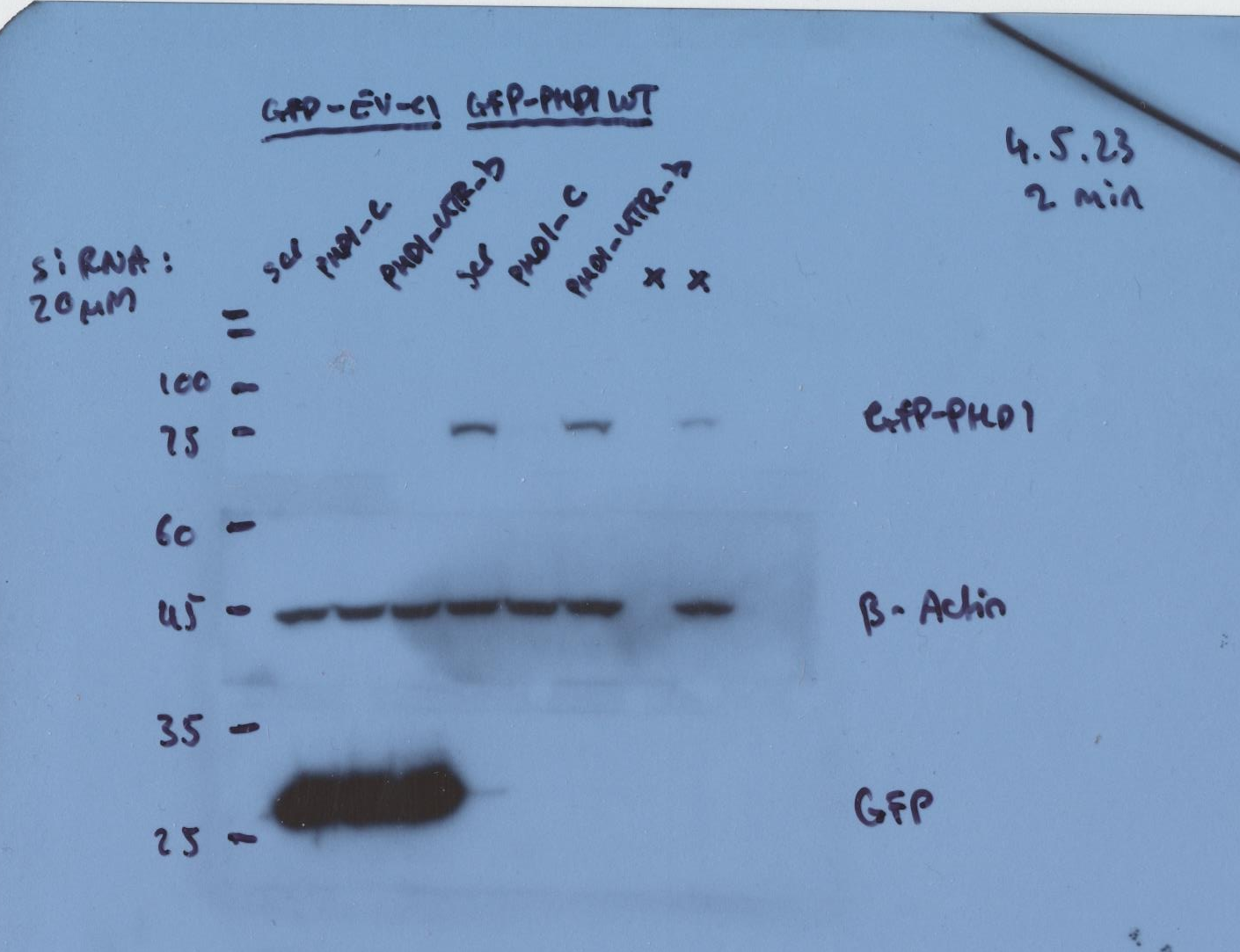

Supplement: Figure 3—figure supplement 1—source data 1. [file elife-108131-fig3-figsupp1-data1.zip › Figure 3_figure supplement 1_source data 1/Figure 3_figure supplement 1_Source data 1.tif]

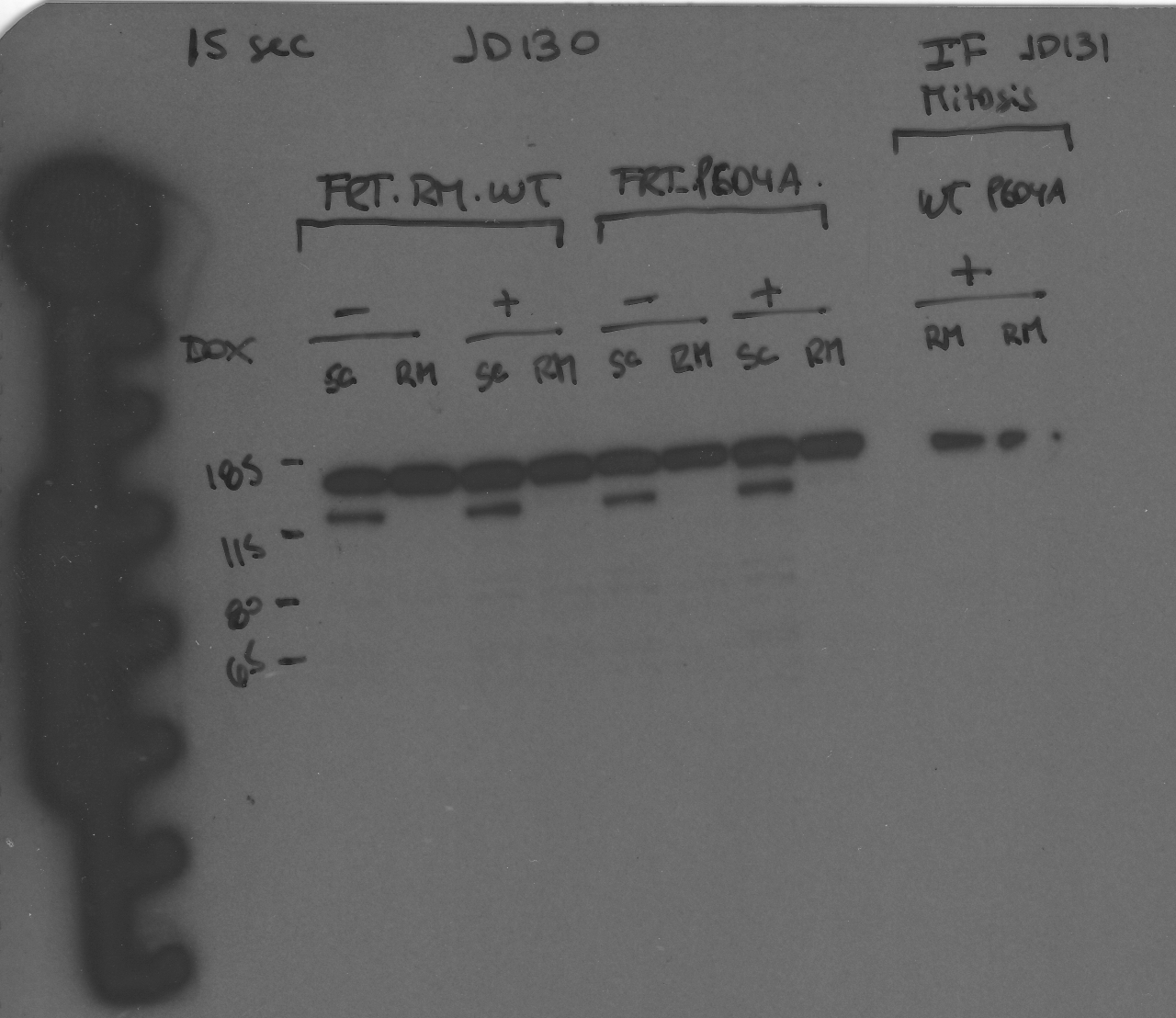

Supplement: Figure 4—source data 1. [file elife-108131-fig4-data1.zip › Figure 4-source data 1 (originals)/Figure 4- source data 1_RepoMan.tiff]

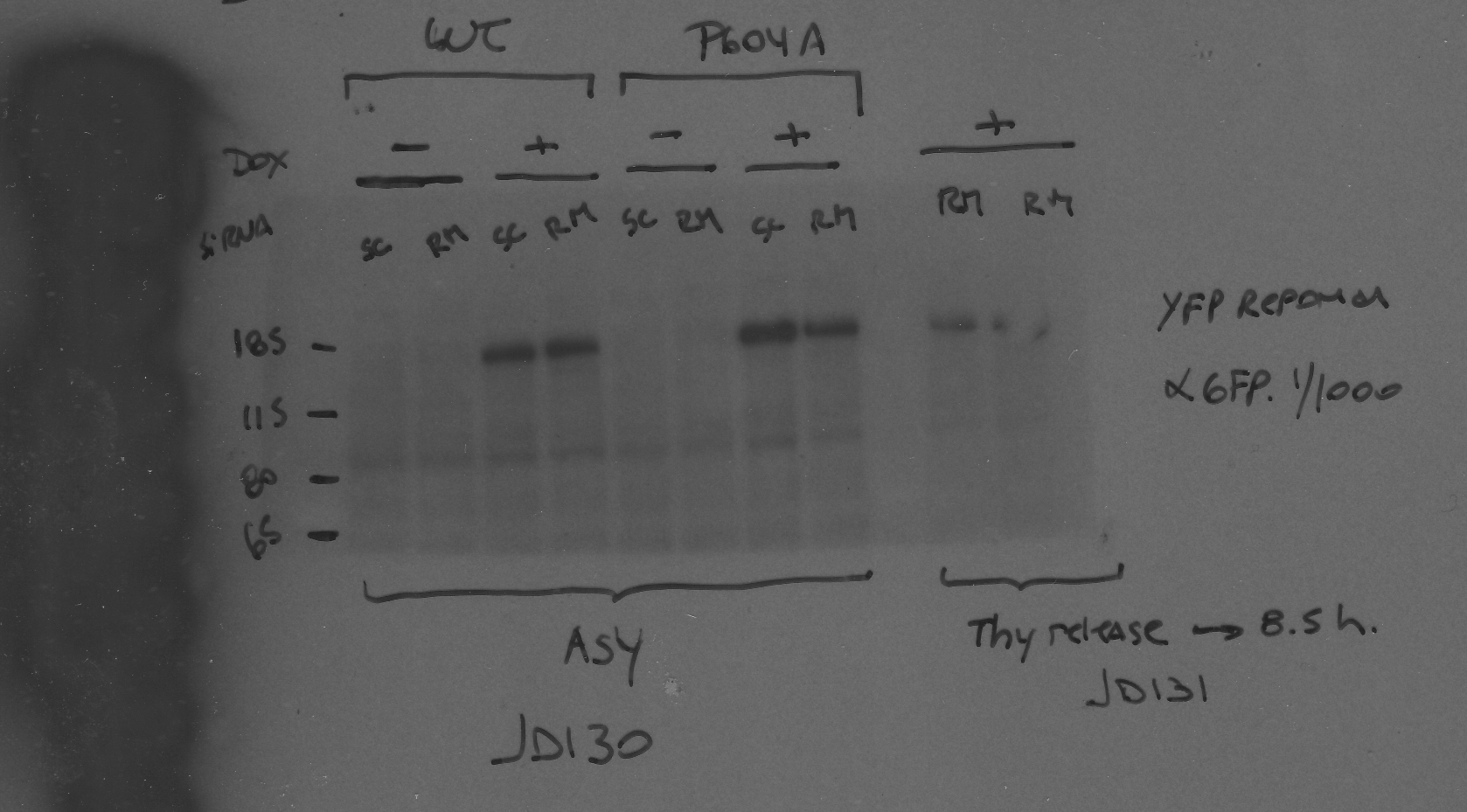

Supplement: Figure 4—source data 1. [file elife-108131-fig4-data1.zip › Figure 4-source data 1 (originals)/Figure 4- source data 1 _GFP.tiff]

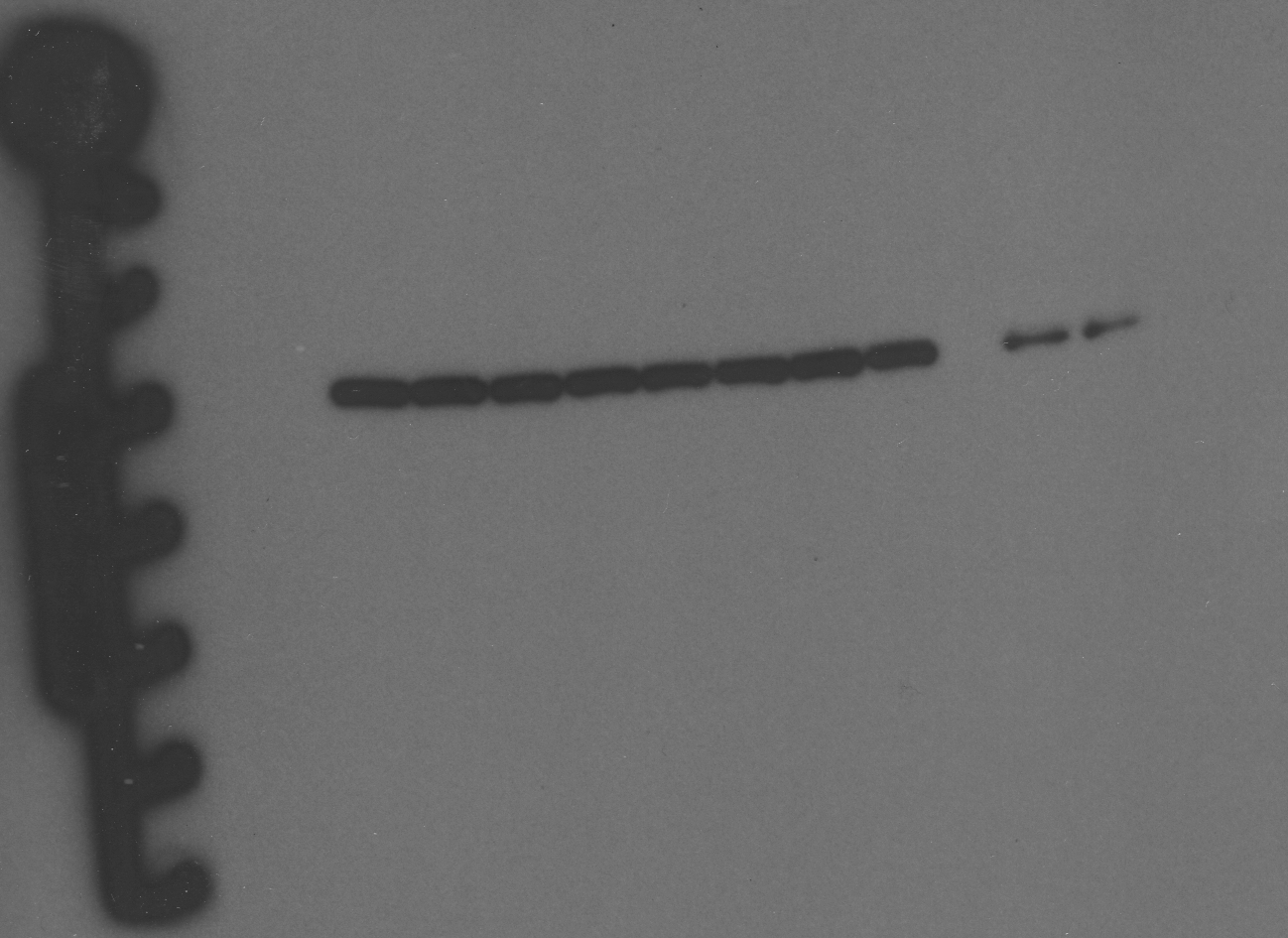

Supplement: Figure 4—source data 1. [file elife-108131-fig4-data1.zip › Figure 4-source data 1 (originals)/Figure 4-source data 1_Actin.tiff]

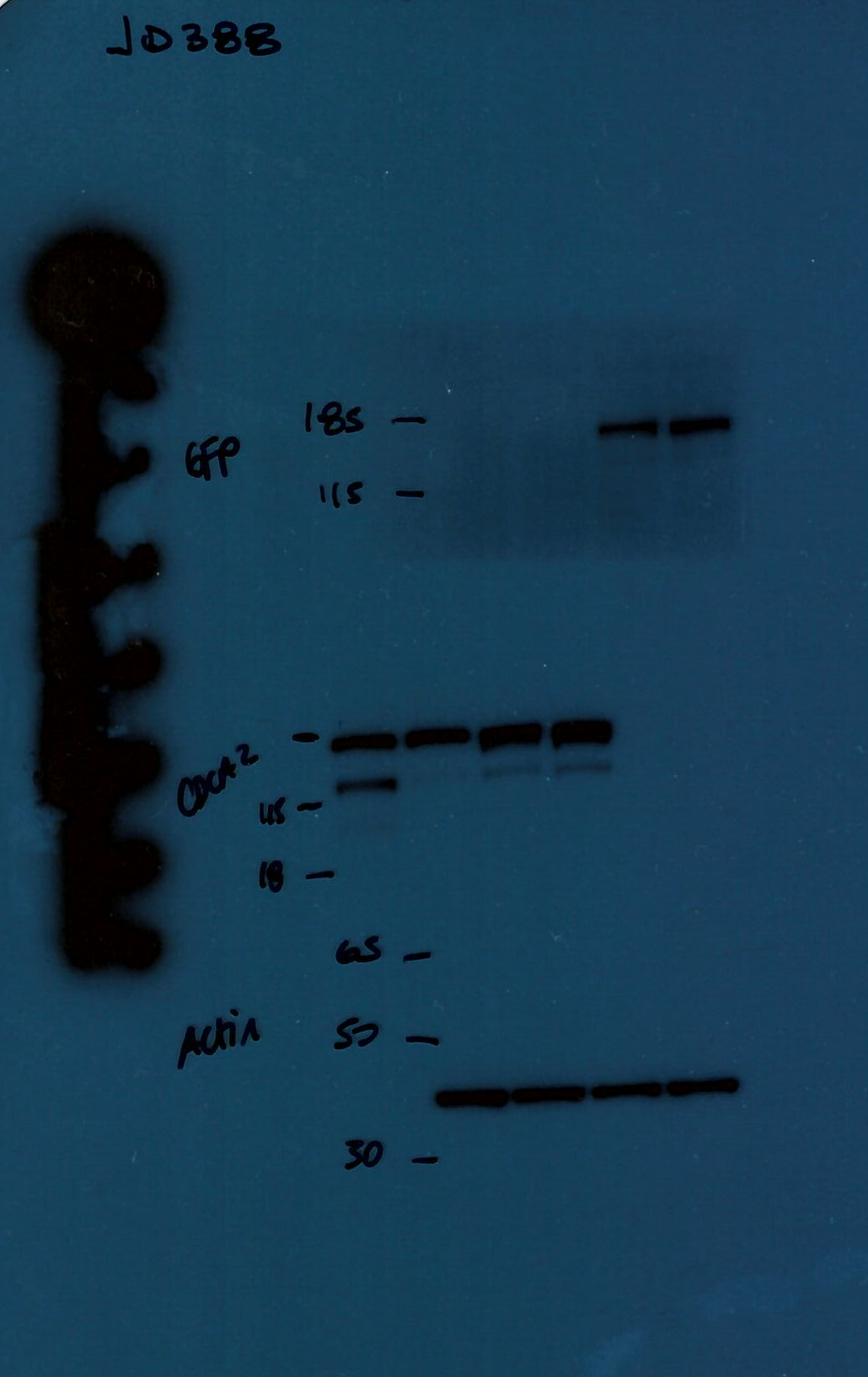

Supplement: Figure 4—figure supplement 1—source data 1. [file elife-108131-fig4-figsupp1-data1.zip › Figure 4-figure supplement 1- source data 1/Figure 4-figure suplemment 1-source data 1.tiff]

Figure 4- figure supplement 1\_source data 2

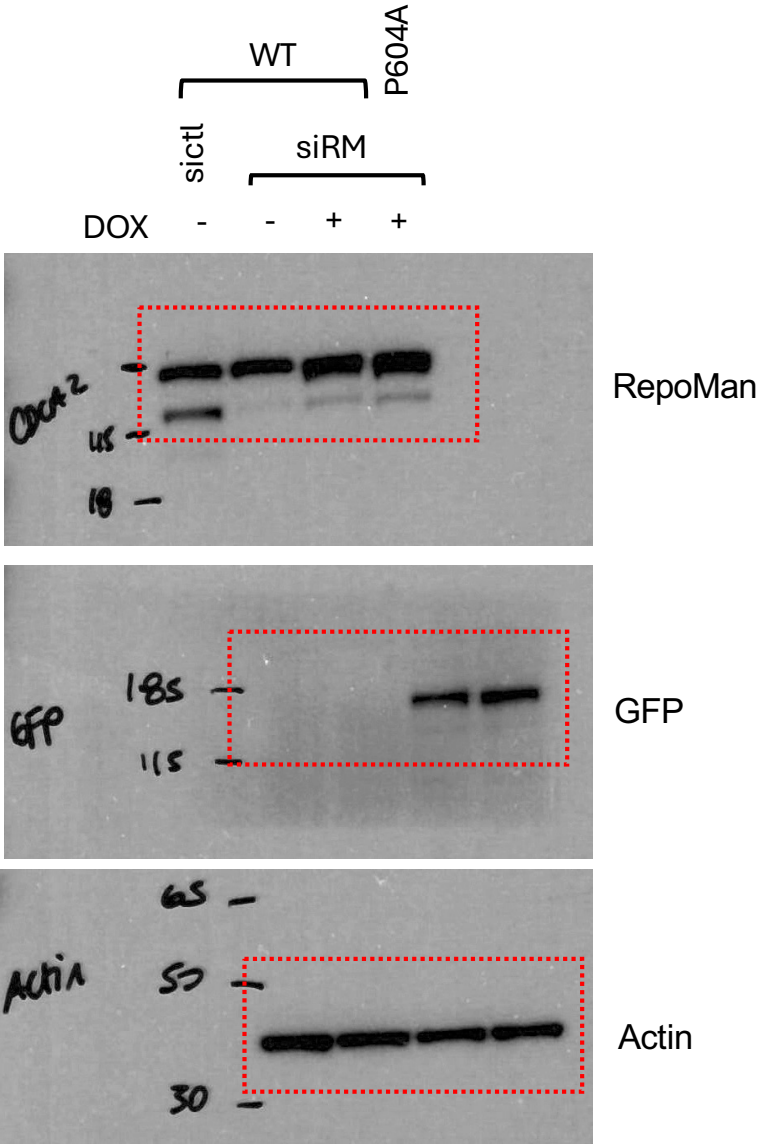

Supplement: Figure 4—figure supplement 1—source data 2. [file elife-108131-fig4-figsupp1-data2.pdf]

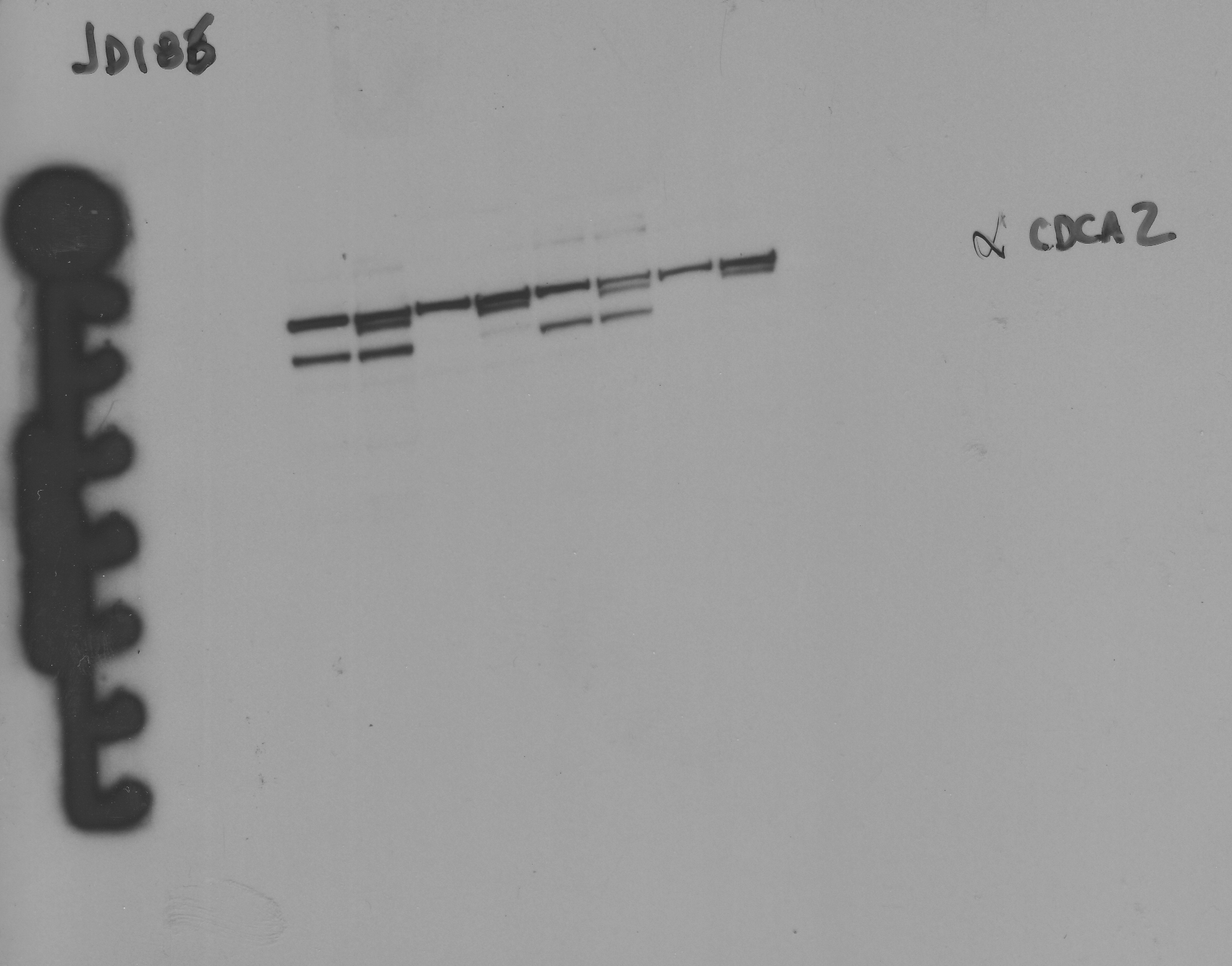

Supplement: Figure 5—source data 1. [file elife-108131-fig5-data1.zip › Figure 5- source data 1 (originals)/Figure 5- source data 1- E/Fig5E_Repoman.tiff]

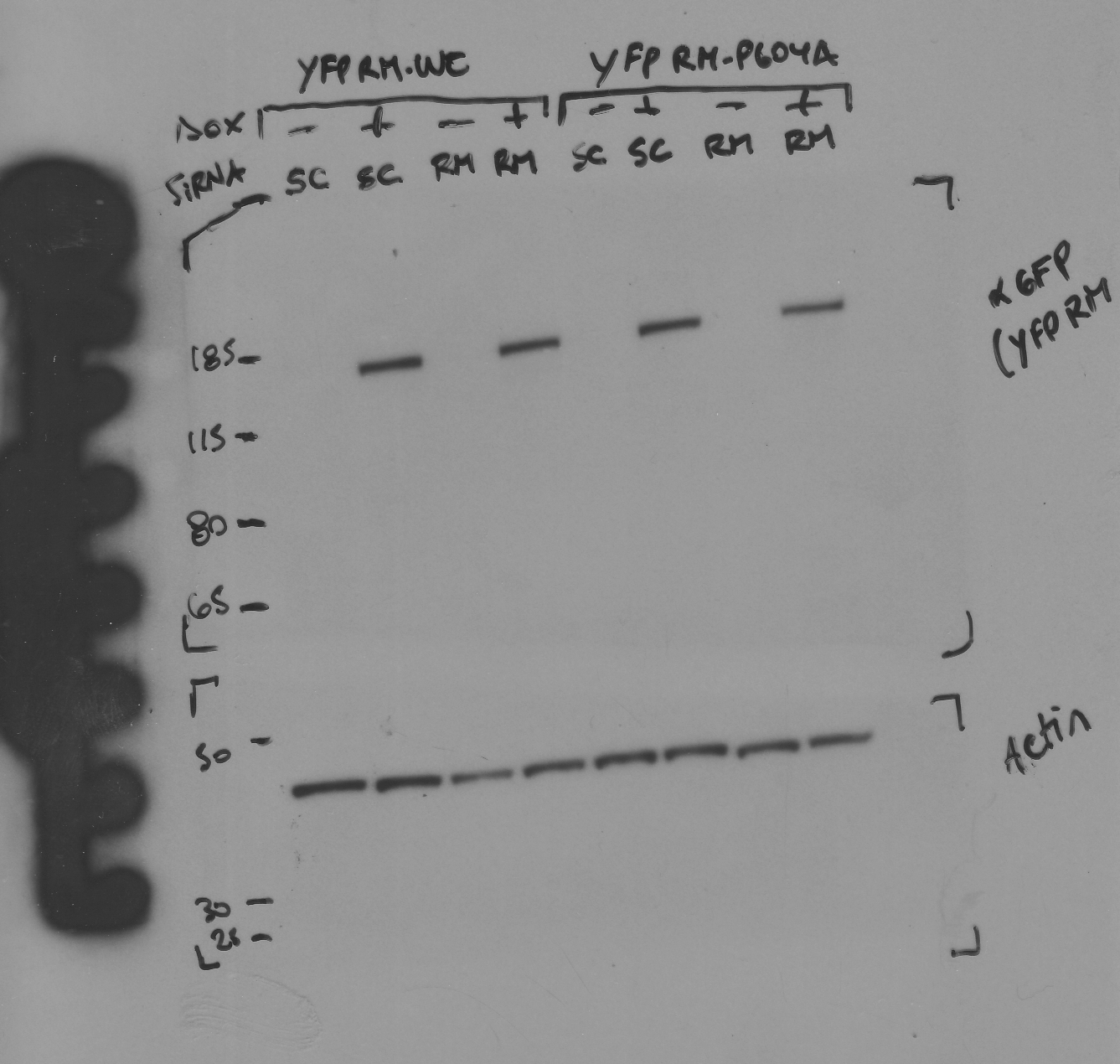

Supplement: Figure 5—source data 1. [file elife-108131-fig5-data1.zip › Figure 5- source data 1 (originals)/Figure 5- source data 1- E/Fig5D_Actin_GFP.tiff]

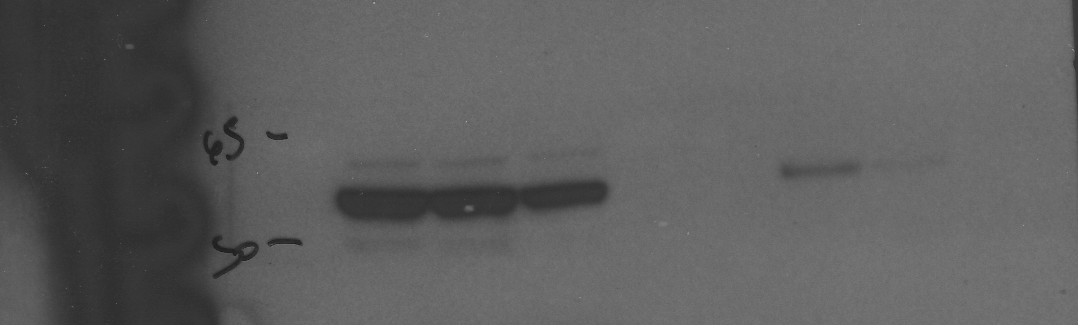

Supplement: Figure 5—source data 1. [file elife-108131-fig5-data1.zip › Figure 5- source data 1 (originals)/Figure 5- source data 1 - C/Figure5_C _B56.tiff]

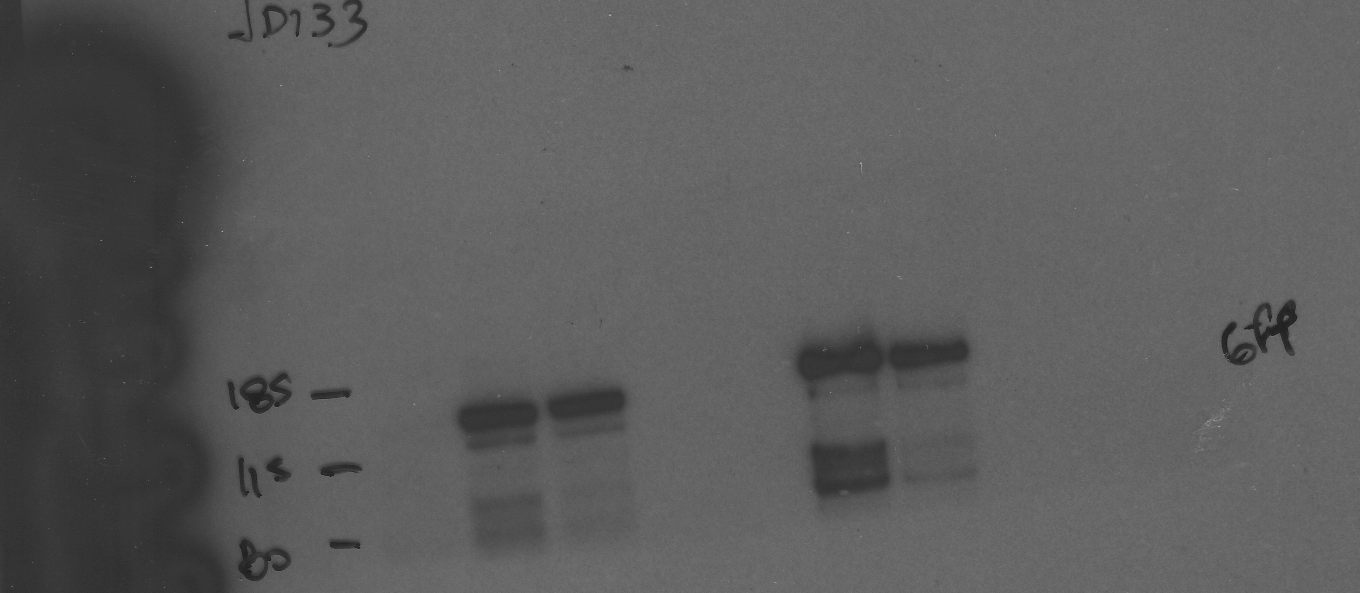

Supplement: Figure 5—source data 1. [file elife-108131-fig5-data1.zip › Figure 5- source data 1 (originals)/Figure 5- source data 1 - C/Figure_5_GFP Ip.tiff]

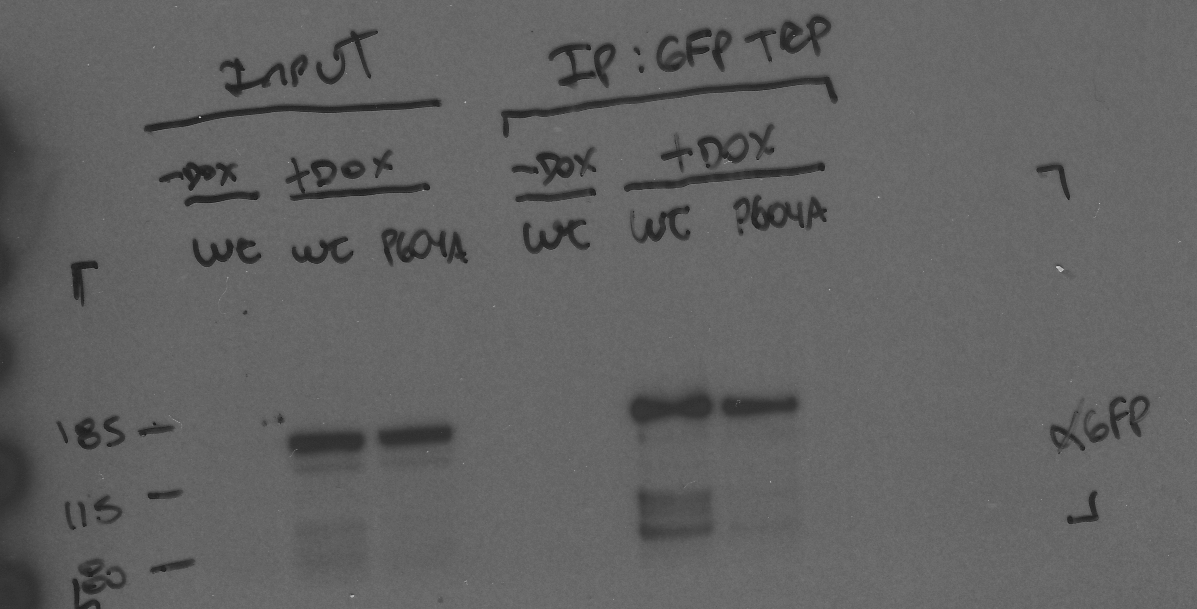

Supplement: Figure 5—source data 1. [file elife-108131-fig5-data1.zip › Figure 5- source data 1 (originals)/Figure 5- source data 1 - C/Figure 5_C GFP input.tiff]

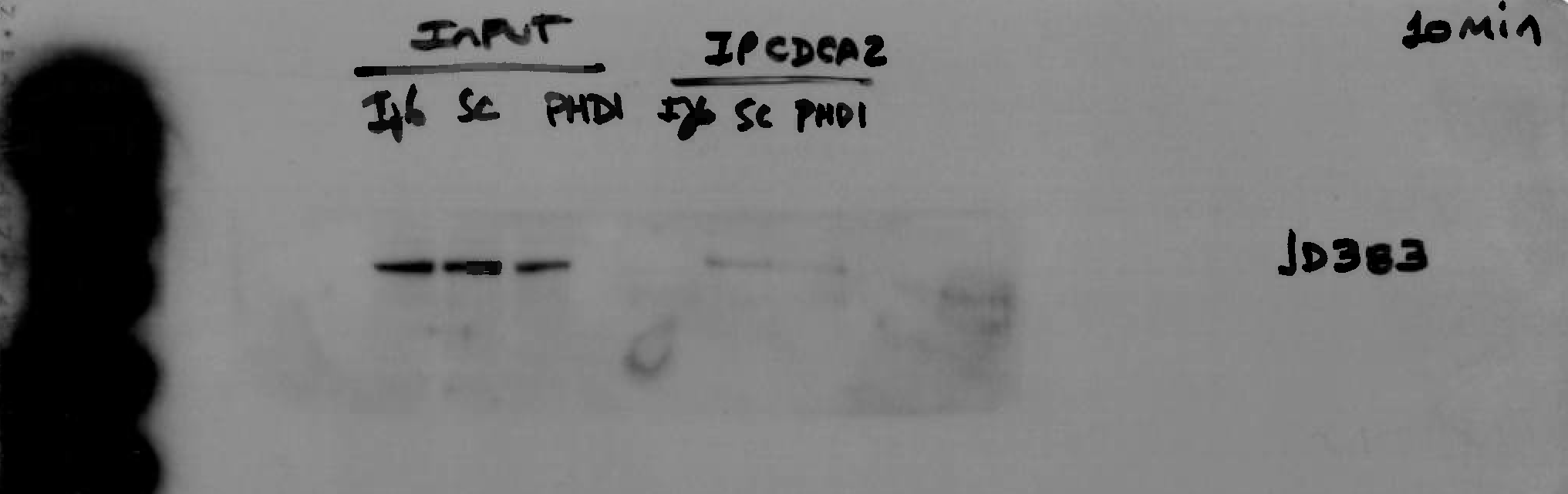

Supplement: Figure 5—source data 1. [file elife-108131-fig5-data1.zip › Figure 5- source data 1 (originals)/Figure 5 - source data 1- D/Figure 5D_B56.tiff]

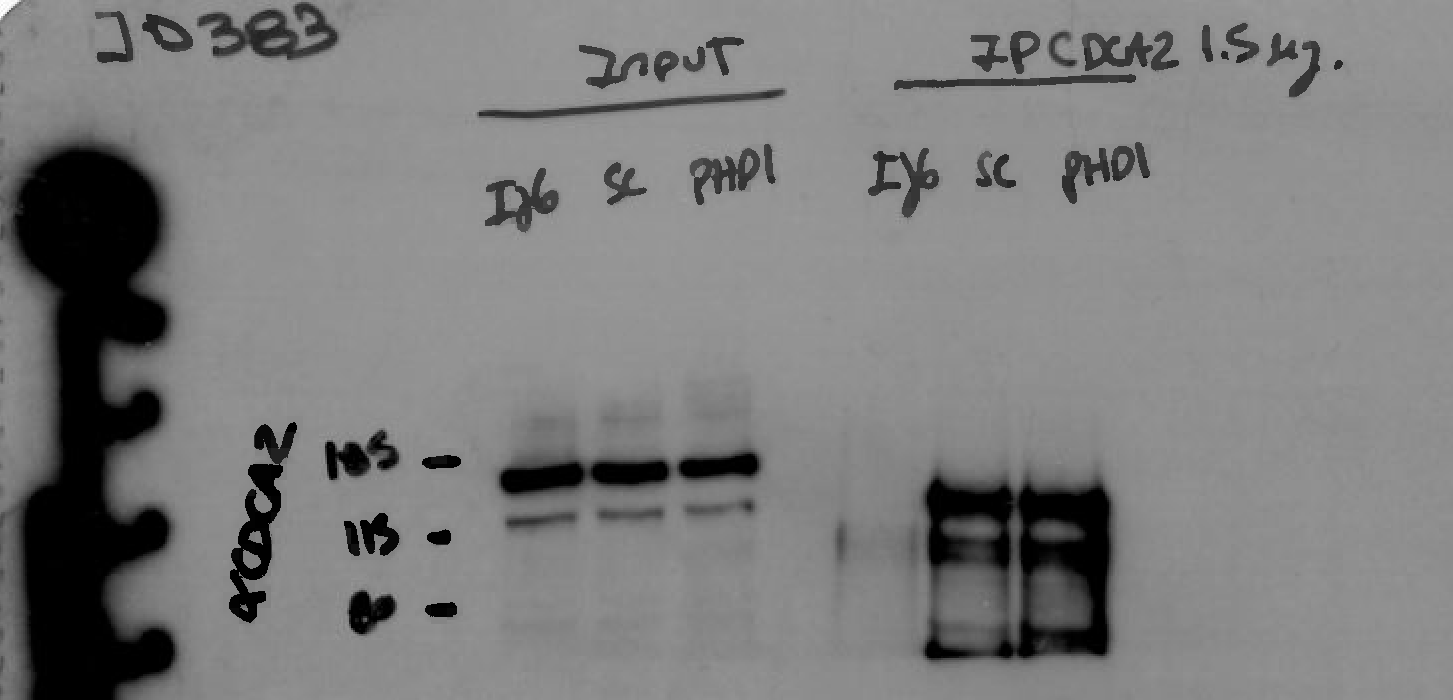

Supplement: Figure 5—source data 1. [file elife-108131-fig5-data1.zip › Figure 5- source data 1 (originals)/Figure 5 - source data 1- D/Fig5D_Repoman.tiff]

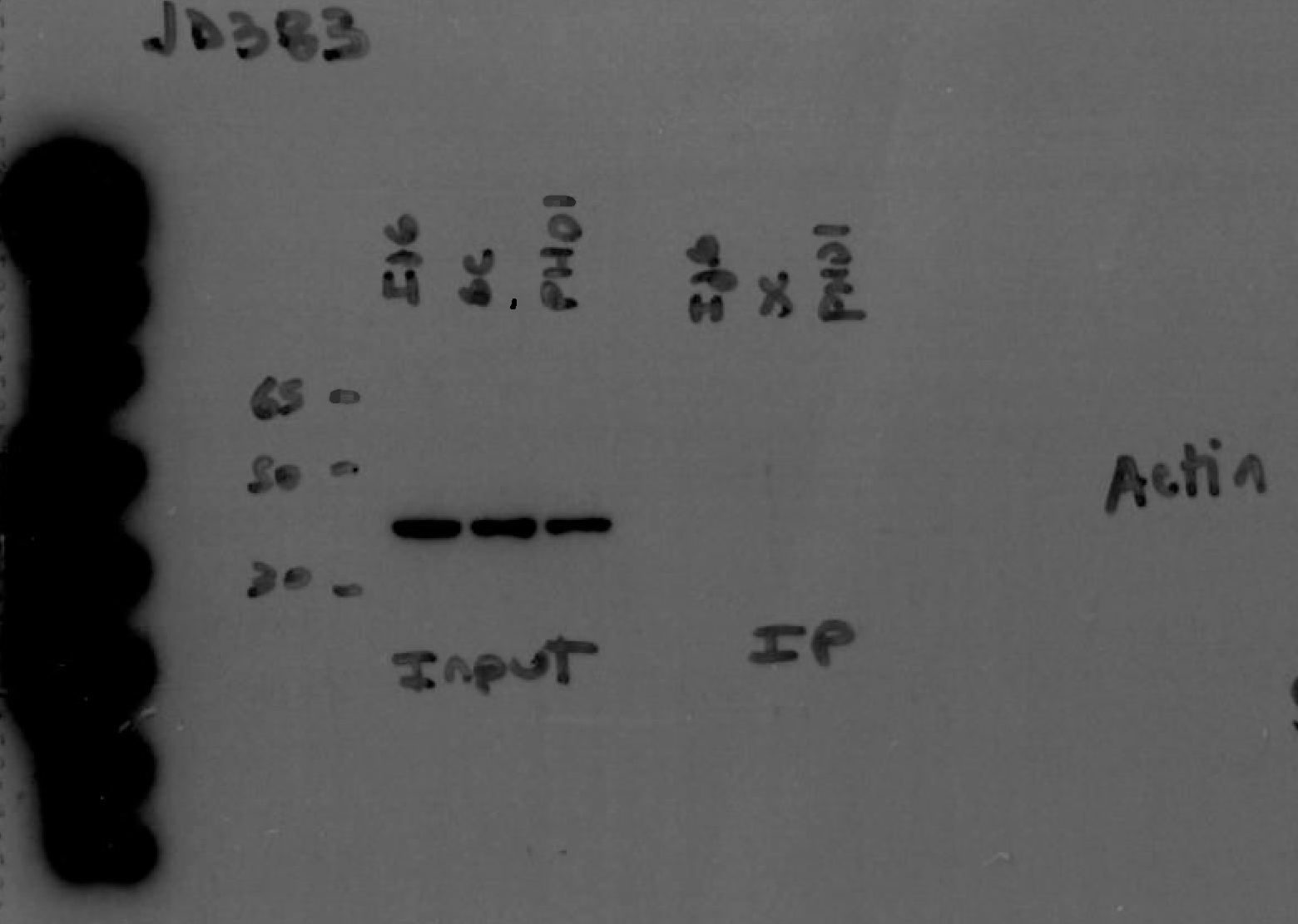

Supplement: Figure 5—source data 1. [file elife-108131-fig5-data1.zip › Figure 5- source data 1 (originals)/Figure 5 - source data 1- D/Fig5D_Actin.tiff]

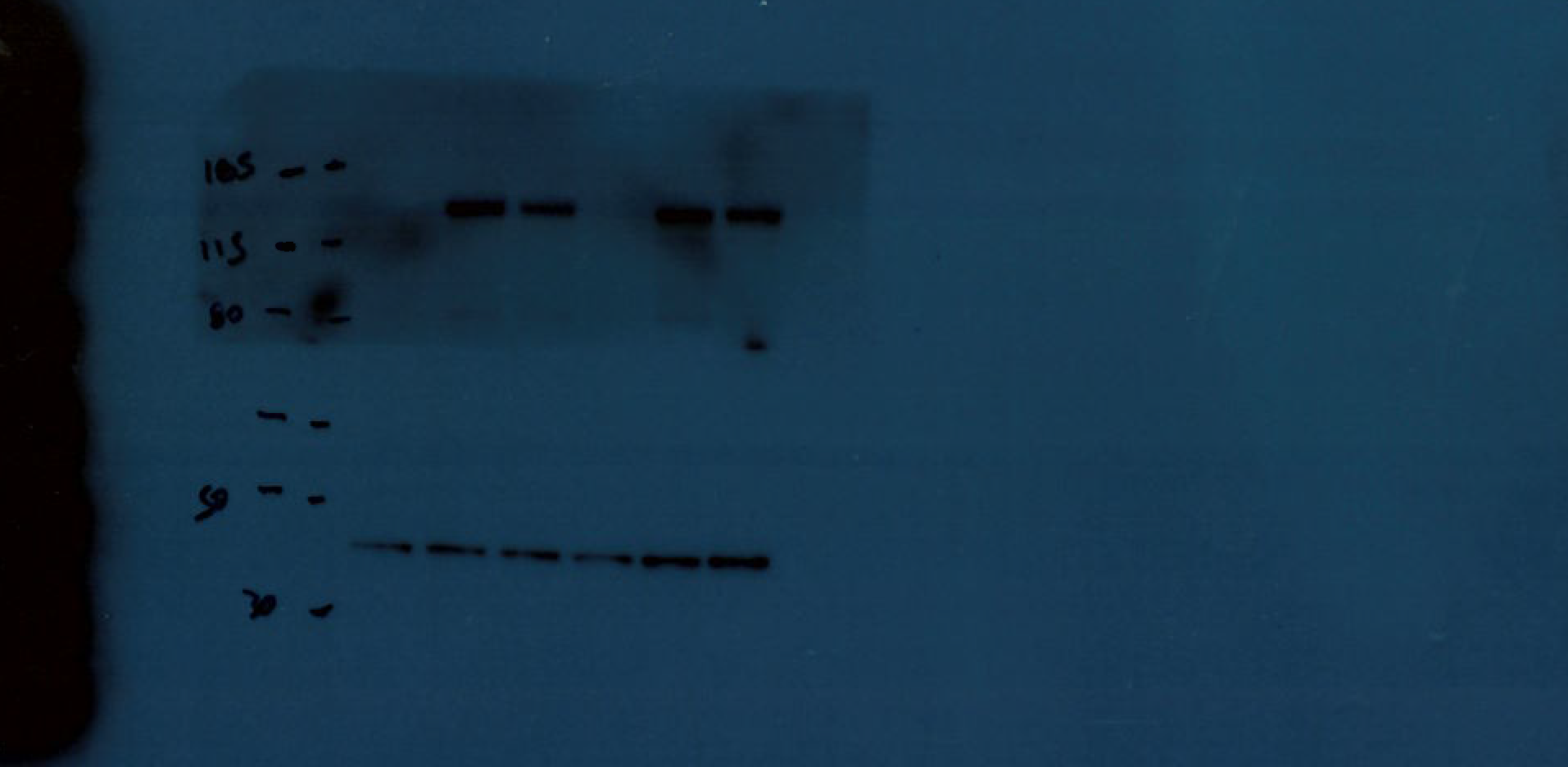

Supplement: Figure 5—figure supplement 1—source data 1. [file elife-108131-fig5-figsupp1-data1.zip › Figure 5_ figure supplement 1_source data 1/Figure 5-figure supplement 1_B GPF_Actin .tif]

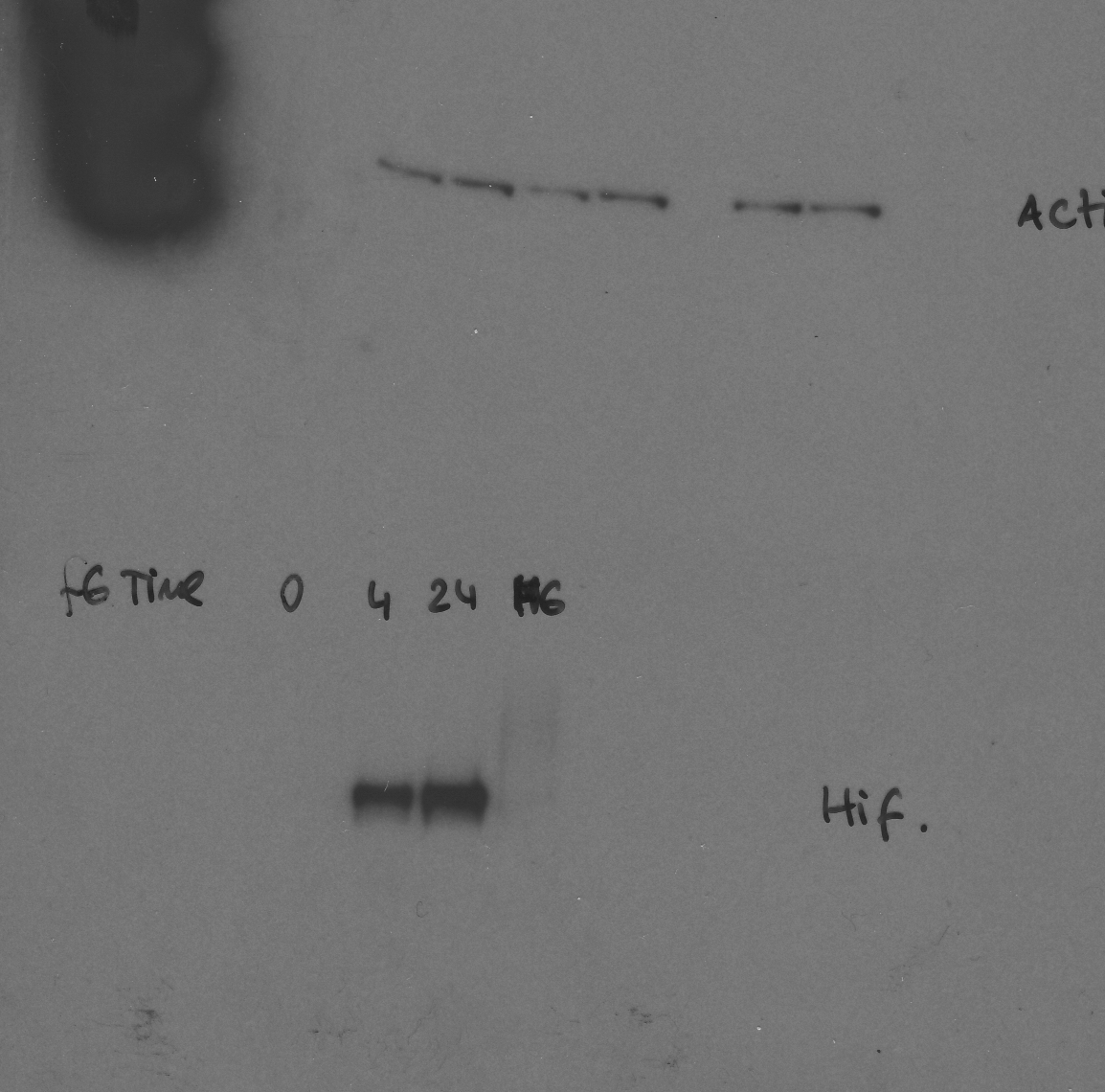

Supplement: Figure 5—figure supplement 1—source data 1. [file elife-108131-fig5-figsupp1-data1.zip › Figure 5_ figure supplement 1_source data 1/Figure 5_ Figure supplement 1 A_ HIF and Actin.tif]

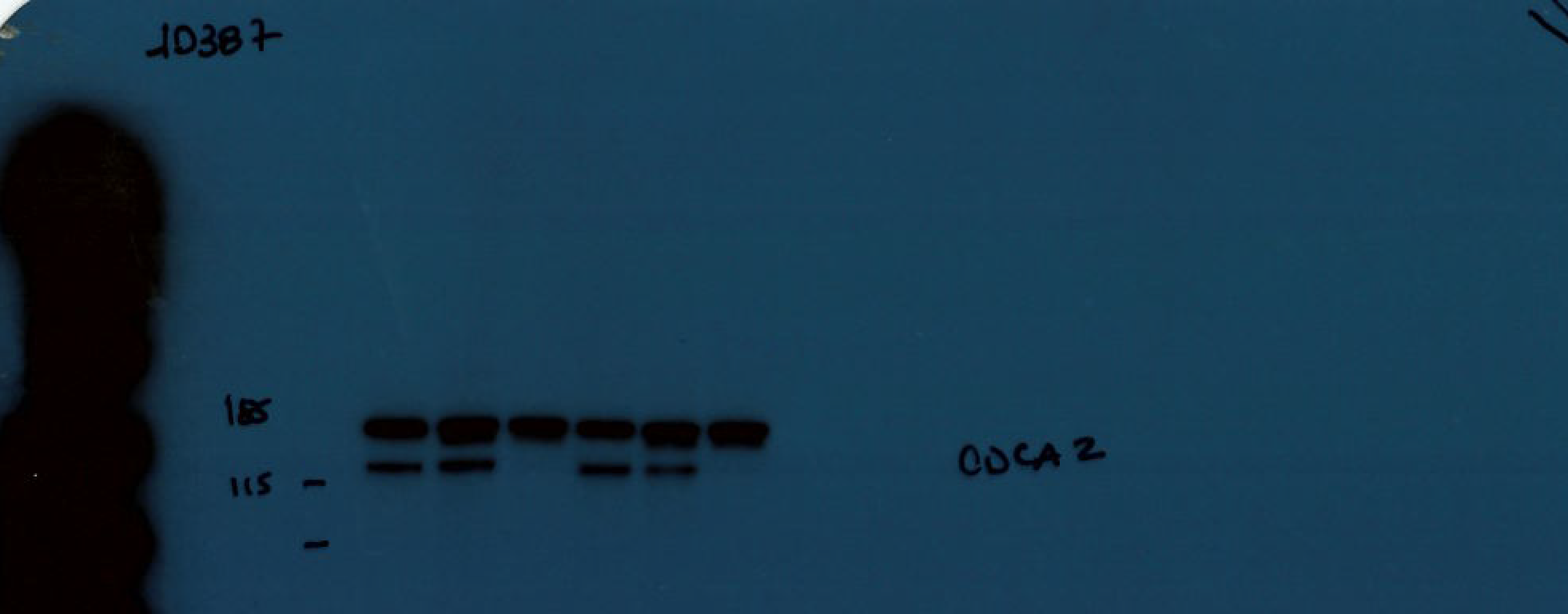

Supplement: Figure 5—figure supplement 1—source data 1. [file elife-108131-fig5-figsupp1-data1.zip › Figure 5_ figure supplement 1_source data 1/Figure 5- figure supplement 1_B Repoman.tif]

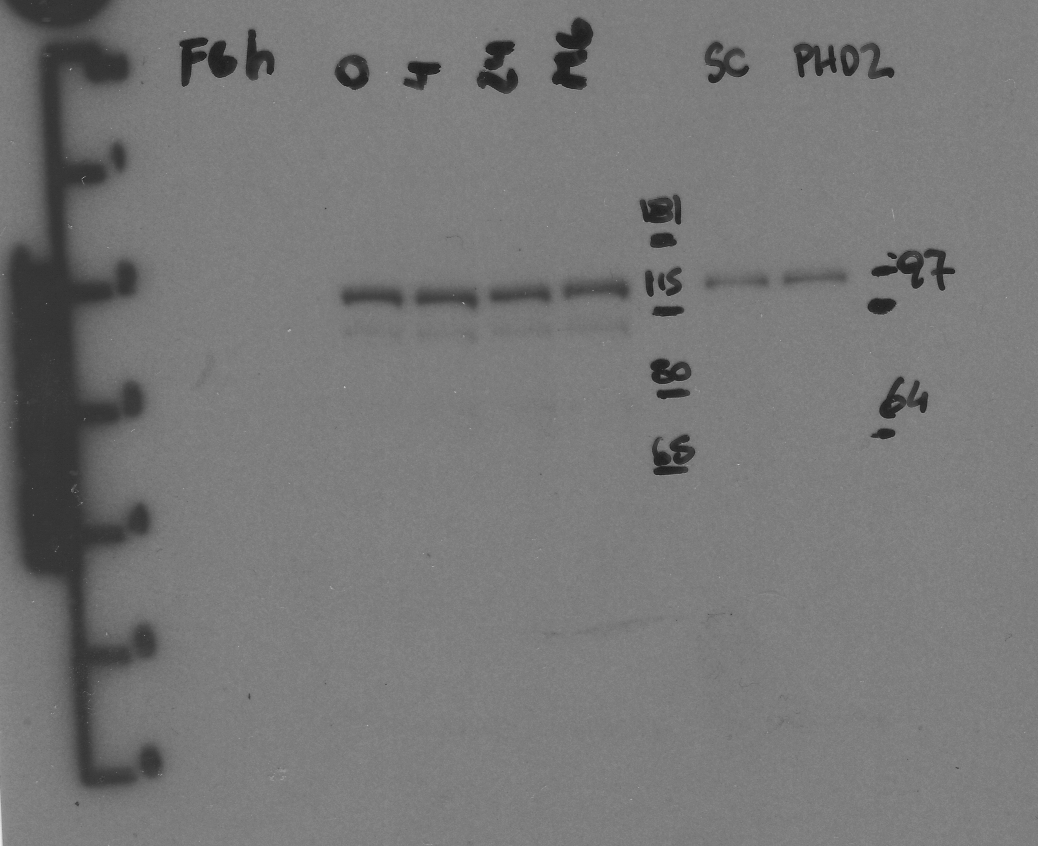

Supplement: Figure 5—figure supplement 1—source data 1. [file elife-108131-fig5-figsupp1-data1.zip › Figure 5_ figure supplement 1_source data 1/Figure 5_ figure supplement 1-A_Repoman .tif]

Figure 5- figure supplement 1\_source data 2

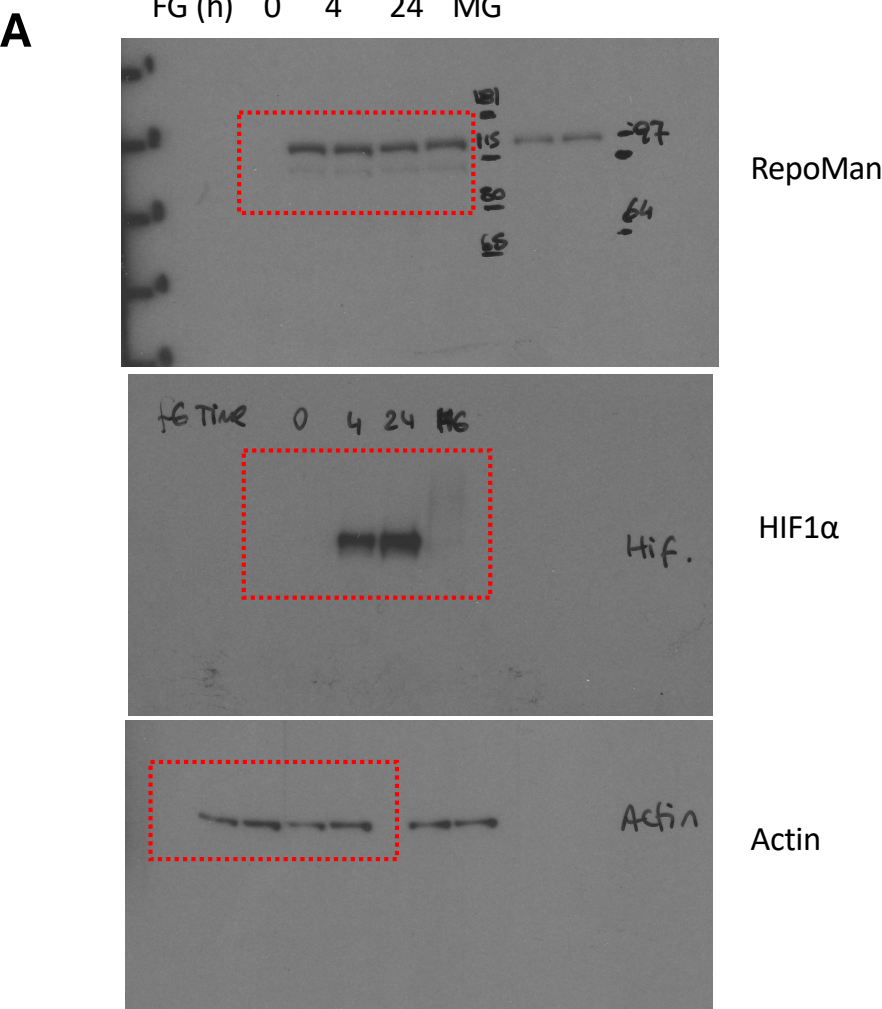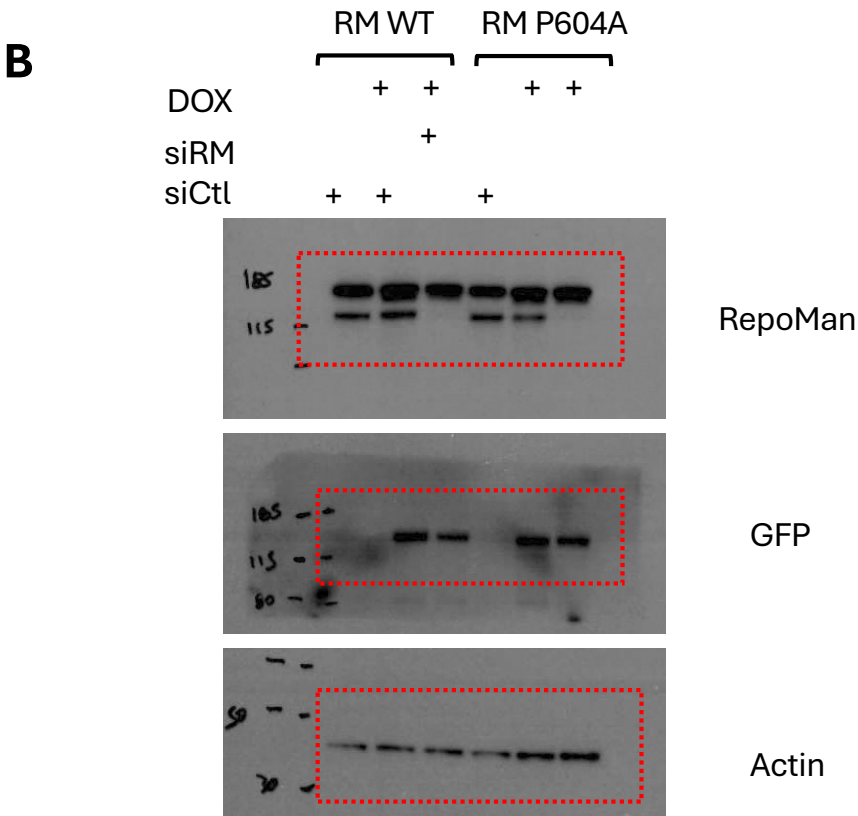

Supplement: Figure 5—figure supplement 1—source data 2. [file elife-108131-fig5-figsupp1-data2.pdf]

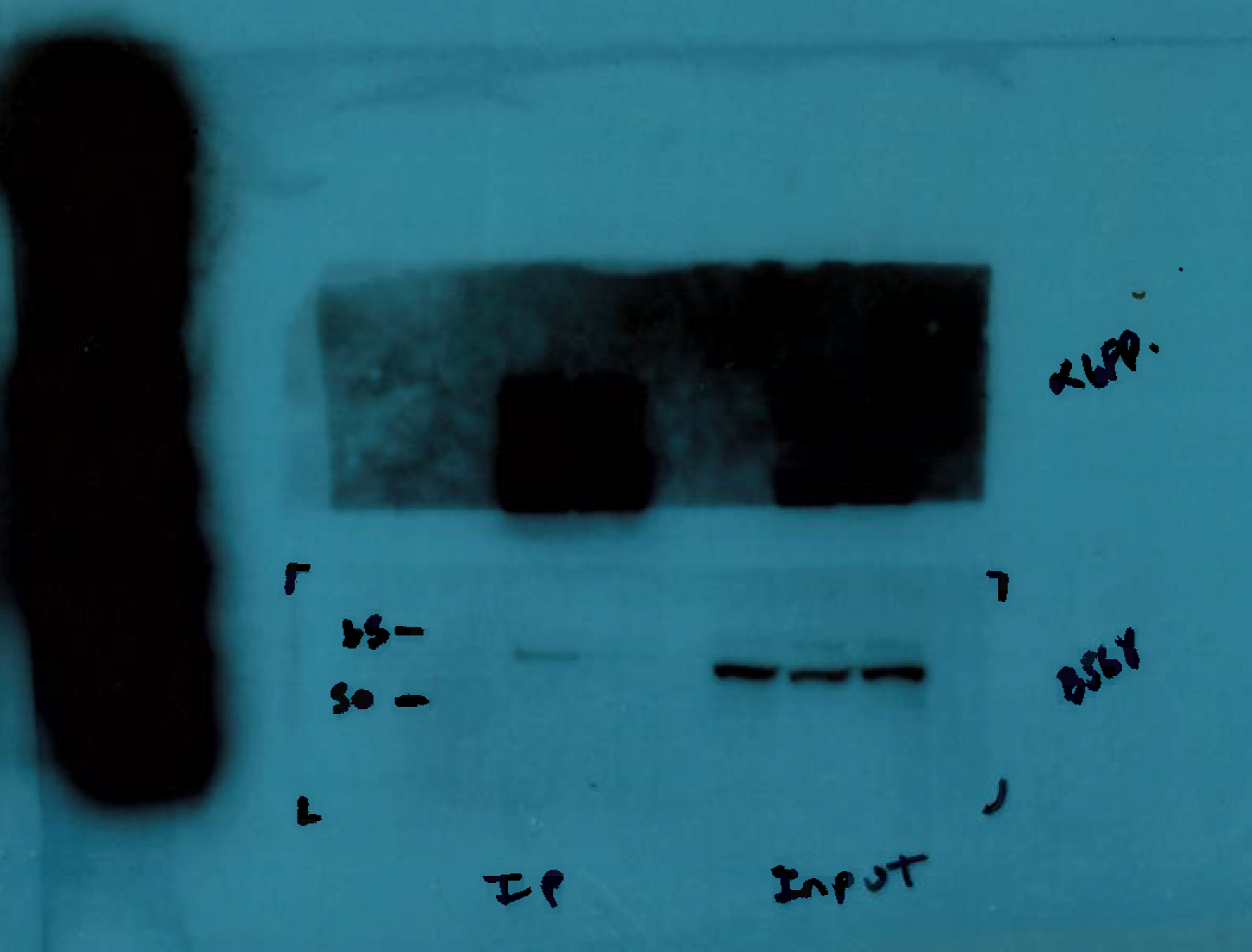

Supplement: Figure 5—figure supplement 3—source data 1. [file elife-108131-fig5-figsupp3-data1.zip › Figure 5-figure supplement 3 -source data 1/Figure 5_supplement 3. B56g .tif]

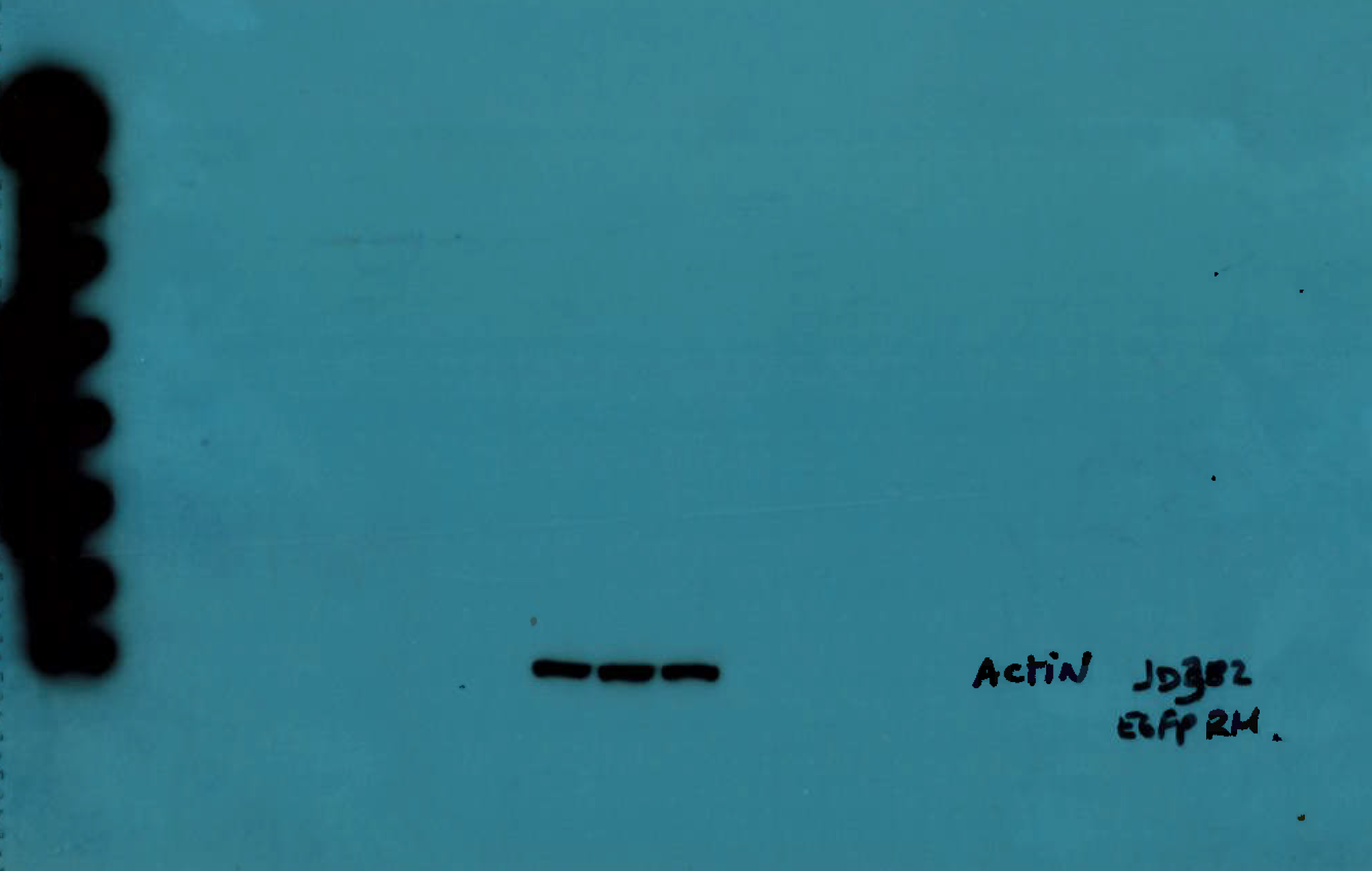

Supplement: Figure 5—figure supplement 3—source data 1. [file elife-108131-fig5-figsupp3-data1.zip › Figure 5-figure supplement 3 -source data 1/Figure 5 - figure supplement 3. actin .tif]

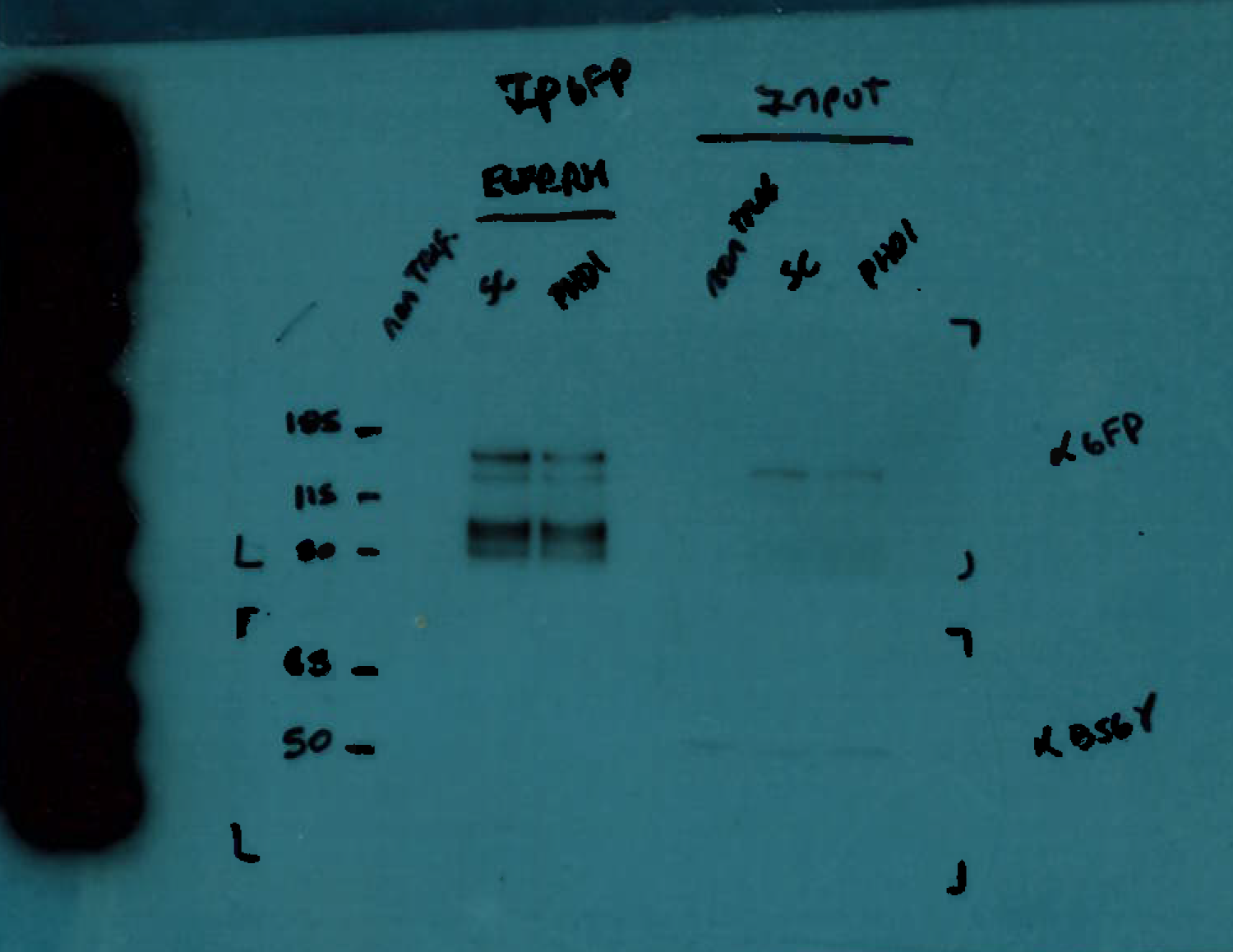

Supplement: Figure 5—figure supplement 3—source data 1. [file elife-108131-fig5-figsupp3-data1.zip › Figure 5-figure supplement 3 -source data 1/Figure 5_supplement 3. GFP. tif.tiff]

Figure 5- figure supplement 3\_source data 2

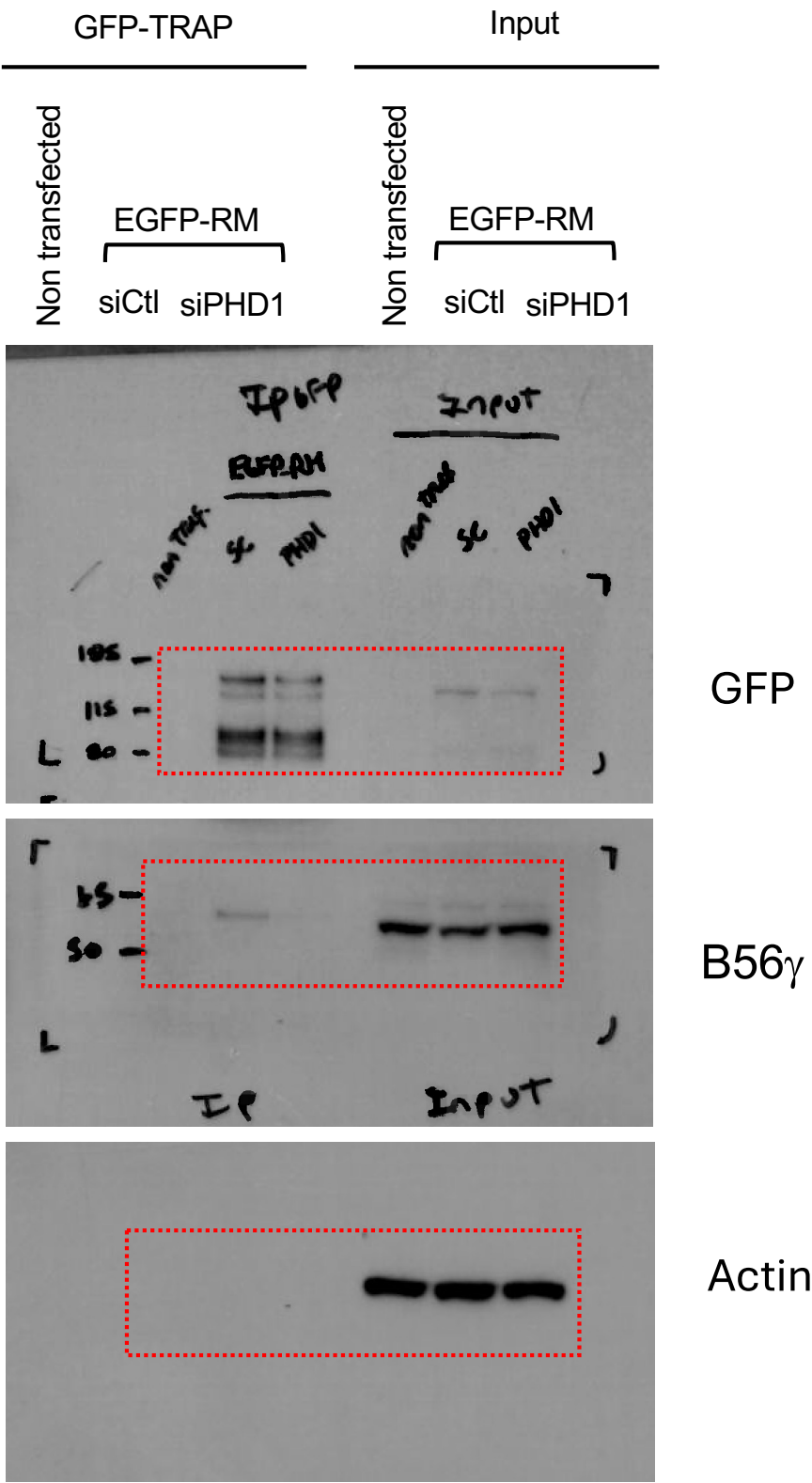

Supplement: Figure 5—figure supplement 3—source data 2. [file elife-108131-fig5-figsupp3-data2.pdf]

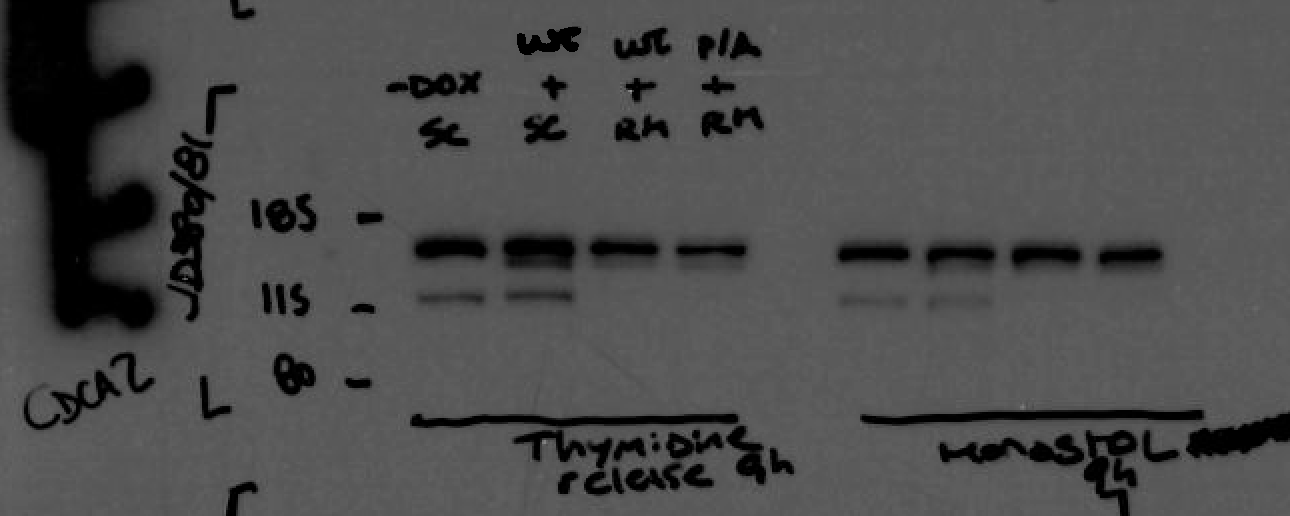

Supplement: Figure 6—figure supplement 1—source data 1. [file elife-108131-fig6-figsupp1-data1.zip › Figure 6-figure supplement 1-source data 1/Figure 6_ figure supplement 1. Repoman.tif]

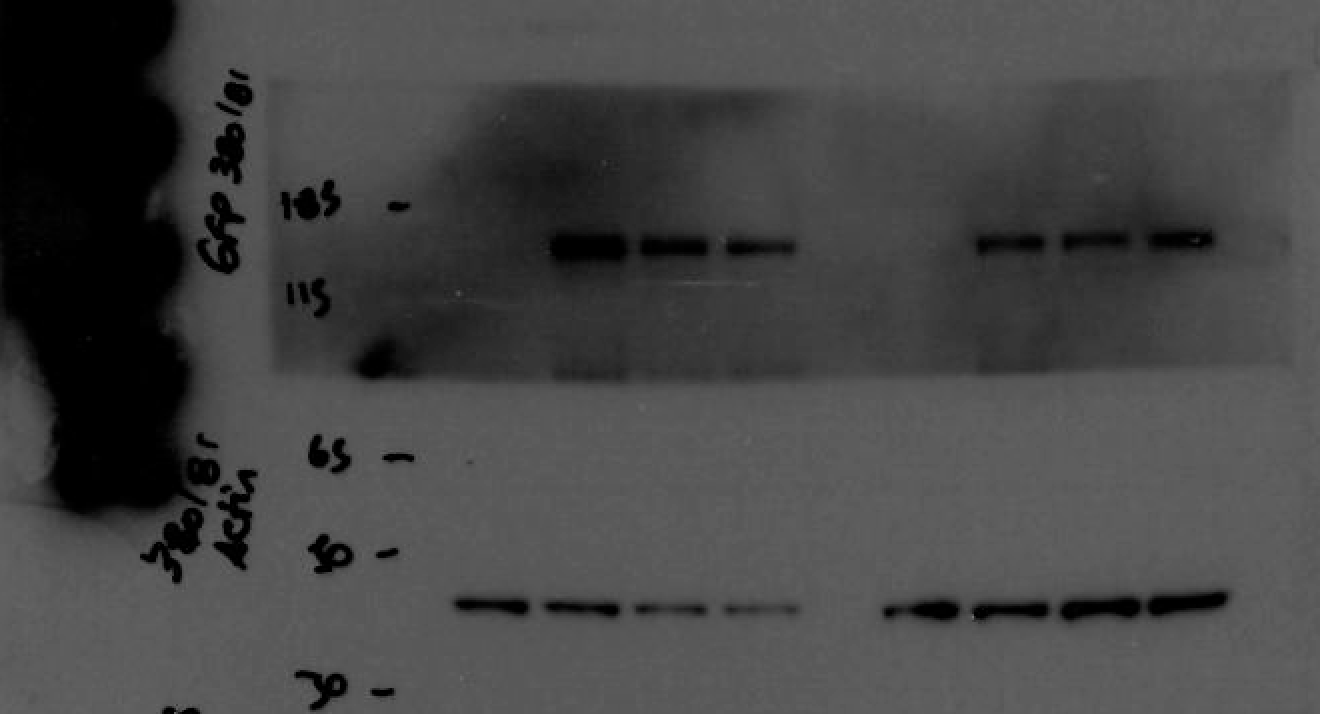

Supplement: Figure 6—figure supplement 1—source data 1. [file elife-108131-fig6-figsupp1-data1.zip › Figure 6-figure supplement 1-source data 1/Figure 6_figure supplement 1. GFP-actin.tif]

Figure 6- figure supplement 1\_source data 2

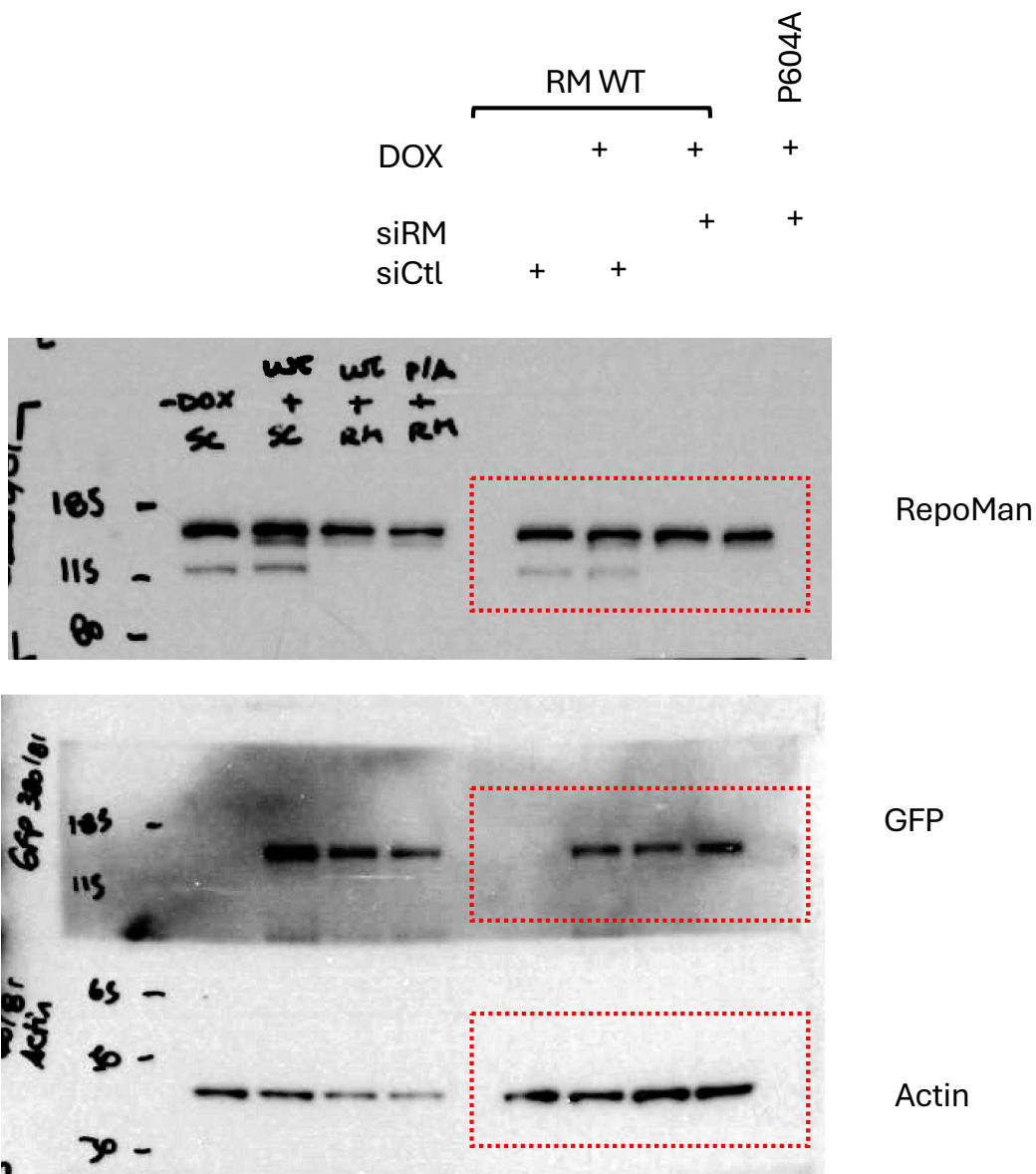

Supplement: Figure 6—figure supplement 1—source data 2. [file elife-108131-fig6-figsupp1-data2.pdf]
