# Supplementary material for: PHD1-dependent hydroxylation of RepoMan (CDCA2) on P604 modulates the control of mitotic progression
Source: eLife. 2026 Jun 25;14:RP108131. doi: 10.7554/eLife.108131 (PMC13299607; doi:10.7554/eLife.108131)
Supplement: Figure 2—source data 3. [file elife-108131-fig2-data3.pdf]

Figure 2- source data 3

Pearson's Correlation Coefficient (PCC)

Interphase	(Pro) metaphase	Anaphase
0.821	0.864	0.207
0.721	0.738	0.27
0.715	0.847	0.087
0.476	0.909	0.135
0.793	0.776	0.137
1	0.909	0.078
0.578	0.722	0
	0.611	0.556
	0.83	0.009
	0.788	
	0.743	
	0.496	
	0.826	
	0.72	
	0.702	
