# Supplementary material for: PHD1-dependent hydroxylation of RepoMan (CDCA2) on P604 modulates the control of mitotic progression
Source: eLife. 2026 Jun 25;14:RP108131. doi: 10.7554/eLife.108131 (PMC13299607; doi:10.7554/eLife.108131)
Supplement: Figure 3—source data 3. [file elife-108131-fig3-data3.pdf]

Figure 3 -source data 3

Control siRNA	PHD1 siRNA	PHD2 siRNA
0.484775606	5.414823312	2.191418595
0.627699873	5.220218114	1.737001685
1.477834665	4.784583738	1.279431485
1.032613711	5.172521509	0.573500867
0.892820656	5.401095487	2.572239929
0.82384264	6.298551235	0.485832133
1.392087913	6.958830424	0.245070539
0.896328461	4.502070638	0.892832084
0.802221827	3.549731334	1.221345789
0.834924403	5.918092608	1.126192263
1.285420737	5.279316947	1.231957669
1.449429507	1.597256	1.742494
1.330069	3.308079	1.299057
0.356578	3.376616	1.597731
1.016322	2.427134	1.019172
0.628863	2.520175	0.464143
0.66785	2.315117	1.200403
1.538846	1.725679	0.838995
0.946558	3.624095	0.893349
1.420363	4.171761	0.655151
1.088058	2.859882	1.038676
0.987467	3.510389	1.393788
1.019026	2.387768	0.95770818
1.37078881	3.1974587	1.4611975
1.18334649	1.07967672	1.08333556
0.97770789	1.50341284	0.5853388
1.07270037	1.25769957	0.69110247
1.16386482	1.54303291	0.8355224
0.79765073	1.29854984	0.65623022
1.20954357	1.44938391	1.08142183
0.64793802	0.82603767	1.05013288
0.86029257	1.38487252	0.86278759
1.17143015	1.02204817	0.74069552
0.74019104	1.20068155	1.22106957
0.88081199	1.92292099	0.78243329
1.0445845	0.99149437	0.67384386
0.87914906	1.80920151	0.722973
0.893548	1.47692808	0.953061
0.645047	1.766206	1.072786

1.030506	1.24839	0.663124
1.02857	1.666374	0.751257
1.121076	1.847781	1.048144
0.636908	1.852495	0.812475
1.548406	1.118754	1.009065
0.974483	1.234276	0.681206
0.722207	1.856335	0.938675
1.175076	1.839712	0.430219
1.224173	1.336102	
	1.65601	
	1.462145	
	2.920536	
	1.760123	
	1.980637	
	1.798561	
	1.787229	
	1.883486	
	0.857674	
	1.337355	
	1.530206	
	1.333691	
	1.258476	
	1.468759	
