# Supplementary material for: PHD1-dependent hydroxylation of RepoMan (CDCA2) on P604 modulates the control of mitotic progression
Source: eLife. 2026 Jun 25;14:RP108131. doi: 10.7554/eLife.108131 (PMC13299607; doi:10.7554/eLife.108131)
Supplement: Figure 3—figure supplement 1—source data 2. [file elife-108131-fig3-figsupp1-data2.pdf]

Figure 3- figure supplement 1-source data 2

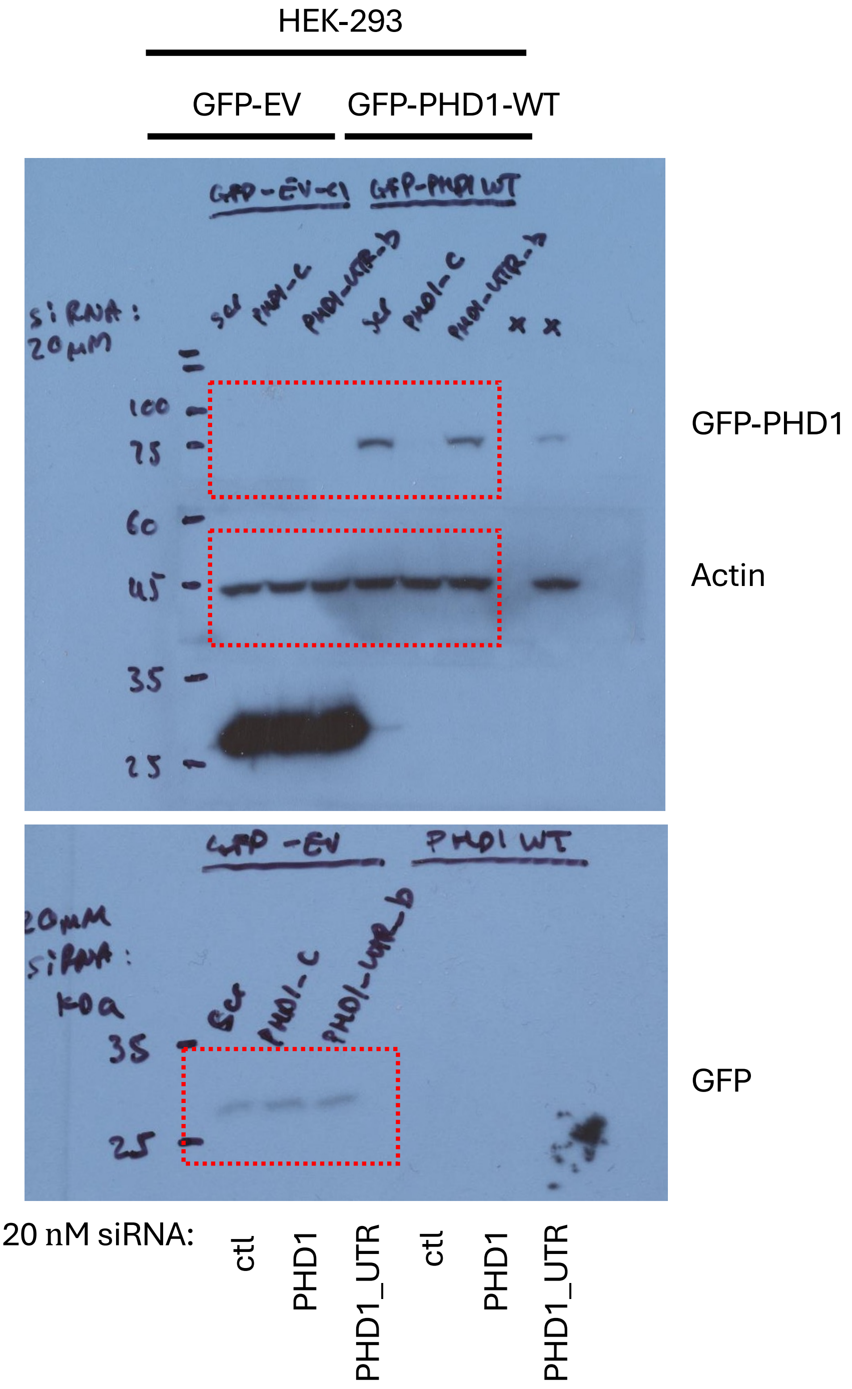

The lower membrane was washed with TBS_T and re-probing with GFP to get cleaner / less exposed GFP bands in the empty vector lanes and those are show in Figure 3- figure supplement 1_B.
