# Supplementary material for: PHD1-dependent hydroxylation of RepoMan (CDCA2) on P604 modulates the control of mitotic progression
Source: eLife. 2026 Jun 25;14:RP108131. doi: 10.7554/eLife.108131 (PMC13299607; doi:10.7554/eLife.108131)
Supplement: Figure 4—source data 2. [file elife-108131-fig4-data2.pdf]

Figure 4 -source data 2

A

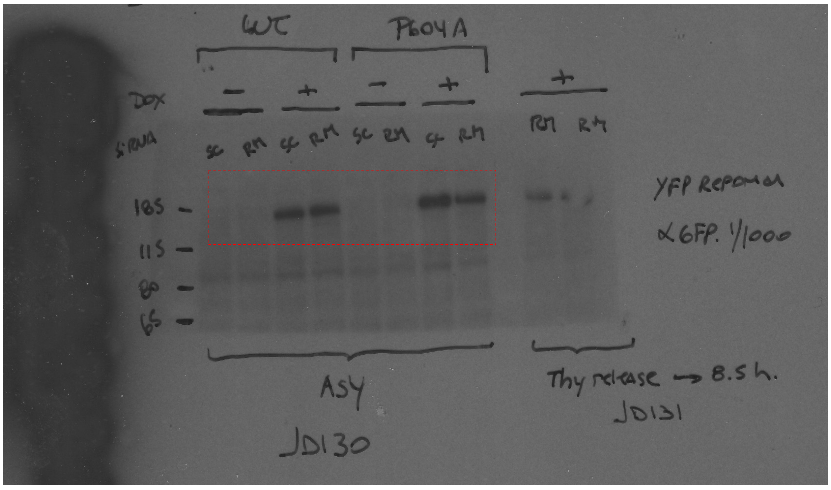

GFP

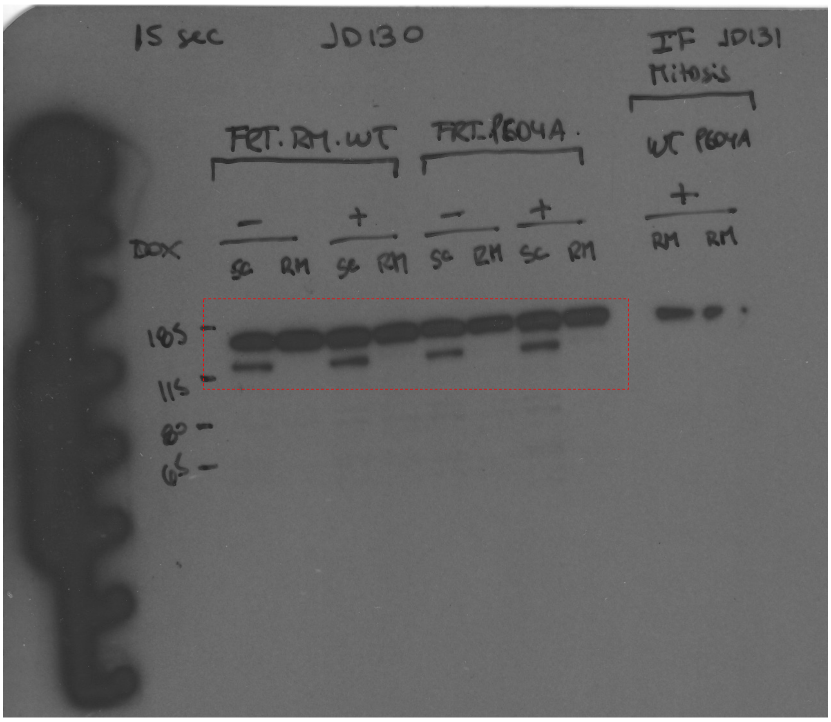

RepoMan

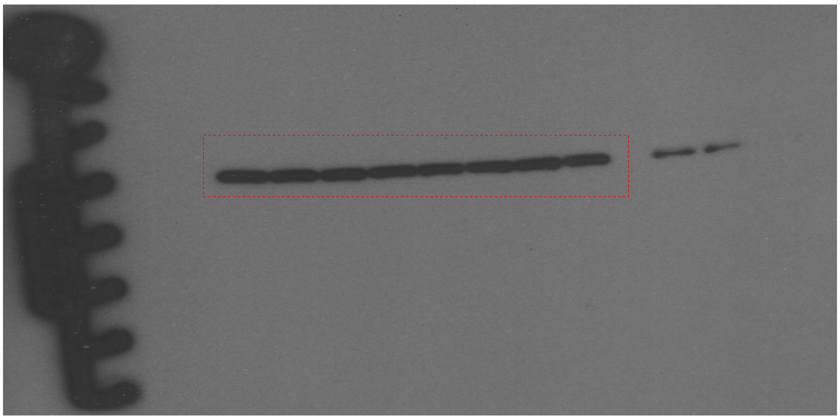

Actin

Figure 4A: Same membrane was developed first with GFP antibody and then with CDCA2 Antibody (Repoman).
