# Supplementary material for: PHD1-dependent hydroxylation of RepoMan (CDCA2) on P604 modulates the control of mitotic progression
Source: eLife. 2026 Jun 25;14:RP108131. doi: 10.7554/eLife.108131 (PMC13299607; doi:10.7554/eLife.108131)
Supplement: Figure 4—source data 3. [file elife-108131-fig4-data3.pdf]

Figure 4 -source data 3

Experiment	Condition	Distribution Foci		Distribution Diffuse		
		Number of cells	Percentage (%)	Number of cells	Percentage (%)	Total number of cells
**1**	si RM - DOX	3	20	12	80	15
	siRM + DOX (WT)	12	80	3	20	15
	si RM + DOX (P604A)	10	50	10	50	20
**2**	si RM - DOX	2	22.22222222	7	77.77777778	9
	siRM + DOX (WT)	6	66.66666667	3	33.33333333	9
	si RM + DOX (P604A)	5	50	5	50	10
**3**	si RM - DOX	3	25	9	75	12
	siRM + DOX (WT)	11	91.66666667	1	8.333333333	12
	si RM + DOX (P604A)	1	8.333333333	11	91.66666667	12
**4**	si RM - DOX	0	0	10	100	10
	siRM + DOX (WT)	8	80	2	20	10
	si RM + DOX (P604A)	4	40	6	60	10
