# Supplementary material for: PHD1-dependent hydroxylation of RepoMan (CDCA2) on P604 modulates the control of mitotic progression
Source: eLife. 2026 Jun 25;14:RP108131. doi: 10.7554/eLife.108131 (PMC13299607; doi:10.7554/eLife.108131)
Supplement: Figure 5—source data 2. [file elife-108131-fig5-data2.pdf]

Figure 5 -source data 2

C

Input IP

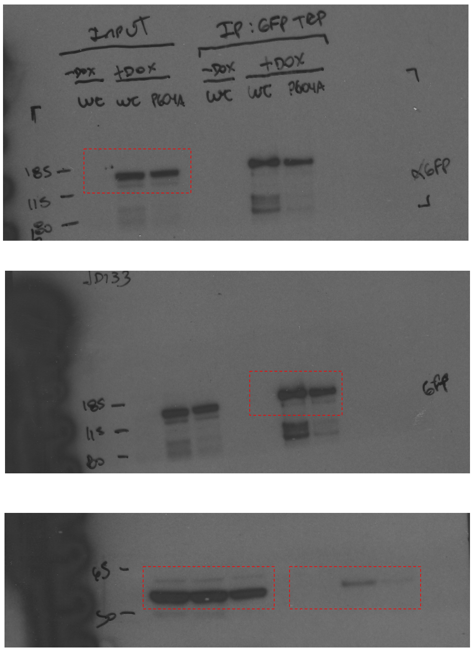

GFP

GFP

B56γ

D

Input IP

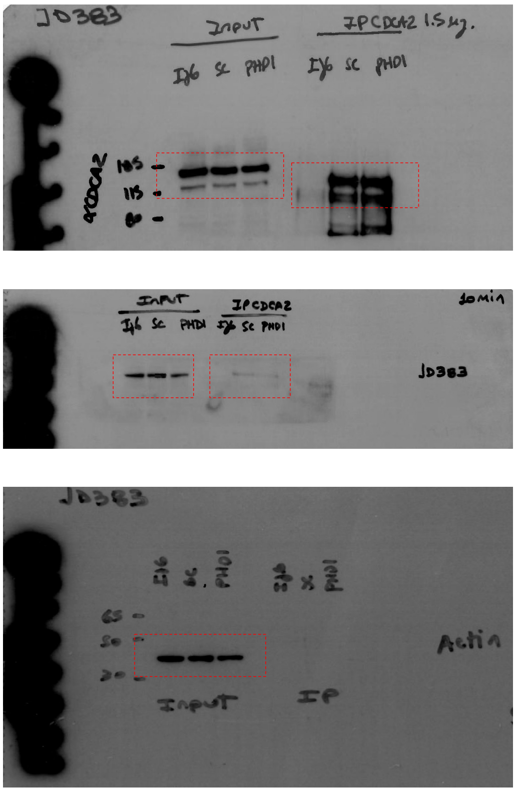

RepoMan

B56γ

Actin

E

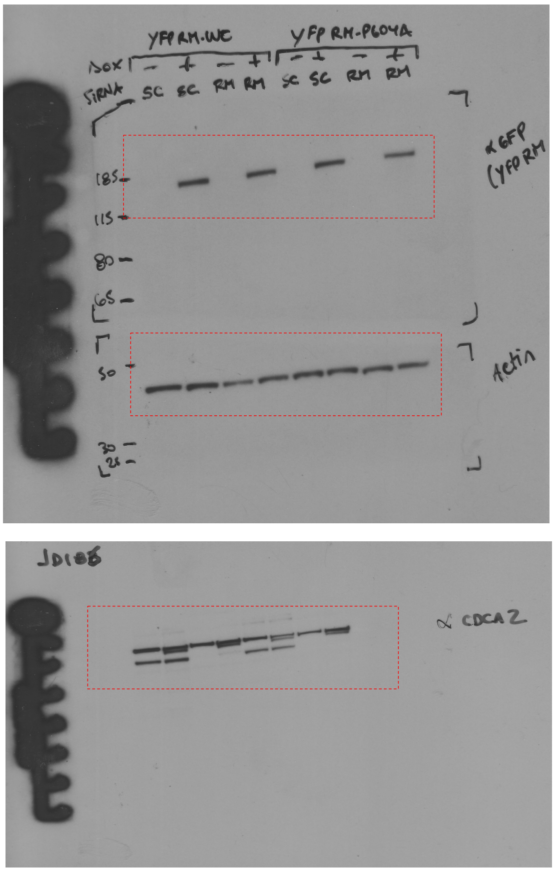

GFP

Actin

RepoMan

Figure 5E:Same membrane was developed first with GFP antibody and then with CDCA2 Antibody (Repoman)
