# Supplementary material for: PHD1-dependent hydroxylation of RepoMan (CDCA2) on P604 modulates the control of mitotic progression
Source: eLife. 2026 Jun 25;14:RP108131. doi: 10.7554/eLife.108131 (PMC13299607; doi:10.7554/eLife.108131)
Supplement: Figure 5—source data 4. [file elife-108131-fig5-data4.pdf]

Figure 5 - source data 4

Table A

WT	P604A
0.382013	0.184494
2.845382	0.113131
1.653171	0.040809
3.025067	0.628805
1.367205	0.88311
0.149512	0.257074
0.575781	0.201758
0.333396	0.685549
0.790974	0.422058
	0.231086
0.700443	0.25443
0.947364	-0.01204
1.895165	0.082602
-0.02585	0.91483
0.217984	0.88787
1.438983	0.522494
0.472651	1.118349
1.558113	0.358472
0.236114	0.126145
0.436534	0.935984
0.9181	0.159838
1.671529	1.158703
	0.568616
1.085211	0.381152
0.929098	0.943291
1.158373	0.319277
0.942487	-0.1416
0.729352	1.15168
0.373	0.278449
0.445106	0.070144
0.803031	0.03269
0.480444	0.94049
0.991068	0.319211
0.893874	-0.04565
0.514129	0.297062
1.04877	0.222379
0.686824	1.169893
0.489827	0.811418
3.160283	0.391603
1.679494	0.868989

1.845755	1.792
0.4644992	2.044
1.7726898	1.24
0.8452043	-0.17
2.5988323	0.197
0.3677532	0.242
0.1350568	0.932
0.8085126	-0.33
1.0550522	0.206
0.7116502	0.013
1.1589974	0.031
0.3708741	0.334
0.6728299	0.519
1.0454919	2.511
2.0041091	-0.06
0.3443329	-0.11
0.7734894	0.139
1.7544757	0.159
1.1146317	0.708
0.1557624	0.552

Table B

WT	P604A
1118.768	1615.953
1825.518	2166.74
2156.714	731.062
1693.546	2510.669
1505.566	1881.175
3023.273	2301.058
1765.134	2114.603
1559.032	2509.984
1438.412	2418.294
	1964.355
1606.799	2485.96
2226.264	4285.482
3105.972	1621.018
464.048	3985.764
797.5	4613.297
2938.802	3424.226
1334.173	5126.46

3302.518	3332.136
2157.601	794.839
2054.811	5497.501
1420.253	2067.607
5849.461	2151.314
	2353.464
2826.938	1909.35
45.301	6591.143
3172.94	4537.455
3423.323	2573.254
2097.455	4998.547
3560.296	5122.853
4757.717	3886.582
1157.545	4192.674
3717.204	2825.341
2880.673	6262.861
6605.251	3998.237
3689.002	2098.319
4658.459	3485.436
3794.681	1914.516
5326.024	3239.928
4768.19	880.926
3618.472	3735.652
5573.448	2610.298
1859.619	468.609
3686.765	1005.197
3458.858	2245.288
2842.168	2389.238
1446.327	2170.969
3735.933	4066.413
6736.537	1194.509
3720.538	2666.717
6609.468	2227.699
4615.945	3336.706
4033.608	5803.401
1713.346	714.227
2895.878	524.309
1646.284	2913.716
6637.744	1908.486
4327.121	2890.513
1228.222	3844.408
4642.506	1948.642
4481.239	2650.3
