# Supplementary material for: PHD1-dependent hydroxylation of RepoMan (CDCA2) on P604 modulates the control of mitotic progression
Source: eLife. 2026 Jun 25;14:RP108131. doi: 10.7554/eLife.108131 (PMC13299607; doi:10.7554/eLife.108131)
Supplement: Figure 6—source data 1. [file elife-108131-fig6-data1.pdf]

Figure 6 -source data 1

Experiment	condition	Number of unaligned bipolar cells	Number of aligned bipolar cells	Number of total bipolar cells	% unaligned bipolar cells	% aligned bipolar cells
1	**CtL**	39	43	82	47.56097561	52.43902439
	**WT**	53	36	89	59.5505618	40.4494382
	**P604A**	60	19	79	75.94936709	24.05063291
2	**CtL**	32	46	78	41.02564103	58.97435897
	**WT**	38	40	78	48.71794872	51.28205128
	**P604A**	63	17	80	78.75	21.25
3	**WT**	15	14	29	51.72413793	48.27586207
	**P604A**	19	9	28	67.85714286	32.14285714
