# Supplementary material for: PHD1-dependent hydroxylation of RepoMan (CDCA2) on P604 modulates the control of mitotic progression
Source: eLife. 2026 Jun 25;14:RP108131. doi: 10.7554/eLife.108131 (PMC13299607; doi:10.7554/eLife.108131)
Supplement: Figure 6—source data 2. [file elife-108131-fig6-data2.pdf]

Figure 6- source data 2

Experiment		RepoMan-WT	RepoMan-P604A
1	Total cells	23	18
	Defects (n of cells)	6	10
	Normal (n of cells)	17	8
	%Defects	26.08695652	55.55555556
	%Normal	73.91304348	44.44444444
2		RepoMan-WT	RepoMan-P604A
	Total cells	15	16
	Defects (n of cells)	5	10
	Normal (n of cells)	10	6
	%Defects	33.33333333	62.5
	%Normal	66.66666667	37.5
3		RepoMan-WT	RepoMan-P604A
	Total cells	19	21
	Defects (n of cells)	3	11
	Normal (n of cells)	16	10
	%Defects	15.78947368	52.38095238
	%Normal	84.21052632	47.61904762
