# Supplementary material for: PHD1-dependent hydroxylation of RepoMan (CDCA2) on P604 modulates the control of mitotic progression
Source: eLife. 2026 Jun 25;14:RP108131. doi: 10.7554/eLife.108131 (PMC13299607; doi:10.7554/eLife.108131)
Supplement: Peer review history

# Editor report: eLife Assessment

**DOI:** 10.7554/eLife.108131.3.sa0

**Authors:** Qing Zhang

**Affiliations:** https://ror.org/05byvp690 The University of Texas Southwestern Medical Center United States

**Keywords:** Compelling, Important

This **compelling** work describes how the cell cycle-regulating phosphatase subunit, RepoMan, is regulated by the oxygen-dependent, metabolite-sensing hydroxylase PHD1. The characterisation of how proline hydroxylation alters signalling at the molecular and cellular level provides **important** evidence to enhance our understanding of how 2-oxoglutarate-dependent dioxygenases influence the cell cycle and mitosis.

# Referee report: Reviewer #1 (Public review)

**DOI:** 10.7554/eLife.108131.3.sa1

Summary:

The study by Druker et al. shows that siRNA depletion of PHD1, but not PHD2, increases H3T3 phosphorylation in cells arrested in prometaphase. Additionally, the expression of wild-type RepoMan, but not the RepoMan P604A mutant, restored normal H3T3 phosphorylation localization in cells arrested in prometaphase. Furthermore, the study demonstrates that expression of the RepoMan P604A mutant leads to defects in chromosome alignment and segregation, resulting in increased cell death. These data support a role for PHD1-mediated prolyl hydroxylation in controlling progression through mitosis. This occurs, at least in part, by hydroxylating RepoMan at P604, which regulates its interaction with PP2A during chromosome alignment.

Strengths:

The data support most of the conclusions made.

Comments on revisions:

Actually, I am still concerned that PHD1 binds to RepoMan endogenously and directly. Furthermore, the authors have not yet provided genetic evidence demonstrating that PHD1 controls progression through mitosis by catalyzing the hydroxylation of RepoMan.

# Referee report: Reviewer #2 (Public review)

**DOI:** 10.7554/eLife.108131.3.sa2

Summary:

This is a concise and interesting article on the role of PHD1-mediated proline hydroxylation of proline residue 604 on RepoMan and its impact on RepoMan-PP1 interactions with phosphatase PP2A-B56 complex leading to dephosphorylation of H3T3 on chromosomes during mitosis. Through biochemical and imaging tools, the authors delineate a key mechanism in regulation of progression of the cell cycle. The experiments performed are conclusive with well-designed controls.

Strengths:

The authors have utilized cutting edge imaging and colocalization detection technologies to infer the conclusions in the manuscript.

Weaknesses:

Lack of in vitro reconstitution and binding data.

Comments on revisions:

Thank you, authors, for providing the statistics and siRNA validations. While I maintain that the manuscript's claims can benefit a lot from the in vitro experiments and that a Pro-Ala mutation may not be a good mimic for Pro-hydroxylation, I understand the authors' reservations and restrictions regarding the new experiments. Despite the lacunae, the manuscript is a good advance for the field.

# Referee report: Reviewer #3 (Public review)

**DOI:** 10.7554/eLife.108131.3.sa3

Summary:

The manuscript is a comprehensive molecular and cell biological characterisation of the effects of P604 hydroxylation by PHD1 on RepoMan, a regulatory subunit of the PPIgamma complex. Conclusions are generally supported by results. Overall, a timely study that demonstrates the interplay between hydroxylase signalling and the cell cycle. The study extends the scope of prolyl hydroxylase signalling beyond canonical hypoxia pathways, providing a concrete example of hydroxylation regulating PP1 holoenzyme composition and function during mitosis.

The work would benefit from additional biochemical validation of direct targeting to characterise the specificity and mode of recognition, but this is beyond the scope of the study.

Strengths:

Compelling data, characterisation of how P604 hydroxylation induces the interaction between RepoMan and a phosphatase complex, resulting in loading of RepoMan on Chromatin. Knockdown of PHD1 mimics the disruption of the complex and loss of the regulation of the hydroxylation site by PHD1, resulting in mitotic defects.

# Author comment: Author response

**DOI:** 10.7554/eLife.108131.3.sa4

**Authors:** Jimena Druker, Hao Jiang, Dilem Shakir, Fraser Child, Vanesa Alvarez, Melpomeni Platani, Andrea Corno, Constance Alabert, Adrian T Saurin, Jason R Swedlow, Sonia Rocha, Angus I Lamond

**Affiliations:** University of Dundee Dundee United Kingdom; University of Dundee Dundee United Kingdom; University of Liverpool Liverpool United Kingdom; University of Liverpool Liverpool United Kingdom; University of Dundee Dundee United Kingdom; University of Dundee Dundee United Kingdom; University of Dundee Dundee United Kingdom; Gene Expression and Regulation, University of Dundee Dundee United Kingdom; University of Dundee Dundee United Kingdom; University of Dundee Dundee United Kingdom; University of Liverpool Liverpool United Kingdom; University of Dundee Dundee United Kingdom

The following is the authors’ response to the original reviews.

**Public Reviews:**

**Reviewer #1 (Public review):**

Summary:

The study by Druker et al. shows that siRNA depletion of PHD1, but not PHD2, increases H3T3 phosphorylation in cells arrested in prometaphase. Additionally, the expression of wild-type RepoMan, but not the RepoMan P604A mutant, restored normal H3T3 phosphorylation localization in cells arrested in prometaphase. Furthermore, the study demonstrates that expression of the RepoMan P604A mutant leads to defects in chromosome alignment and segregation, resulting in increased cell death. These data support a role for PHD1-mediated prolyl hydroxylation in controlling progression through mitosis. This occurs, at least in part, by hydroxylating RepoMan at P604, which regulates its interaction with PP2A during chromosome alignment.

Strengths:

The data support most of the conclusions made. However, some issues need to be addressed.

Weaknesses:

(1) Although ectopically expressed PHD1 interacts with ectopically expressed RepoMan, there is no evidence that endogenous PHD1 binds to endogenous RepoMan or that PHD1 directly binds to RepoMan.

We do not fully agree that this comment is accurate - the implication is that we only show interaction between two exogenously expressed proteins, i.e. both exogenous PHD1 and RepoMan, when in fact we show that tagged PHD1 interacts with endogenous RepoMan. The major technical challenge here is the well-known difficulty of detecting endogenous PHD1 in such cell lines. We agree that co-IP studies do not prove that this interaction is direct and never claim to have shown this, though we do feel that a direct interaction is most likely, albeit not proven.

(2) There is no genetic evidence indicating that PHD1 controls progression through mitosis by catalyzing the hydroxylation of RepoMan.

We agree that our current study is primarily a biochemical and cell biological study, rather than a genetic study. Nonetheless, similar biochemical and cellular approaches have been widely used and validated in previous studies in mechanisms regulating cell cycle progression and we are confident in the conclusions drawn based on the data obtained so far.

(3) Data demonstrating the correlation between dynamic changes in RepoMan hydroxylation and H3T3 phosphorylation throughout the cell cycle are needed.

We agree that it will be very interesting to analyse in more detail the cell cycle dynamics of RepoMan hydroxylation and H3T3 phosphorylation - along with other cell cycle parameters. We view this as outside the scope of our present study and are actively engaged in raising the additional funding needed to pursue such future experiments.

(4) The authors should provide biochemical evidence of the difference in binding ability between RepoMan WT/PP2A and RepoMan P604A/PP2A.

Here again we agree that it will be very interesting to analyse in future the detailed binding interactions between wt and mutant RepoMan and other interacting proteins, including PP2A. We show reduced interaction in cells by PLA (Figure 5A) and in biochemical analysis (Figure 5C). More in vitro analysis is, in our view, outside the scope of our present study and we are actively engaged in raising the additional funding needed to pursue such future experiments.

(5) PHD2 is the primary proline hydroxylase in cells. Why does PHD1, but not PHD2, affect RepoMan hydroxylation and subsequent control of mitotic progression? The authors should discuss this issue further.

We agree with the main point underpinning this comment, i.e., that there are still many things to be learned concerning the specific roles and mechanisms of the different PHD enzymes in vivo. We address this in the Discussion section and look forward to addressing these questions experimentally in future studies.

**Reviewer #2 (Public review):**

Summary:

This is a concise and interesting article on the role of PHD1-mediated proline hydroxylation of proline residue 604 on RepoMan and its impact on RepoMan-PP1 interactions with phosphatase PP2A-B56 complex leading to dephosphorylation of H3T3 on chromosomes during mitosis. Through biochemical and imaging tools, the authors delineate a key mechanism in the regulation of the progression of the cell cycle. The experiments performed are conclusive with well-designed controls.

Strengths:

The authors have utilized cutting-edge imaging and colocalization detection technologies to infer the conclusions in the manuscript.

Weaknesses:

Lack of in vitro reconstitution and binding data.

We agree that it will be very interesting to pursue in vitro reconstitution studies and detailed binding data. We view this as outside the scope of our present study and are actively engaged in raising the additional funding needed to pursue such future experiments. We do provide in vitro hydroxylation data in our accompanying manuscript by Jiang et al, 2025 Elife.

**Reviewer #3 (Public review):**

Summary:

The manuscript is a comprehensive molecular and cell biological characterisation of the effects of P604 hydroxylation by PHD1 on RepoMan, a regulatory subunit of the PPIgamma complex. The identification and molecular characterisation of the hydroxylation site have been written up and deposited in BioRxiv in a separate manuscript. I reviewed the data and came to the conclusion that the hydroxylation site has been identified and characterised to a very high standard by LC-MS, in cells and in vitro reactions. I conclude that we should have no question about the validity of the PHD1-mediated hydroxylation.

In the context of the presented manuscript, the authors postulate that hydroxylation on P604 by PHD1 leads to the inactivation of the complex, resulting in the retention of pThr3 in H3.

Strengths:

Compelling data, characterisation of how P604 hydroxylation is likely to induce the interaction between RepoMan and a phosphatase complex, resulting in loading of RepoMan on Chromatin. Loss of the regulation of the hydroxylation site by PHD1 results in mitotic defects.

Weaknesses:

Reliance on a Proline-Alanine mutation in RepoMan to mimic an unhydroxylatable protein. The mutation will introduce structural alterations, and inhibition or knockdown of PHD1 would be necessary to strengthen the data on how hydroxylates regulate chromatin loading and interactions with B56/PP2A.

We do not agree that we rely solely on analysis of the single site pro-ala mutant in RepoMan for our conclusions, since we also present a raft of additional experimental evidence, including knock-down data and experiments using both fumarate and FG. We would also reference the data we present on RepoMan in the parallel study by Jiang et al, which has also published in eLife (https://doi.org/10.7554/eLife.108128.1). Of course, we agree with the reviewer that even although the mutant RepoMan features only a single amino acid change, this could still result in undetermined structural effects on the RepoMan protein that could conceivably contribute, at least in part, to some of the phenotypic effects observed. We now provide evidence in the current revision (new Figure 5D) that reduced interaction between RepoMan and B56gamma/PP2A is also evident when PHD1 is depleted from cells.

**Recommendations for the authors:**

**Reviewer #2 (Recommendations for the authors):**

(1) The manuscript can benefit from improved quality of writing and avoidance of grammatical errors.

We have checked through the manuscript again and corrected any mistakes we have encountered in the Current revision.

(2) Although the data in the manuscript is compelling, it is difficult to rule out indirect effects in the interactions. Hence, in vitro binding assays with purified proteins are important to validate the findings, along with in vitro reconstitution of phosphatase activity.

It is possible that cofactors and / or additional PTMs are required to promote these interactions in vivo. We have provided in vitro hydroxylation analysis and the additional experiments suggested will be the subject of follow-on future studies.

(3) Proline to alanine is a drastic mutation in the amino acid backbone. The authors could purify PHD1 and reconstitute P604 hydroxylation to show if it performs as expected.

This is likely to be a challenging experiment technically, given that RepoMan is a component of multiple distinct complexes, some of which are dynamic. We did not feel able to address this within the scope of the current study.

(4) The confocal images showing the overlap of two fluorescent signals need to show some sort of quantification and statistics to prove that the overlap is significant.

We now provide Pearson correlation measurements for Figure 2A in new Figure 2B in the Current revision.

(5) Kindly provide a clearer panel for the Western blot of H3T3ph in Figure 3c.

We have now included a new panel for this Figure in the Current revision.

(6) Kindly also include the figures for validation of siRNAs used in the study

We have added this throughout in supplementary figures.

**Reviewer #3 (Recommendations for the authors):**

(1) The authors have shown that PHD1 and RepoMan interact; can the interaction be "trapped" by the addition of DMOG? Generally, hydroxylase substrates can be trapped, which would add an additional layer of confidence that PHD1 and RepoMan form an enzyme-substrate complex.

This is something we are planning to do for follow-up studies using the established methods from the von Kriesgheim laboratory.

(2) How does P604A mutation affect the interaction with PHD1? One would expect a reduction in interaction.

Another interesting point we are planning to investigate in the future.

(3) The effects of expression of the wt and P604A mutant repoman are well-characterised. Could the authors check the effects of overexpressing PHD1 and deadPHD1, inhibition on the mitosis/H3 phosphorylation? My concerns are that a P-A mutation will disrupt the secondary structure, and although it is a good tool, data should be backed up by increasing/decreasing the hydroxylation of RepoMan over the mutation. Repeat some of the most salient experiments where the P604A mutation has been used and modulate the hydP604 by modulating PHD1 activity/expression (such as Chromatin interaction, PLA assay, B56gamma interaction, H3 phosphorylation localisation, Monastrol release, etc.)

We agree, the PA mutant can potentially affect the protein structure. In our manuscript we have provided pH3 analysis for PHD inhibition using siRNA, FG4592 and Fumarate. In the Current revision ee also data showing that depletion of PHD1 results in a reduction in interaction between RepoMan and B56gamma/PP2A. This is now presented in new figure 5D.

(4) I also have a general question, as a point of interest, as the interaction between PHD1 and RepoMan appears to be cell cycle dependent, is it possible that the hydroxylation status cycles as well? Could this explain how some sub-stochiometric hydroxylation events observed may be masked by assessing unsynchronised cells in bulk?

Indeed, a very good question. We believe this is an interesting question for follow up studies. Given our previous publication showing phosphorylation of PHD1 by CDKs alters substrate binding (Ortmann et al, 2016 JCS), this is our current hypothesis.
